# The C-terminal of CASY-1/Calsyntenin regulates GABAergic synaptic transmission at the *Caenorhabditis elegans* neuromuscular junction

**DOI:** 10.1371/journal.pgen.1007263

**Published:** 2018-03-12

**Authors:** Shruti Thapliyal, Amruta Vasudevan, Yongming Dong, Jihong Bai, Sandhya P. Koushika, Kavita Babu

**Affiliations:** 1 Department of Biological Sciences, Indian Institute of Science Education and Research (IISER) Mohali, Knowledge City, Sector 81, SAS Nagar, Manauli, Punjab, India; 2 Department of Biological Sciences, Tata Institute of Fundamental Research, Colaba, Mumbai, India; 3 Basic Sciences Division, Fred Hutchinson Cancer Research Center, Seattle, WA 98109 and Department of Biochemistry, University of Washington, Seattle, WA, United Sttaes of America; Katholieke Universiteit Leuven, BELGIUM

## Abstract

The *C*. *elegans* ortholog of mammalian calsyntenins, CASY-1, is an evolutionarily conserved type-I transmembrane protein that is highly enriched in the nervous system. Mammalian calsyntenins are strongly expressed at inhibitory synapses, but their role in synapse development and function is still elusive. Here, we report a crucial role for CASY-1 in regulating GABAergic synaptic transmission at the *C*. *elegans* neuromuscular junction (NMJ). The shorter isoforms of CASY-1; CASY-1B and CASY-1C, express and function in GABA motor neurons where they regulate GABA neurotransmission. Using pharmacological, behavioral, electrophysiological, optogenetic and imaging approaches we establish that GABA release is compromised at the NMJ in *casy-1* mutants. Further, we demonstrate that CASY-1 is required to modulate the transport of GABAergic synaptic vesicle (SV) precursors through a possible interaction with the SV motor protein, UNC-104/KIF1A. This study proposes a possible evolutionarily conserved model for the regulation of GABA synaptic functioning by calsyntenins.

## Author summary

GABA acts as a major inhibitory neurotransmitter in both vertebrate and invertebrate nervous systems. Despite the potential deregulation of GABA signaling in several neurological disorders, our understanding of the genetic factors that regulate GABAergic synaptic transmission has just started to evolve. Here, we identify a role for a cell adhesion molecule, CASY-1, in regulating GABA signaling at the *C*. *elegans* NMJ. We show that the mutants in *casy-1* have reduced number of GABA vesicles at the synapse resulting in less GABA release from the presynaptic GABAergic motor neurons. Further, we show that the shorter isoforms of the *casy-1* gene; *casy-1b* and *casy-1c* that carry a potential kinesin-motor binding domain are responsible for maintaining GABAergic signaling at the synapse. We show a novel interaction of the CASY-1 isoforms with the C- terminal of the UNC-104/KIF1A motor protein that mediates the trafficking of GABAergic synaptic vesicle precursors to the synapse, thus maintaining normal inhibitory signaling at the NMJ.

## Introduction

A remarkable feature of the nervous system is the specific connections between neurons, which allows for orchestrated neural networks and circuitry of the brain. Many cell adhesion molecules (CAMs) are concentrated at synaptic sites in neuronal axons and dendrites, serving as dynamic regulators of synaptic function. Considering the complexity of the nervous system, tightly controlled spatial and temporal regulation of several different classes of CAMs is essential. Existing literature suggests that neuronal CAMs are not only important for adhesion but are also required for various aspects of synapse development and function [1–16].

The *C*. *elegans casy-1* is an ortholog of mammalian Calsyntenin genes. Calsyntenins are type-I transmembrane proteins characterized by the presence of two cadherin-like tandem repeats, an LG/LNS domain in the extracellular region and an intracellular region that carries two kinesin light-chain binding domains [17, 18]. All these regions are conserved in the three human Calsyntenin genes; *clstn1*, *clstn2* and *clstn3* as well as in the sole *C*. *elegans* calsyntenin ortholog, *casy-1*. Calsyntenins are highly expressed in the mammalian central nervous system [17, 19]. Similarly, *C*. *elegans* CASY-1 was also observed in most head neurons and some other non-neuronal tissues like intestine and gonadal sheath cells [20, 21]. Mammalian calsyntenins show a high degree of spatial and temporal regulation of expression, indicating possible diversity of functions. For example, calsyntenins associate with the scaffold proteins X11/X11L, which in turn associates with the amyloid precursor proteins (APP) forming a tripartite complex in the brain [22]. CLSTN1 has been shown to interact with the kinesin light-chain protein resulting in blocking the transport of APP-containing vesicles and thus causing more β- amyloid generation, suggesting a possible role of these molecules in the pathogenesis of Alzheimer’s disease [18, 23–27]. Developmentally, CLSTN1 has been shown to be required for trafficking of NMDA receptors at the synapse and is essential for neuronal maturation during early embryonic development [28]. Further, CLSTN1 also regulates axon branching and endosomal trafficking during sensory neuron formation [29]. More recently, CLSTN1 has been shown to mediate trafficking of axon guidance receptors at spinal cord choice points [30] and in regulating microtubule polarity during sensory axon arbor development [31]. In contrast to the roles of CLSTN1, which appears to mediate trafficking functions during nervous system development, CLSTN2 has been reported to be required for modulating synaptic plasticity. In a genome-wide screen for human hippocampus based-episodic memory genes, CLSTN2 was identified as a target that could be involved in human memory performance [32]. CLSTN2 knock-out mice are hyperactive with cognitive deficits due to reduced GABAergic neurotransmission [33]. The molecular basis of how CLSTN2 regulates GABA neurotransmission is however still unknown. CLSTN3 has been reported to act as a synaptic adhesion molecule that promotes excitatory and inhibitory synapse development in concert with neurexins [34]. In Zebrafish, the extracellular cadherin domains of calsyntenins have been shown to mediate homophilic adhesion [35]. However, despite all these studies that have allowed us to understand the functions of calsyntenins, we are still far from elucidating the molecular and physiological underpinnings of this molecule.

The calsyntenin ortholog in *C*. *elegans*, CASY-1 has also been found to be essential for multiple forms of learning [20, 21]. It has been shown that in salt chemotaxis learning, the LG/LNS domain in the extracellular region of CASY-1 is essential for memory formation. Functional rescue experiments by expressing human CLSTN2 in *C*. *elegans casy-1* mutants were able to rescue the learning defects, highlighting conservation of function.

In this study, we propose a role for CASY-1 in regulating GABA synaptic vesicle (SV) precursor transport and hence maintaining normal GABAergic neurotransmission at the *C*. *elegans* neuromuscular junction (NMJ). We show that the two shorter isoforms of CASY-1, that only have the conserved C-terminal region and lack the entire extracellular N-terminal region, function in GABAergic motor neurons to regulate GABA release by mediating the trafficking of GABA SV precursors via their interaction with the UNC-104 motor protein.

## Results

### Mutants in *casy-1* are hypersensitive to Aldicarb

The *C*. *elegans* NMJ is an extensively used model to understand various aspects of synapse development and function. The NMJ consists of body-wall muscles that receive synaptic inputs from both excitatory cholinergic and inhibitory GABAergic motor neurons. An intricate stability between the excitatory and inhibitory signaling is responsible for the sinusoidal locomotion in *C*. *elegans* and any defect in this balance could result in altered synaptic function (reviewed in [36, 37]).

**Fig 1:**
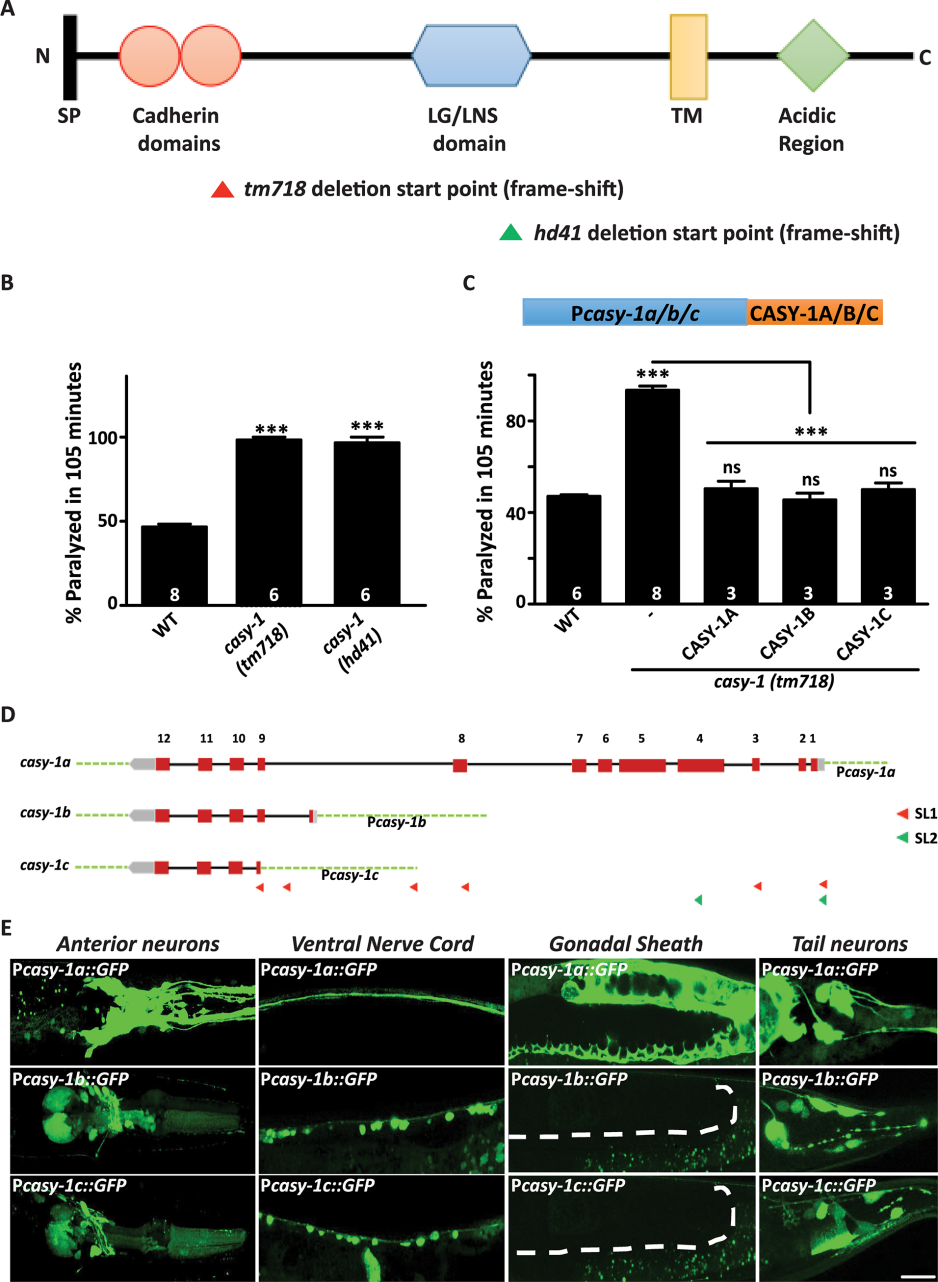
Mutants in *casy-1* are hypersensitive to Aldicarb. (A) A schematic representation of the CASY-1 protein showing the N-terminal signal peptide (SP), two-tandem cadherin repeats, LG/LNS domain, transmembrane region (TM) and cytosolic acidic region. The domains deleted in *casy-1 mutants* are indicated as triangles. The *tm718* and *hd41* alleles are putative null alleles as deletion starts in the N-terminal region and results in a frame-shift in both cases. (B) Aldicarb-induced paralysis in *casy-1* mutants was compared to wild-type (WT) animals. Both *casy-1* mutant alleles (*tm718* and *hd41*) are hypersensitive in the Aldicarb assays. Assays were done at least 6 times. (C) A schematic representing the transgenes used in the experiment. Expression of *casy-1* isoforms under their endogenous promoters completely rescues the Aldicarb hypersensitivity of *casy-1* mutant animals. In B and C, number of assays (~20 *C*. *elegans*/assay) is indicated for each genotype. Data are represented as mean ± S.E.M. (***p<0.0001 using one-way ANOVA and Bonferroni's Multiple Comparison Test) “ns” indicates not significant in all Figures. (D) Pictorial representation of the genomic locus of three isoforms. CASY-1B and CASY-1C are expressed by alternative promoters that exist in between the 8^th^ and 9^th^ intron of CASY-1A isoform, which is unusually long (~ 4000bp) and carries their own SL1 leader sequences. The location of promoter sequences utilized in the study are indicated. (E) Representative confocal images of transcriptional reporters of the three *casy-1* isoforms. Expression of GFP under isoform-specific promoters showed expression of *casy-1a* in most of the head neurons including amphid sensory neurons, in VNC, some tail neurons, in the intestine as well as in the gonadal sheath. *casy-1b* and *casy-1c* also showed expression in some head neurons, in the ventral cord motor neurons and some tail neurons but no expression in the gonadal sheath. Dotted lines indicate the position of gonadal sheath. Scale bar, 20μm.

Previously an RNAi screen was conducted using the acetylcholine esterase inhibitor, Aldicarb, to identify the function of cell adhesion molecules at the *C*. *elegans* NMJ [38]. The presence of Aldicarb causes acute paralysis due to accumulation of acetylcholine at the NMJ. Loss of function of genes that are necessary for synaptic function could cause either increased resistance or hypersensitivity to Aldicarb [39–42]. One of the positives from the screen was *casy-1*. To validate the results of RNAi screening, two different mutant alleles of *casy-1*, *tm718* and *hd41*, were obtained. Both alleles are putative null alleles as they carry deletions that start at the N- terminal region and result in frame-shift mutations ([21] and (illustrated in Fig 1A)). Both *casy-1(tm718)* and *casy-1(hd41)* showed significant hypersensitivity to Aldicarb suggesting neuromuscular signaling defects in these mutants (Fig 1B). All further experiments were performed using the *casy-1(tm718)* mutant allele.

The *casy-1* locus in *C*. *elegans* is predicted to encode three isoforms based on EST evidence [43]. CASY-1A, a 984 residue full-length protein contains all the conserved domains of mammalian calsyntenins (illustrated in Fig 1A). CASY-1B and CASY-1C are truncated proteins encoding 167 and 160 residues respectively and lack most of the N- terminal of the calsyntenin gene (illustrated in S1A Fig). Real-time qPCR experiments revealed that all three isoforms of *casy-1* are significantly reduced in the *casy-1(tm718)* mutant allele (S1B Fig).

An isoform-specific rescue of the *casy-1* mutant phenotype was then performed using each isoform with its native promoter (illustrated in Fig 1D and detailed in supplemental methods). Surprisingly, all three isoforms could fully rescue the Aldicarb hypersensitivity of *casy-1* mutants (Fig 1C) suggesting that (1) the promoter sequences used for each *casy-1* isoform appears to be functional and (2) all three isoforms could be functioning to regulate synaptic transmission at the NMJ.

As reported earlier [21], we observed strong expression of *casy-1a* in a lot of head neurons including the amphid sensory neurons, the Ventral Nerve Cord (VNC) and some tail neurons. Expression was also observed in somatic tissues like gonadal sheath and intestine (Fig 1E). To examine the expression pattern of *casy-1b* and *casy-1c*, transcriptional reporter lines expressing NLS-GFP under isoform-specific promoters were generated. Compared to *casy-1a*, which is highly enriched in the head and tail neurons, *casy-1b* and *casy-1c* show a more limited expression in the head neurons. However, both *casy-1b* and *casy-1c* are strongly expressed in the VNC motor neurons, which is not seen with *casy-1a*. The weak expression of *casy-1a* in the VNC belongs to the axonal processes from the head neurons that traverse along the entire length of the *C*. *elegans* body. No expression was observed in the gonadal sheath for the shorter isoforms (Fig 1E).

### CASY-1 is not required for motor neuron development

**Fig 2:**
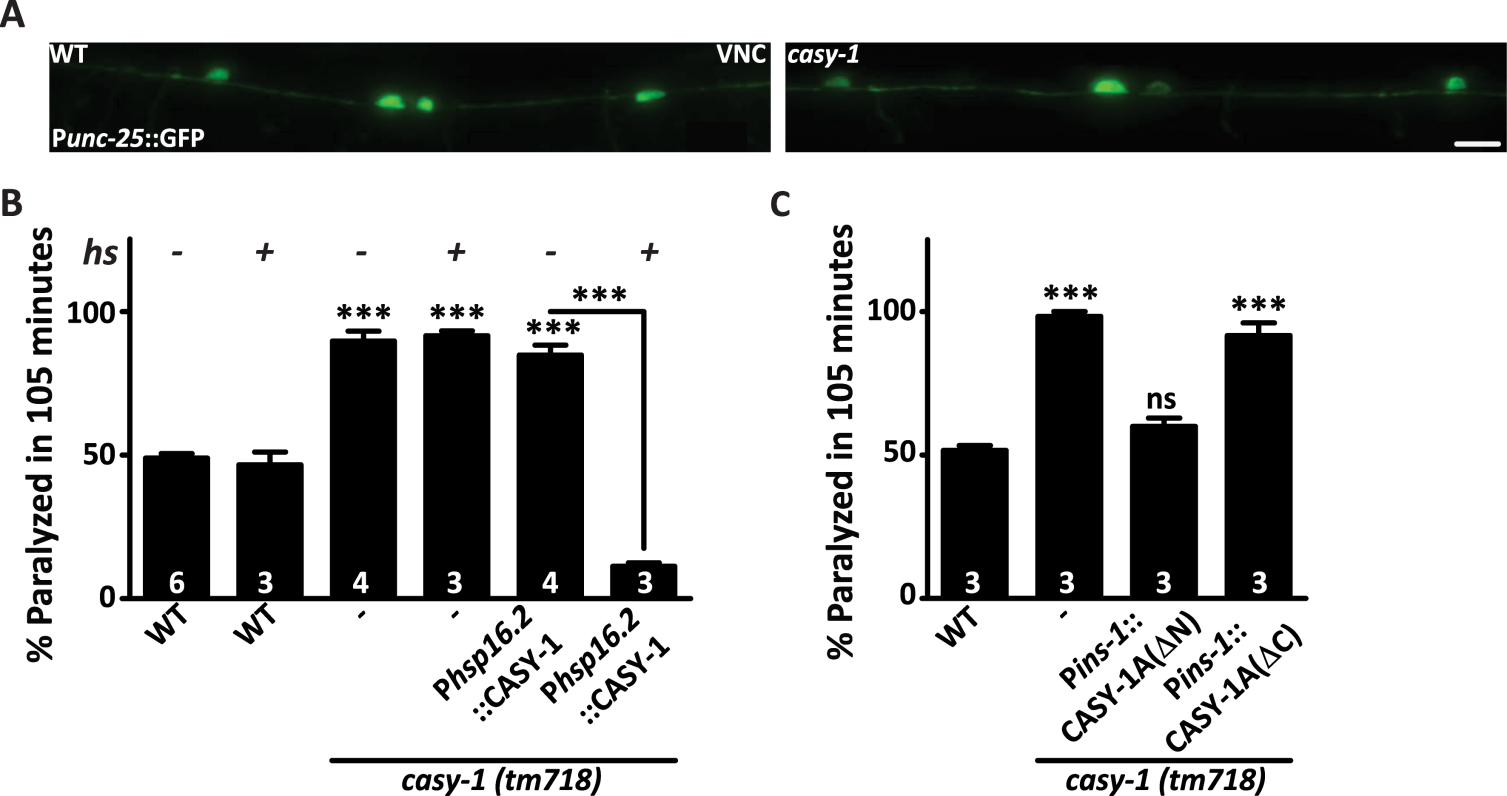
Motor neuron development is normal in *casy-1 mutants*. (A) Representative fluorescent images of WT and *casy-1* mutant animals expressing GFP in all GABAergic neurons (*juIs76* [P*unc-25*:: GFP]). The number of cell bodies and axonal commissures were largely normal in *casy-1* mutant animals (n-25) analyzed. Scale bar, 8μm. (B) CASY-1 functions in the mature nervous system to regulate synaptic transmission at the NMJ. Expression of CASY-1A isoform just three hours before the assay using a heat- shock promoter (*hsp16*.*2*) completely rescues the Aldicarb hypersensitivity phenotype seen in *casy-1* mutant animals. The number of assays (~20 *C*. *elegans*/assay) is indicated for each genotype. (C) The C-terminal of the CASY-1A isoform is required to regulate synaptic transmission at the NMJ. Transgenic lines expressing either the CASY-1A N-terminal (ΔC) or C-terminal (ΔN) alone expressed under *ins-1* promoter suggest that the CASY-1A C- terminal is sufficient to rescue the Aldicarb hypersensitivity in *casy-1* mutants. Assays were done 3 times as indicated in Figures (~20 *C*. *elegans*/ assay). Data are represented as mean ± S.E.M. (***p<0.0001 using one-way ANOVA and Bonferroni's Multiple Comparison Test). “ns” indicates not significant in all Figures.

Mammalian orthologs of CASY-1 have been shown to regulate various aspects of neuronal development [28–31, 34]. Since defects in the synthesis or release of either excitatory (acetylcholine) or inhibitory (GABA) neurotransmitter could result in Aldicarb hypersensitivity [41], we speculated that defects in cholinergic or GABAergic neuronal development could explain the Aldicarb hypersensitivity in *casy-1* mutants. To examine this, transgenic lines that express soluble markers driven by acetylcholine (ACh; *unc-17*) or GABA (*unc-25*) neuron-specific promoters were used. These transgenes were introduced in the *casy-1* mutant background. Analysis of the imaging data revealed no gross morphological defects in GABAergic or cholinergic motor neurons in *casy-1* mutants (Fig 2A and S2A Fig).

To further validate that CASY-1 is not essential for motor neuron developmental functions, a transgenic line that expressed the CASY-1A isoform conditionally under a heat shock promoter *hsp16*.*2* was used. Aldicarb hypersensitivity of *casy-1* mutants was completely rescued, and the *C*. *elegans* became resistant to Aldicarb when CASY-1A was expressed transiently in the mature nervous system (Fig 2B). These results further indicate the role of CASY-1 in regulating synaptic function in a mature nervous system rather than in neuronal development.

Since all three CASY-1 isoforms can completely rescue the Aldicarb hypersensitivity of *casy-1* mutants (Fig 1C), we decided to determine the region of CASY-1 that is required to regulate synaptic transmission at the NMJ. Two transgenic lines were utilized in which either the entire extracellular N-terminal region [CASY-1A (ΔN)] or the entire intracellular C-terminal region [CASY-1A (ΔC)] were removed [21]. Both constructs were expressed under the *ins-1* promoter in the ventral cord motor neurons [44]. Transgenes lacking the N-terminal domains could rescue the Aldicarb hypersensitivity of *casy-1* mutants, however transgenes lacking the C-terminal region could not rescue the Aldicarb defects (Fig 2C). These results indicate that the extracellular domains are dispensable for the synaptic function of CASY-1 at the NMJ. The requirement of just the cytoplasmic C-terminal region for regulating synaptic function at the NMJ further explains how all three CASY-1 isoforms, which are structurally different could rescue the Aldicarb defect of *casy-1* mutants. Since the entire C-terminus is conserved in all three CASY-1 isoforms (S2B Fig) it is conceivable that they utilize this region for their synaptic function when expressed in motor neurons.

### CASY-1 regulates synaptic transmission in GABAergic motor neurons

**Fig 3:**
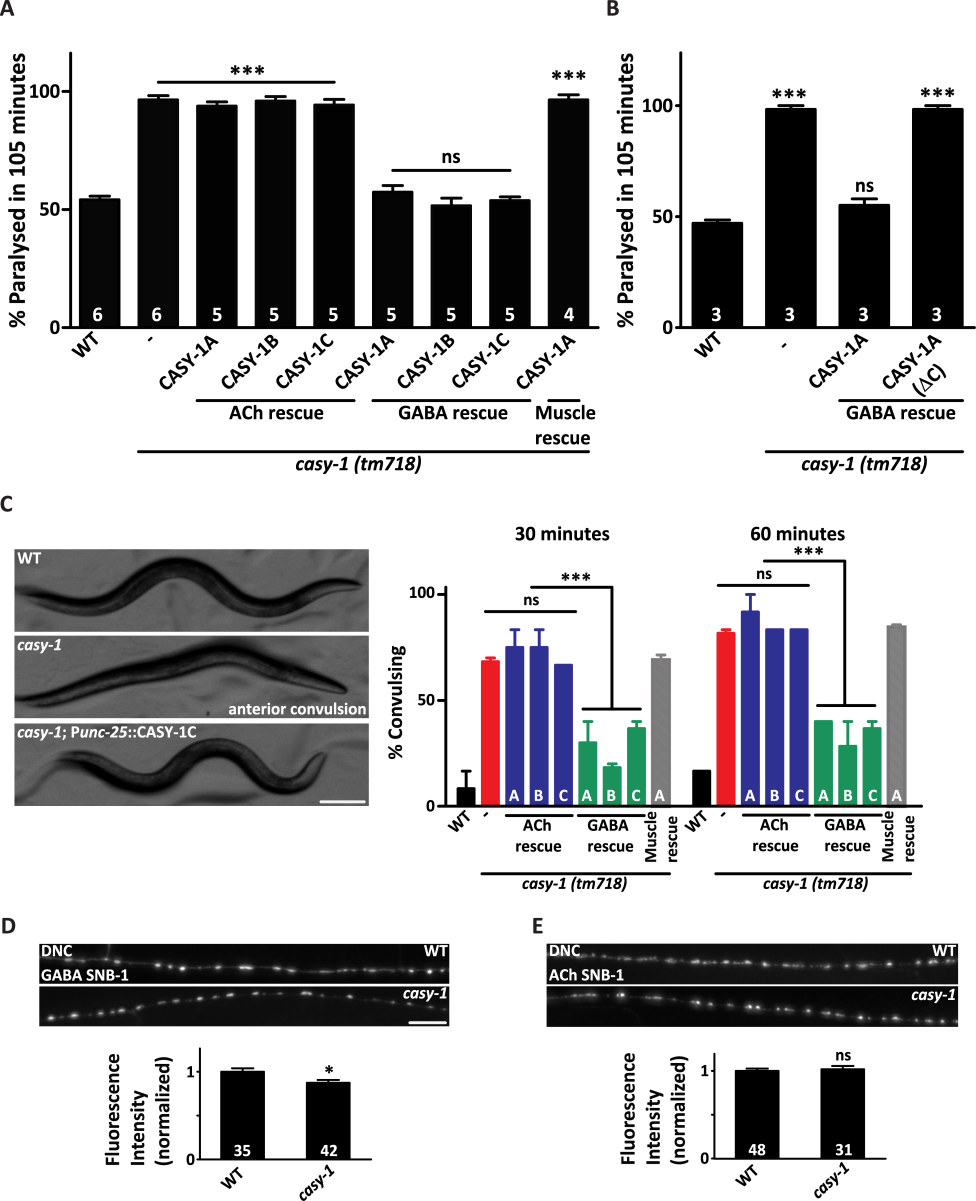
The *casy-1* isoforms functions in GABAergic neurons to regulate synaptic transmission. (A) Expression of CASY-1 isoforms using tissue specific promoters; ACh neurons (P*unc-17*), GABA neurons (P*unc-25*) and muscle (P*myo-3*) establishes that expressing CASY-1 in GABAergic neurons completely rescues the Aldicarb hypersensitivity in *casy-1* mutants. The number of assays (~20 *C*. *elegans*/assay) is indicated for each genotype. (B) The C-terminal of the CASY-1 isoforms functions in GABAergic neurons to regulate synaptic function. The CASY-1A isoform expressed under GABAergic promoter completely rescues the Aldicarb hypersensitivity in *casy-1* mutants. However, removing the entire C-terminal from the CASY-1A [CASY-1A (ΔC)] isoform does not rescue the Aldicarb hypersensitivity in *casy-1* mutants. The number of assays (~20 *C*. *elegans*/assay) is indicated for each genotype. (C) The *casy-1* mutants show higher sensitivity to GABA receptor antagonist PTZ than the WT animals. Representative still frame images demonstrating *casy-1* mutant *C*. *elegans* with anterior convulsions. The still frame images are representative frames from movies (7 frames/second), which are available in the supporting information. Scale bar, 100μm. The graph shows the fraction of animals showing anterior ‘head bobs’ after 30 minute and 60-minute exposure to 10 mg/ml PTZ. The sensitivity to PTZ could be fully rescued by expressing all *casy-1* isoforms in GABAergic neurons but not in cholinergic neurons or muscle. Assays were done (~10 *C*. *elegans*/assay) at least thrice. Data are represented as mean ± S.E.M. Values that differ significantly from WT animals are indicated (*p<0.01, ***p<0.0001 using one-way ANOVA and Bonferroni's Multiple Comparison Test). Representative fluorescent images of (D) GABAergic [*nuIs376* (P*unc-25*::SNB-1::GFP)] or (E) cholinergic [*nuIs152* (P*unc-129*:: SNB-1::GFP)] synapses in the dorsal cord of WT or *casy-1* mutant. Scale bar, 8μm. Cholinergic synapses are largely normal in *casy-1* mutants, while GABAergic synapses showed a subtle but significant decrease in fluorescent intensity when compared to WT animals. Quantification of fluorescent intensity is normalized to WT values. The number of animals analyzed for each genotype is indicated at the base of the bar graph. Quantified data are displayed as mean ± S.E.M. and were analyzed by two-tailed Student’s *t*-test, “ns” indicates not significant in all Figures.

To identify specific neurons where CASY-1 could be functioning to regulate synaptic transmission, all isoforms of CASY-1 were expressed under cholinergic (*unc-17*) or GABAergic (*unc-25*) neuron-specific promoters. All three isoforms completely rescued the Aldicarb hypersensitivity of *casy-1* mutants when expressed in GABAergic neurons but not in cholinergic neurons (Fig 3A). These results indicate that CASY-1 could be regulating synaptic transmission in GABA motor neurons at the NMJ. Although expression of CASY-1 isoforms was not observed in the body-wall muscle, however, to remove the possibility of any retrograde signaling from the body-wall muscle effecting neuronal behavior at the NMJ, a transgenic line expressing CASY-1A under a body-wall muscle specific promoter (*myo-3*) was generated. Expression of CASY-1A in body-wall muscles failed to rescue the Aldicarb hypersensitivity of *casy-1* mutants (Fig 3A).

Previous experiments (Fig 2C) show that the C-terminal of CASY-1 is sufficient to rescue the Aldicarb hypersensitivity of *casy-1* mutants. To further strengthen our hypothesis, a transgenic line CASY-1A (ΔC) was generated, in which the entire C- terminal (880–984 aa) was removed from CASY-1A and expressed under the GABA-specific promoter (*unc-25*). This line failed to rescue the Aldicarb defects of *casy-1* mutants (Fig 3B), further signifying the importance of the C-terminal of CASY-1 at the NMJ.

To further investigate the role of GABA signaling in *casy-1* mutants, an assay using the drug Pentylenetetrazole (PTZ), a potent antagonist of GABA_A_ receptors [45], was performed. PTZ has been shown to be effective in *C*. *elegans* for generating a convulsive phenotype called ‘head bobs’, which is used as an indicator of reduced GABAergic synaptic-transmission [46]. Mutants in *unc-25*, a GABA-synthesizing enzyme, were used as positive controls (S3A Fig). The *casy-1* mutant animals showed a strong convulsion phenotype after a 30-minute exposure to 10mg/ml PTZ and by the end of 60 minutes, a significant fraction of *casy-1* mutants showed convulsions (Fig 3C). Additionally, *casy-1* mutants also showed a tail shrinking phenotype, a characteristic of GABA mutants in *C*. *elegans* [47] (S1–S3 Movies). The convulsive phenotype of *casy-1* mutants was completely rescued by expressing the CASY-1 isoforms in GABAergic neurons but not in cholinergic neurons or muscle (Fig 3C) further implicating the role of CASY-1 in GABA signaling.

Reduced GABAergic synaptic transmission could be either due to reduced release of GABA from the GABAergic motor neurons (pre-synaptic) or reduced response of muscle to GABA due to lower expression of GABA_A_ receptors on the muscle (post-synaptic). To differentiate between these two possibilities, pre-synaptic and post-synaptic markers were analyzed in the *casy-1* mutant background. Initially the expression of pre-synaptic markers under cholinergic and GABAergic neuron specific promoters were investigated. First, GFP–tagged SNB-1, a *C*. *elegans* ortholog of the mammalian Synaptic Vesicle (SV) protein synaptobrevin was analyzed. Synaptobrevin localizes in a punctate pattern along the dorsal cord of *C*. *elegans* [40]. Mutants in *casy-1* showed a subtle but significant decrease in GABAergic::SNB-1::GFP levels, suggesting fewer GABA vesicles at the synapse (Fig 3D). Analysis of the Dorsal Nerve Cord (DNC) puncta to VNC cell body fluorescence ratio further displayed a significant decrease supporting the presence of fewer GABA vesicles at the DNC synapses (S3B Fig). Fluorescence intensity of cholinergic synapses was indistinguishable in *casy-1* mutants from wild-type (WT) animals, suggesting normal cholinergic signaling (Fig 3E). The synapse density in the dorsal cord was also examined, by quantifying SYD-2 puncta, an ortholog of the mammalian active zone protein α-Liprin [40]. The density of cholinergic and GABAergic synapses were largely normal, indicating that synapse development is largely unaffected in *casy-1* mutants (S3C and S3D Fig).

The *C*. *elegans* body muscles express two classes of Acetylcholine receptors (AChRs); nicotine-sensitive (nAChR) and levamisole-sensitive (LAChR) receptors as well as a single class of GABA_A_ receptors. There was no significant change in the fluorescent intensity of the GFP- tagged cholinergic receptors (S3E Fig) in the mutants. The GABA_A_ receptor, UNC-49 also showed similar levels of expression and localization in *casy-1* mutants when compared to WT animals (S3F Fig). To further ensure that post-synaptic GABA_A_ receptors functioned normally in the mutants, a GABA agonist Muscimol was utilized. *C*. *elegans* placed on Muscimol show various degrees of responses depending on their sensitivity to the drug. The most characteristic phenotype is the “rubber band phenotype”, wherein the animals contract and relax without displacement following prodding on the head [48]. If reduced GABAergic signaling in *casy-1* mutants is due to lower release of GABA from the pre-synaptic terminal, no difference in the severity of the rubber band phenotype would be observed following incubation with Muscimol. When *casy-1* mutants were tested in the Muscimol assay they behaved like WT *C*. *elegans* (S3G Fig), again strengthening our hypothesis that CASY-1 functions pre-synaptically at the NMJ. To further ensure that cholinergic transmission is normal in *casy-1* mutants, we performed a Levamisole assay. Levamisole is an agonist for L-type cholinergic receptors and exposure to Levamisole results in a time-course induced paralysis in WT animals. Mutants in *casy-1* showed a response similar to WT animals in the Levamisole assay suggesting normal LAChR signaling at the NMJ (S3H Fig).

### Mutants in *casy-1* have reduced GABAergic neurotransmission at the NMJ

**Fig 4:**
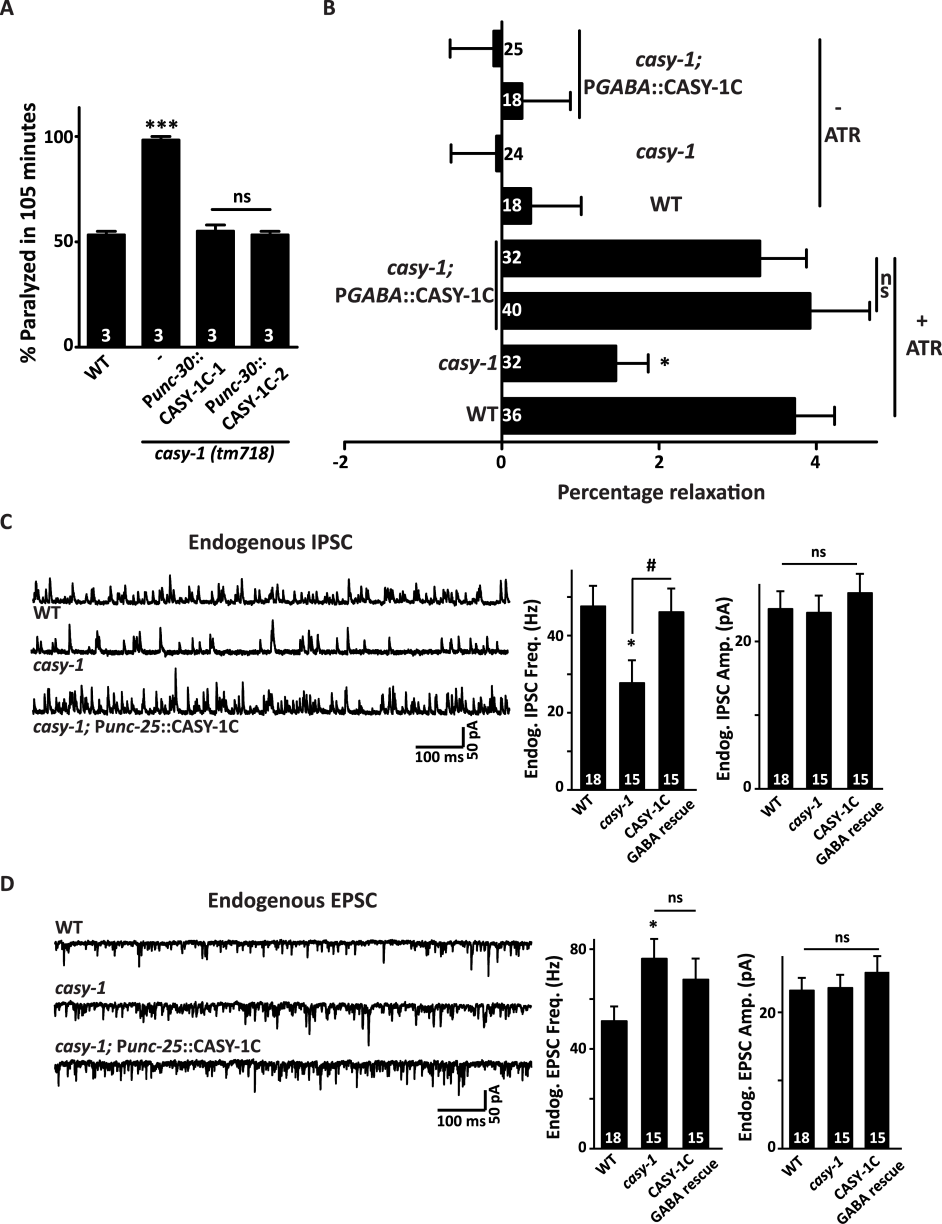
*casy-1* mutants show decreased GABAergic synaptic transmission at the NMJ. (A) Expression of the CASY-1C specifically in DD and VD neuronal subtypes using GABAergic motor neuron specific promoter (P*unc-30*) establish that CASY-1 is explicitly functioning in GABAergic motor neurons to regulate synaptic transmission. Assays were done 3 times as indicated in Figures (~20 *C*. *elegans*/ assay). Data are represented as mean ± S.E.M. (***P<0.0001 using one-way ANOVA and Bonferroni's Multiple Comparison Test). “ns” indicates not significant in all Figures. (B) Optogenetic stimulation of GABAergic neurons using Channelrhodopsin (ChR2) [*zxIs3* (*unc-47*p::ChR2(H134R)::YFP)] showed that the *casy-1* mutant animals relax significantly less than WT *C*. *elegans* upon blue light exposure (percent change in body length before and after optogenetic stimulation). Expressing the CASY-1C isoform under a GABAergic neuron specific promoter (P*unc-25*) completely rescues the relaxation defect in *casy-1* mutant animals. Data is shown for two independent rescue lines. The graph shows the percentage change in body length for both +ATR and–ATR controls. The numbers of animals analyzed for each genotype are indicated. Data are represented as mean ± S.E.M. (*p<0.01 using one-way ANOVA and Bonferroni's Multiple Comparison Test). “ns” indicates not significant in all Figures. (C) mIPSCs were recorded from body wall muscles of adult animals for the indicated genotypes. Representative traces of mIPSCs and summary data for frequency and amplitude are shown. The *casy-1* mutants showed a significant decrease in mIPSCs rate compared to WT *C*. *elegans*, suggesting a decreased GABAergic neurotransmission at the NMJ. The mIPSC amplitude, however, remains unaltered suggesting normal muscle responsiveness in the mutant. The decreased mIPSCs rate of *casy-1* mutants can be significantly rescued by expressing CASY-1C specifically in GABA motor neurons (P*unc-25*). (D) Depicts traces and quantified data for the mEPSCs recorded from the *C*. *elegans* body-wall muscles. There is a subtle but significant increase in mEPSC frequency in *casy-1* mutants, and this defect was not rescued by expressing CASY-1C in GABA motor neurons. For both C and D, the number of animals analyzed for each genotype is indicated. Data are represented as mean ± S.E.M. *p<0.05 with respect to WT and # *p*<0.05 with respect to *casy-1* mutants, using the one-way ANOVA with Dunnett’s post test).

The *C*. *elegans* nervous system consists of 26 GABAergic neurons which include six DD and thirteen VD neurons that innervate the dorsal and ventral body-wall muscles respectively, four RME motor neurons that innervate head muscles, the AVL and DVB neurons that are pre-synaptic to the enteric muscles and RIS which is an interneuron [47, 49]. To confirm that CASY-1 functions specifically in GABA motor neurons (D- type neurons), a transgenic line expressing the CASY-1C isoform specifically in DD and VD class of GABA motor neurons using the *unc-30* promoter that also expresses in some non-GABAergic neurons was generated [50]. Expression of CASY-1C in just D-type motor neurons completely rescued the Aldicarb hypersensitivity of *casy-1* mutants, indicating that CASY-1 functions in motor neurons to regulate synaptic transmission at the NMJ (Fig 4A).

To further explore the function of *casy-1* in GABA neurotransmission, we utilized an optogenetic tool, where channelrhodopsin (ChR2), a light-gated cation channel that allows non-specific flow of cations into the cell leading to electrical excitation, was used [51]. A transgenic line that expressed ChR2 specifically in GABAergic neurons was utilized. The light-based activation of GABA motor neurons leads to a measurable change in body length (relaxation). Activation of GABAergic ChR2 results in a reduced relaxation in *casy-1* mutants in comparison to WT controls. These results suggest a lower release of GABA from the pre-synaptic termini in *casy-1* mutants, which in turn results in less relaxation upon optogenetic stimulation. Expressing the CASY-1C isoform in GABAergic neurons completely rescued the decreased relaxation defect of *casy-1* mutants (Fig 4B).

To evaluate changes in endogenous synaptic transmission, whole-cell patch clamp recordings of the muscles under voltage-clamp conditions from dissected *C*. *elegans* were analyzed. Endogenous excitatory and inhibitory post-synaptic currents (EPSCs and IPSCs) that indicate the frequency and amplitude of neurotransmitter release from cholinergic and GABAergic motor neurons onto the body-wall muscle were measured in the WT and *casy-1* mutant animals. The *casy-1* mutants had a significantly lower endogenous IPSC rate compared to WT animals (Fig 4C). The amplitude of IPSCs was unaltered. Collectively, these results indicate that the release of GABA is reduced in *casy-1* mutants while the muscle responsiveness to GABA remained unaffected. Expression of CASY-1C specifically in GABA motor neurons significantly rescued the decreased IPSC frequency in the *casy-1* mutants (Fig 4C). To validate the specific role of shorter *casy-1* isoforms in regulating GABA signaling, a transgenic line that expresses CASY-1A isoform under its endogenous promoter was tested to rescue the decreased IPSC frequency in the *casy-1* mutants. This transgenic line did not show significant differences in IPSC rate when compared to the *casy-1* mutant *C*. *elegans*. This experiment further strengthens our data that the shorter isoforms expressed in motor neurons function to modulate GABAergic transmission (S4 Fig).

Interestingly, we found that the endogenous EPSC rate showed a significant increase in *casy-1* mutants when compared to WT animals (Fig 4D). Expression of CASY-1C specifically in GABA motor neurons could not rescue the EPSC frequency in the *casy-1* mutants (Fig 4D). The amplitude of EPSCs was again unaffected. Changes in acetylcholine release from cholinergic motor neurons are likely to be secondary, as functional rescue experiments showed that expressing *casy-1* in GABAergic, but not in cholinergic motor neurons, rescue the Aldicarb hypersensitivity of *casy-1* mutants. Thus, it is possible that the increased EPSC frequency in *casy-1* mutants could be due to the function of CASY-1 in higher levels of neuronal circuits (e.g. sensory or interneurons).

### The *casy-1* isoforms express in different neuronal subtypes and are targeted to the pre-synaptic termini

**Fig 5:**
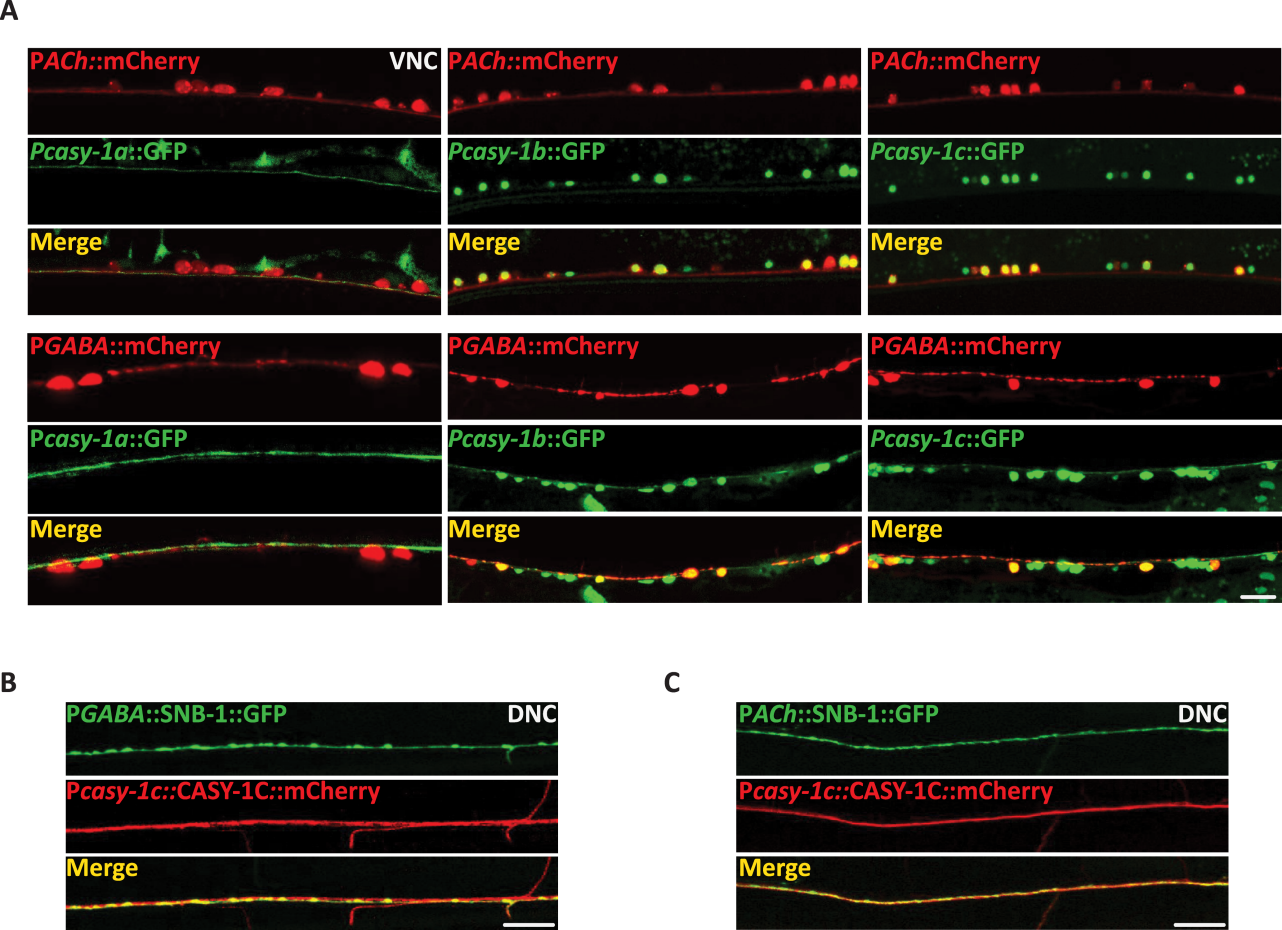
CASY-1 isoforms shows differential spatial localization. (A) Expression of GFP under isoform-specific *casy-1* promoters. *casy-1a* transcriptional reporter does not co-localize with mCherry marked cholinergic or GABAergic motor neurons. *casy-1b* and *casy-1c* expression reporters show expression in both cholinergic and GABAergic motor neurons. Anterior is to the left in all panels. Scale bar, 8μm. (B) Representative fluorescent images of P*casy-1c*::CASY-1C::mCherry translational reporter showing co-localization with the GABAergic *nuIs376* [P*unc-25*::SNB-1::GFP] pre-synaptic markers suggesting the presence of CASY-1C in the GABAergic NMJ pre-synaptic termini. Scale bar, 10μm. (C) Representative fluorescent images of P*casy-1c*::CASY-1C::mCherry translational reporter showing co-localization with the cholinergic *nuIs152* [P*unc-2129*::SNB-1::GFP] pre-synaptic markers suggesting the presence of CASY-1C in the cholinergic synapses. Scale bar, 10μm.

Expression of all CASY-1 isoforms in GABAergic neurons completely rescued the Aldicarb hypersensitivity of *casy-1* mutants (Fig 3A). Also, *casy-1b* and *casy-1c* transcriptional reporters express in motor neurons (Fig 1E). To determine the specific class of motor neurons where *casy-1* isoforms are expressing, isoform specific- GFP transcriptional reporters were co-expressed with cholinergic or GABA specific mCherry-tagged reporter lines. The *casy-1a* transcriptional reporter showed no co-localization with either cholinergic or GABAergic motor neurons (Fig 5A, column one). However, *casy-1b* and *casy-1c* showed co-localization with both cholinergic and GABAergic neuronal markers (Fig 5A, columns two and three). This data suggests that the shorter isoforms of *casy-1* are probably functioning in GABA motor neurons to regulate GABA release and hence affect synaptic transmission at the NMJ, however, the role of these isoforms in cholinergic neurons is still unclear.

To examine specific subcellular localization of the shorter *casy-1* isoforms, CASY-1C::mCherry was expressed under its own promoter. The CASY-1C::mCherry protein localizes all along the dorsal cord axons and shows co-localization with the GABAergic and cholinergic pre-synaptic SNB-1::GFP markers (Fig 5B and 5C). This suggests the presence of CASY-1 at the synapse. The expression of CASY-1C::mCherry at the DNC synapses was diffuse rather than punctate. This could be due to the lateral diffusion followed by ecto-domain shedding at the synaptic cleft (described below). Expression of mCherry and GFP- tagged CASY-1C fully rescued the Aldicarb sensitivity of *casy-1* mutants suggesting that the tagged proteins are functional (S5A Fig).

Mammalian calsyntenins have been shown to be cleaved at their extracellular region [19, 23]. Further, the CASY-1A isoform has previously been shown to be cleaved in its extracellular region juxtaposed to the membrane by synaptic cleft peptidases, resulting in the release of the entire N- terminal into the synaptic cleft [21] (illustrated in S5B Fig). To figure out if CASY-1B and CASY-1C are also cleaved once they reach the synapse, N- terminal mCherry fusion transgenes of CASY-1B and CASY-1C were generated. The mCherry tagged N-terminal of the shorter CASY-1 isoforms was detected in the coelomocytes (S5B Fig; bottom panels), which are macrophage- like scavenger cells that take up any waste or secreted proteins from the body cavity (reviewed in[52]). These results allow us to conclude that all CASY-1 isoforms are cleaved at their N-terminal region by synaptic cleft peptidases, resulting in the release of the ectodomain into the body cavity. These results also provide appropriate justification to how CASY-1A isoform, which does not express in motor neurons could rescue the Aldicarb hypersensitivity when expressed in GABA motor neurons. CASY-1 isoforms are present on the SV precursors with their N-terminal facing the SV lumen, while the conserved C-terminal faces towards the cytosol where it can interact with a wide variety of cytoplasmic proteins. Since the entire C-terminal is totally conserved in the three *casy-1* isoforms, expression of any of the three isoforms in GABA motor neurons could potentially rescue the Aldicarb phenotype of the *casy-1* mutants.

Although this data could explain the rescue of Aldicarb hypersensitivity by the CASY-1A isoform in GABA motor neurons, the question still remains as to how the expression of CASY-1A under its endogenous promoter could rescue the Aldicarb hypersensitivity in *casy-1* mutants (Fig 1C). CASY-1A expression is highly enriched in the head sensory neurons. Previous reports have highlighted the role of genes that function in higher levels of locomotion circuit to regulate motor circuit activity at the NMJ [53, 54]. Future investigations examining CASY-1A functions in sensory neurons might shed some light on this aspect.

### Distribution of the SV protein SNB-1 is altered at GABAergic synapses in *casy-1* mutants

**Fig 6:**
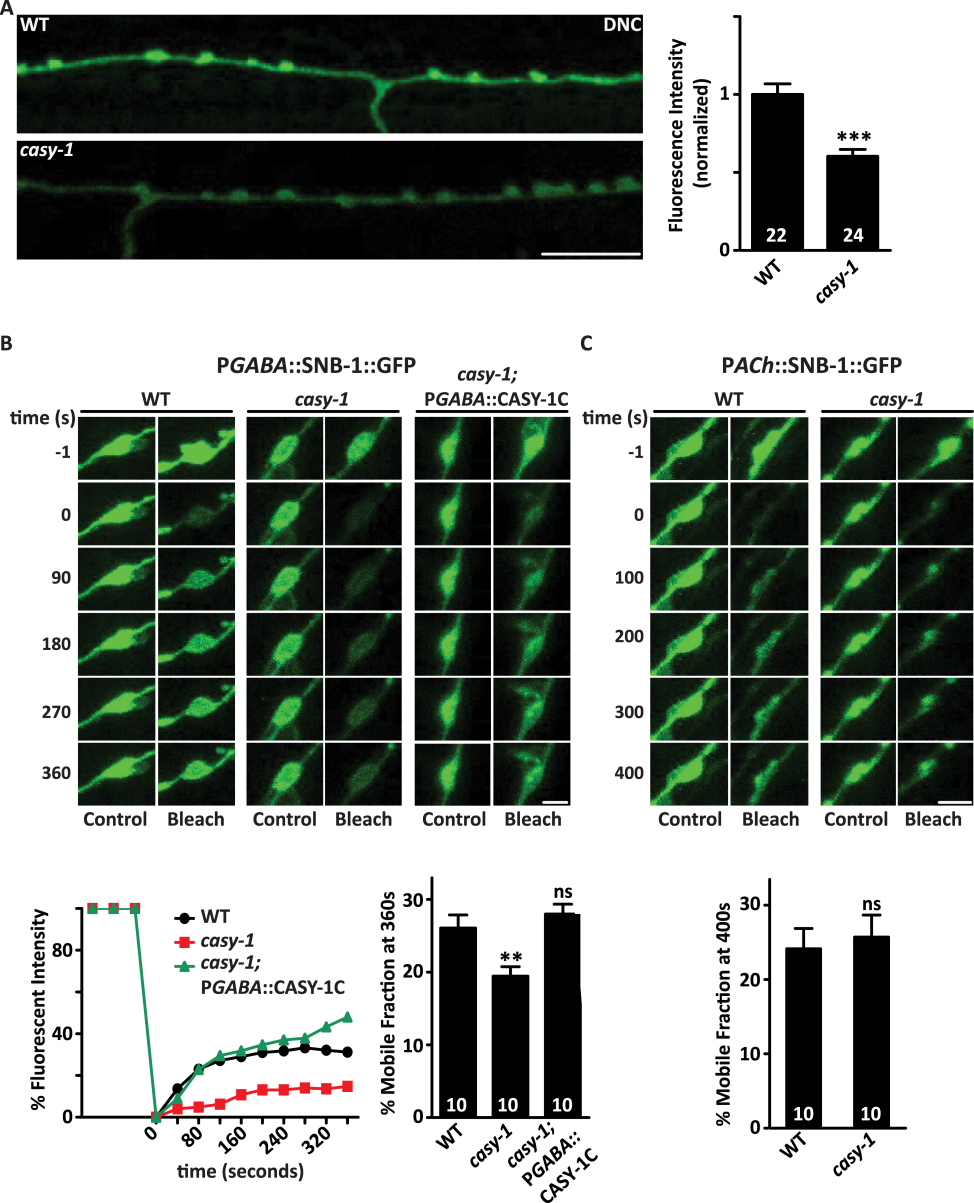
*casy-1* isoforms are required for normal GABA release at NMJ. (A) GABA SV release is compromised in *casy-1* mutants. Images show the DNC of adult hermaphrodites expressing pH-sensitive GFP reporter (*superecliptic pHluorin*) tagged to the luminal domain of synaptobrevin. pHluorin fluorescent intensity is significantly reduced in the *casy-1* mutants suggesting fewer GABA vesicles functional at the synapse. Quantification of fluorescent intensity is normalized to WT values. The number of animals analyzed for each genotype is indicated at the base of the bar graph. Quantified data are displayed as mean ± S.E.M. and were analyzed by two-tailed Student’s *t*-test. Scale bar, 8μm. (B) FRAP analysis of SNB-1::GFP levels in GABAergic motor neurons reveals that the dynamics of SV mobility is reduced in *casy-1* mutants. Representative confocal images of pGABAergic::SNB-1::GFP levels compared between WT, *casy-1* and *casy-1*; pGABAergic::CASY-1C rescue shows images before photo-bleaching *(pre-bleach)*, immediately after photo-bleaching *(post-bleach)* and 360 sec after photo-bleaching *(recovery)*. Scale bar, 2μm. At time 0, a single puncta of SNB-1::GFP was photo-bleached. Recovery of SNB-1::GFP levels were subsequently monitored at the photo-bleached and a neighboring control puncta. The fractional recovery of fluorescence 360 sec after photo-bleaching is shown. Recovery was measured with the pre-bleach fluorescence intensity being 100% and the post-bleach intensity at time 0 being 0%. The fluorescence intensity of control unbleached puncta did not change significantly during the period of recovery. (C) FRAP analysis of SNB-1::GFP levels in cholinergic motor neurons illustrated that mobility dynamics of SNB-1::GFP is normal in cholinergic motor neurons in *casy-1* mutant animals. Representative confocal images of pCholinergic::SNB-1::GFP levels images prior to photo-bleaching *(pre-bleach)*, immediately after photo-bleaching *(post-bleach)* and 400 sec after photo-bleaching *(recovery)* Scale bar, 2μm. The fractional recovery of fluorescence 400 sec after photo-bleaching is shown. The number of animals analyzed are indicated for each genotype. Data are represented as mean ± S.E.M. (**p<0.001 using one-way ANOVA and Bonferroni's Multiple Comparison Test).

Reduced SNB-1::GFP levels, less relaxation upon optogenetic stimulation of GABAergic neurons and decreased endogenous IPSC rate (Figs 3D, 4B and 4C) all highlight the role of CASY-1 in regulating GABA release from motor neurons. To further address this role of *casy-1*, a transgenic line in which the luminal domain of synaptobrevin is tagged with superecliptic pHluorin; a GFP reporter expressed specifically in GABAergic neurons [55] was utilized. pHluorin is highly pH-sensitive and its fluorescence remains quenched in the acidic environment of the SV lumen, however, there is a dramatic increase in the fluorescence as soon as the vesicle fuses onto the membrane relieving the tag from the acidic environment of the SV [56, 57]. To examine if *casy-1* mutants have fewer GABA vesicles at the synapse, and hence less release of GABA, we monitored the fluorescence intensity of pHluorin at the dorsal cord synapses. pHluorin intensity was significantly reduced in the dorsal cord synapses of *casy-1* mutants further confirming the role of CASY-1 in the release of GABA at NMJ (Fig 6A).

Further, we addressed the possibility of the involvement of CASY-1 in GABA vesicle trafficking by performing fluorescence recovery after photobleaching (FRAP) analysis of SNB-1::GFP levels. The recovery rate of SNB-1::GFP depends upon two factors; transport of new SV precursors at the synapse by motor-mediated trafficking or diffusion from neighboring synapses into the bleached area. The recovery rate of GABAergic SNB-1::GFP was significantly reduced in *casy-1* mutants suggesting that the mobility dynamics of SVs at the NMJ is compromised in the mutants. The recovery rate was completely restored by expressing the CASY-1C isoform specifically in GABAergic neurons (Fig 6C). We also monitored the mobility dynamics in cholinergic motor neurons but found no significant change in *casy-1* mutants compared to WT animals (Figs 6C and S6), suggesting that CASY-1 is specifically functioning to regulate SV release in GABA motor neurons.

### CASY-1 regulates the transport of GABAergic vesicle synaptic precursors along the commissures

**Fig 7:**
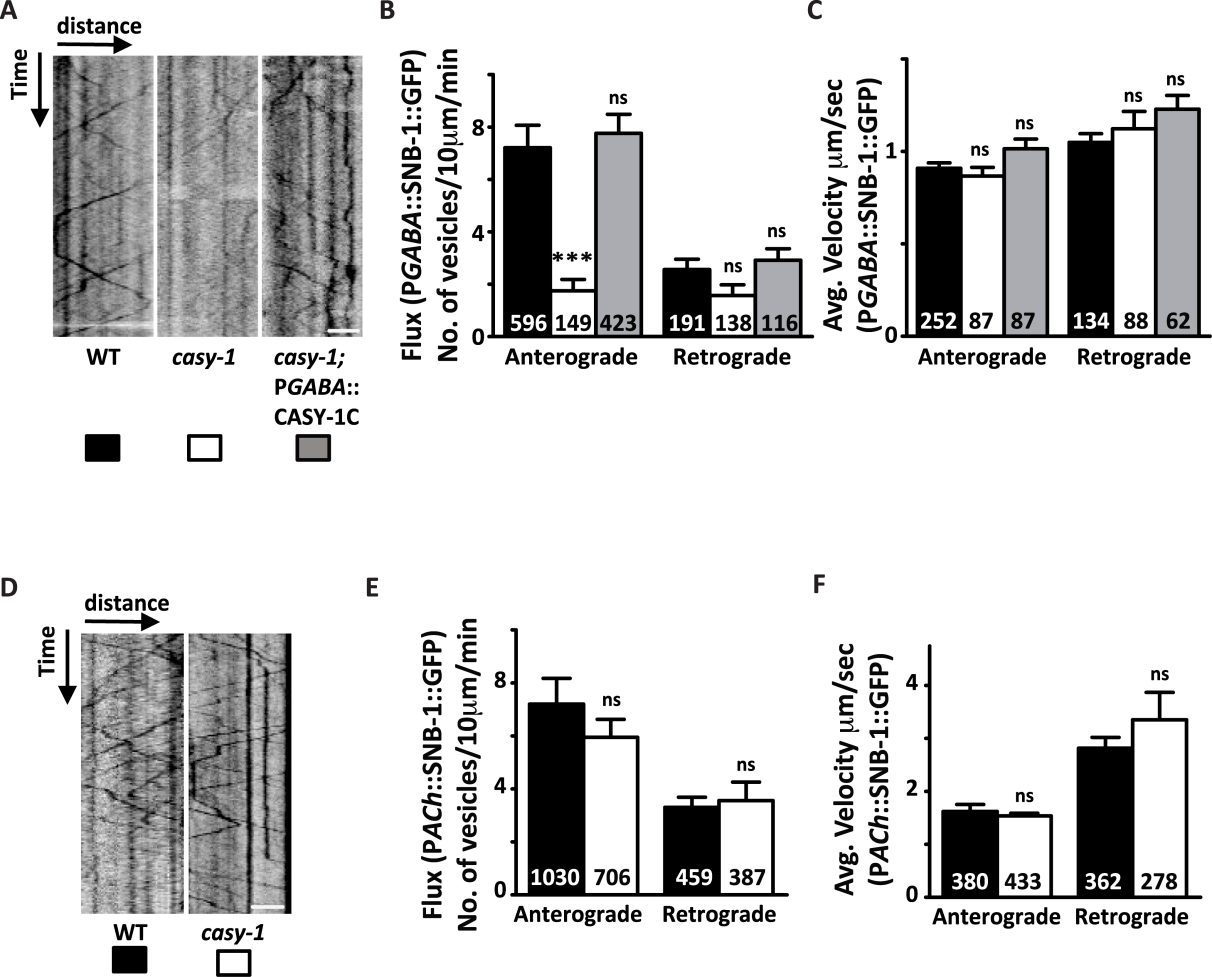
CASY-1 is required for transport of SV precursors in GABAergic motor neurons. (A) Representative GABAergic::SNB-1::GFP transport kymographs in WT, *casy-1* and *casy-1*; pGABAergic::CASY-1C rescue. For all kymographs, ventral cell body is to the right. Anterograde movement is from right to left. All kymographs are clipped to 10 μm (distance) x 1 min (time) (77x180 pixels). Scale bar, 3 μm. (B-C) Quantification of anterograde and retrograde SV flux in young adult animals. (B) Comparison of mean anterograde and retrograde flux (normalized to a distance of 10 μm and a time of 1 min) between WT, *casy-1* mutant and *casy-1*; pGABAergic::CASY-1C rescue animals. (C) Comparison of mean anterograde and retrograde velocities between WT, *casy-1* mutant and *casy-1*; pGABAergic::CASY-1C rescue animals are shown. *casy-1* mutants show significantly reduced anterograde vesicular flux compared to WT animals. Reduced GABAergic anterograde flux was completely rescued by expressing CASY-1C specifically in GABAergic motor neurons. (D) Representative Cholinergic::SNB-1::GFP trafficking kymographs in WT and *casy-1* mutant animals. For all kymographs, ventral cell body is to the right. All kymographs are clipped to 10 μm (distance) x 1 min (time). Scale bar, 3 μm. (E-F) Quantification of anterograde and retrograde SV transport in young adult animals (E) Average flux and (F) Average velocity are shown. Flux is defined as the number of moving particles per 10 μm per min. n represents number of particles analyzed for the analysis. Data are represented as mean ± S.E.M. (***p<0.0001 using two-way ANOVA and Bonferroni's Multiple Comparison Test, “ns” indicates not significant in all Figures).

In *C. elegans*, the motor neuron soma that are present along the ventral nerve cord send out axons in the anterior direction, which extend commissures dorsally by crossing the midline and fasciculate with the DNC to form synapses with the body wall muscles. To address how *casy-1b/casy-1c* regulate the trafficking of SV precursors from motor neuron cell bodies to the DNC synapses, the transport characteristics of SNB-1::GFP tagged vesicles along the commissures were examined using time lapse imaging. We first assayed SV transport in D- type GABAergic motor neurons. In WT animals, SNB-1::GFP labeled vesicles are transported in both anterograde and retrograde directions, with an anterograde bias (Fig 7B). However, in *casy-1* mutants, the SV anterograde transport was significantly reduced, although, the velocity of SNB-1::GFP remains similar to WT animals. Decrease in anterograde vesicular flux was rescued by expressing the CASY-1C isoform in GABAergic motor neurons (Fig 7A–7C and S4–S6 Movies). We next analyzed the transport characteristics of SNB-1::GFP vesicles in Cholinergic motor neurons. Vesicular velocity for SNB-1::GFP was higher in Cholinergic neurons compared to GABAergic neurons. Analysis of SNB-1::GFP transport in cholinergic motor neurons of *casy-1* mutants showed that anterograde and retrograde vesicular flux and velocity are similar to that of wild type, suggesting that vesicular transport is not affected in cholinergic neurons in *casy-1* mutants. (Fig 7D–7F and S7 and S8 Movies). This data suggests that the shorter CASY-1 isoforms function specifically in GABAergic motor neurons to modulate the release kinetics of GABA by directly influencing the transport of SV precursors.

The expression of different vesicular cargo proteins in the *casy-1* mutants was next examined in order to investigate if *casy-1* mutants have general trafficking defects. The reduced SNB-1::GFP levels in the GABA motor neurons (Fig 3D) were due to decreased transport of GABA SV precursors at the synapse and hence suggests that monitoring the fluorescent intensity of tagged cargoes might provide an indication of possible trafficking defects in the *casy-1* mutants. The transport characteristics of cholinergic SNB-1::GFP were unaltered in the *casy-1* mutants which clearly suggests that defects in vesicular cargo trafficking is not a general phenomenon in *casy-1* mutants. To validate this further, the fluorescence intensity of diverse vesicular cargo was monitored at the GABAergic DNC synapses in *casy-1* mutants. The fluorescence intensity of tagged- mitochondrial cargo (MITO::GFP) as well as early endosomes (RAB-5::YFP) was not affected in the GABAergic neurons of *casy-1* mutants (S7A and S7B Fig). However, the fluorescence intensity of a lysosomal marker (CTNS-1::GFP) was significantly reduced suggesting the presence of fewer lysosomes at the synapse (S7C Fig). This decrease in lysosomal marker fluorescence could be due to absence of the interaction of CASY-1 with the kinesin light chain motor proteins like *klc-2* that forms functional complexes with the kinesin heavy chain motor proteins *unc-116* required for trafficking of lysosomal cargos [58, 59].

### CASY-1 is a potential cargo adaptor for UNC-104 mediated transport of SV precursors

Mutants in *casy-1* have fewer GABA vesicles at the synapse as demonstrated by a significant decrease in SNB-1::GFP levels at the NMJ (Fig 3D). They also show reduced recovery rates of SNB-1::GFP following FRAP analysis, suggesting the possibility that fewer SV precursors are trafficked to the NMJ (Fig 6B). UNC-104/KIF1A is a kinesin-3 motor that has a conserved role in trafficking SV precursors from the cell body to the synapse [60–63]. To address the possibility that CASY-1 could be interacting with UNC-104 to regulate the anterograde trafficking of SVs in GABAergic motor neurons, Aldicarb assays were performed after silencing the *casy-1* gene in the *unc-104* mutant background using RNAi. The reverse experiment of knocking down *unc-104* in the *casy-1* mutant background was also performed. We performed RNAi knockdown experiments as we were unable to make *unc-104; casy-1* double mutants due to close proximity of the genes on Chromosome II. Both the knock-down and knock-out of *unc-104*, resulted in resistance to Aldicarb as has been previously reported [64]. Further, RNAi knock-down of *unc-104* in the *casy-1* mutant background, completely abolished the hypersensitivity of *casy-1* mutants and resulted in resistance to Aldicarb just like what was seen in *unc-104* mutants, suggesting that UNC-104 may genetically interact with CASY-1 (S8A Fig). However, resistance to Aldicarb could also be due to the dominant phenotype of *unc-104* over *casy-1* gene function.

**Fig 8:**
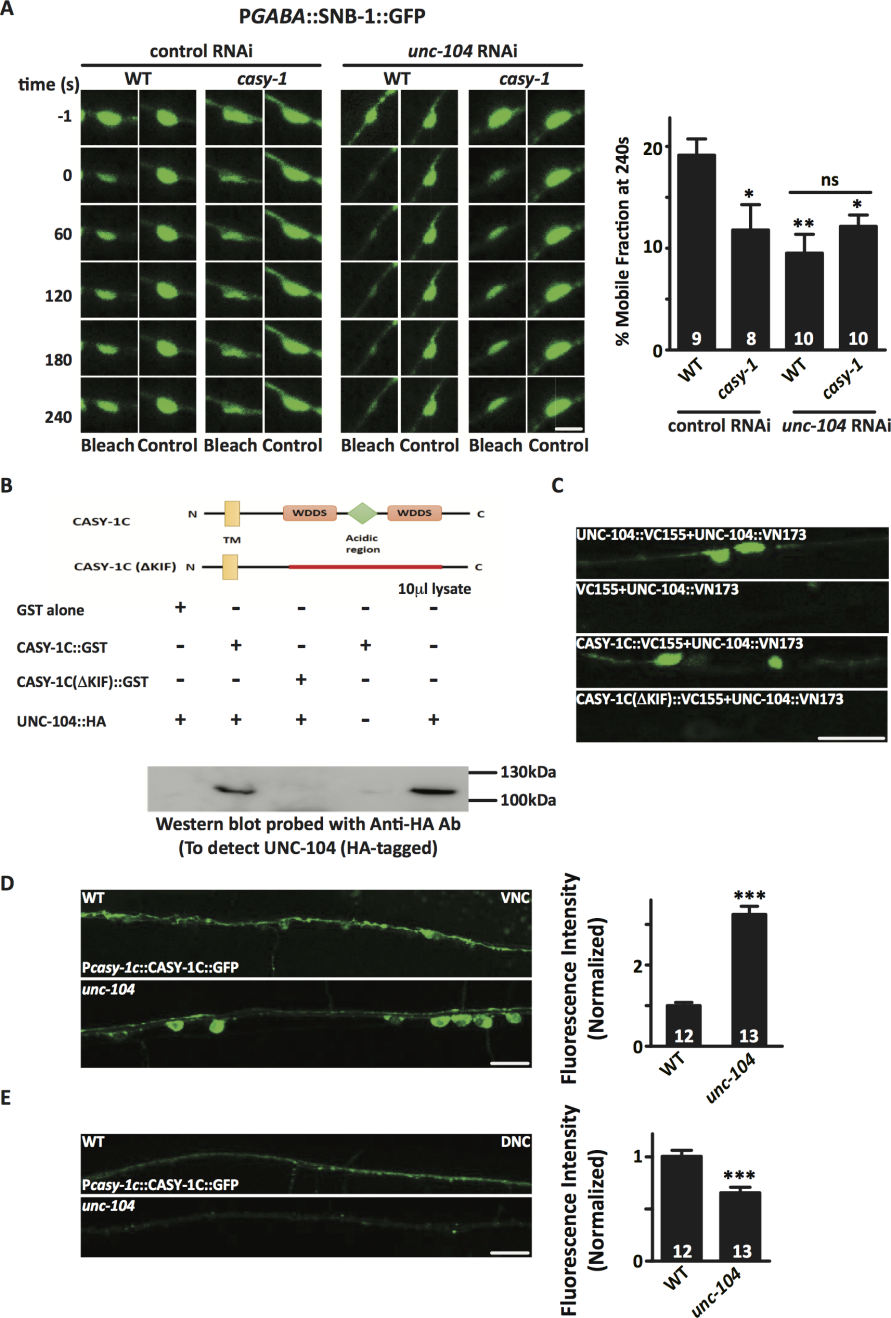
CASY-1 interacts with the kinesin motor UNC-104/KIF1A to regulate the trafficking of GABA vesicles. (A) FRAP analysis of SNB-1::GFP levels in GABAergic motor neurons reveals that the dynamics of SV mobility is reduced after *unc-104* RNAi. Representative confocal images of pGABAergic::SNB-1::GFP levels compared between WT, *casy-1* mutant, *unc-104* RNAi and *casy-1*; *unc-104* RNAi shows images before photo-bleaching *(pre-bleach)*, immediately after photo-bleaching *(post-bleach)* and 240 sec after photo-bleaching *(recovery)*. Scale bar, 2μm. The fractional recovery of fluorescence 240 sec after photo-bleaching is shown. (B) The upper panel shows the domain organization of CASY-1C illustrating the two conserved WDDS motifs surrounding an acidic region containing stretches of glutamic acid residues. The CASY-1C (ΔKIF) represents a deletion in the region harboring the two WDDS motifs and the acidic region (amino acids 70–148) in the C- terminal region of CASY-1C. The lower panel shows a GST pull down assay showing that CASY-1 C-terminal can interact with the tail region of UNC-104. HA-tagged UNC-104 expressed in bacterial cell extract was incubated with bead-bound GST fusion proteins CASY-1C GST and CASY-1C (ΔKIF) GST. The blot was probed with anti-HA antibody. Significant pull down of 110 KDa HA-tagged UNC-104 was observed with CASY-1C GST. A deletion in the KIF-binding domain in CASY-1C eliminates the pull down of HA-tagged UNC-104 from the bacterial lysate. 10 μl of bacterial cell lysate was loaded as control. (C) Representative fluorescence micrographs summarizing the results of BiFC assay. BiFC signals from UNC-104 VN and UNC-104 VC interactions along the VNC (positive control). No BiFC signals were observed from UNC-104 VN and Empty VC (negative control). BiFC signals of UNC-104 VN and CASY-1C VC interactions observed in the GABA ventral cord motor neurons. Intensity of BiFC signals were significantly reduced in UNC-104 VN and CASY-1C (ΔKIF) VC BiFC pair. Scale bar, 10μm. (D) Intracellular localization of P*casy-1c*::CASY-1C::GFP in VNC cell bodies in *unc-104* mutant background. In this mutant background, CASY-1C::GFP is significantly sequestered in cell bodies compared to WT control animals. A circular ROI was quantified in each image for two cell bodies and then averaged for each image intensity values. (E) In parallel, the fluorescence intensity of CASY-1C::GFP was significantly decreased at the DNC synapses in *unc-104* mutants. Quantification of fluorescent intensity is normalized to WT values. The number of animals analyzed for each genotype is indicated at the base of the bar graph. Quantified data are displayed as mean ± S.E.M. and were analyzed by two-tailed Student’s *t*-test. Scale bar, 10μm.

To further validate the genetic interaction between *casy-1* and *unc-104*, FRAP analysis of SNB-1::GFP in GABAergic motor neurons after RNAi knockdown of *unc-104* in a *casy-1* mutant background was performed. The recovery rate of SNB-1::GFP was significantly reduced after knockdown of *unc-104*. However, no significant difference between the recovery rates was observed after *unc-104* knockdown in WT and *casy-1* mutant background, further supporting a genetic interaction between *casy-1* and *unc-104* (Fig 8A).

The *C*. *elegans* CASY-1 has previously been shown to physically interact with the kinesin light chain-2 (KLC-2) in yeast two-hybrid assays [59]. These studies suggest CASY-1 acts as a broad regulator for transport of multiple neuronal cargoes. To investigate the role of CASY-1 in general transport mechanisms, a possible physical interaction between CASY-1 and UNC-104 in a yeast two-hybrid assay was examined. The interaction of the CASY-1 C- terminal with several UNC-104 domain constructs was observed. Our data suggests that a weak interaction occurs between the cytoplasmic tail of CASY-1 and the C-terminal of UNC-104 that largely includes the stalk region and the Pleckstrin Homology (PH) domain, a domain responsible for cargo vesicle binding to UNC-104 [65]. Subtle interactions were also observed with the UNC-104 motor domain and FHA domain. This data suggests that the C-terminal of CASY-1 could directly interact with UNC-104. (S8B Fig). However, since the interaction between UNC-104 and CASY-1 appeared weak in the yeast-two-hybrid assay, we could not rule out the possibility that CASY-1 interacts indirectly with UNC-104, through other adaptor molecule/s.

After identifying the C-terminal PH domains and adjacent regions as critical mediators of CASY-1 and UNC-104 interactions, a GST pull down experiment was performed to get better insights into the physical interaction between these proteins. The C- terminal region of *C*. *elegans* CASY-1C and the C- terminal without the kif interacting domain, CASY-1C(ΔKIF), were expressed as GST fusion proteins in bacteria. CASY-1C(ΔKIF) represents a deletion in the region harboring the two WDDS motifs and the acidic region (amino acids 70–148) in the C- terminal region of CASY-1C (deletion schematized in Fig 8B, top panel). GST alone, GST fused with CASY-1C and CASY-1C(ΔKIF) were used to precipitate HA- tagged UNC-104 protein expressed in a bacterial lysate. Full length CASY-1C significantly precipitated the 110 KDa C-terminal region of HA-tagged UNC-104 (Fig 8B). However, CASY-1C(ΔKIF) GST fusion did not show any precipitation of the HA-tagged UNC-104 (Fig 8C). This data further suggests that the C- terminal of CASY-1C interacts with the tail region of UNC-104. Additionally, UNC-104 and CASY-1C interaction was further validated using bimolecular fluorescence complementation (BiFC) assays, a method widely used to study protein-protein interactions in live animals. Here UNC-104 and CASY-1C were fused to a non-fluorescent YFP (Venus) hybrid also called ‘split-YFP’ (made up of the VN or VC of fluorescent YFP signals). Expression of YFP indicates that these two proteins are closely located (less than 7 nm apart) thus allowing for the fluorophore complementation to occur, hence leading to visible fluorescence [66–69]. As a positive control, an P*unc-104*::UNC-104::VN/ P*unc-104*::UNC-104::VC BiFC pair was microinjected as it has been well established that monomeric UNC-104 dimerizes to become functional and this dimerization can be tested using BiFC ([69] and Fig 8C). As a negative control, an P*unc-104*::UNC-104::VN/ empty::VC BiFC pair was examined. No fluorescence was detected in these transgenic *C*. *elegans*. The experimental transgenic line containing P*unc-104*::UNC-104::VN/ P*unc-25*::CASY-1C::VC showed significant YFP signal patterns along the GABAergic motor neurons on the VNC (Fig 8C). YFP fluorescence signals were significantly reduced in the transgenic animals containing P*unc-104*::UNC-104::VN/ P*unc-25*::CASY-1C(ΔKIF)::VC BiFC pair (Fig 8C). Finally we found that the expression of CASY-1C(ΔKIF) in GABAergic motor neurons could not rescue the Aldicarb phenotype in *casy-1* mutants (S8C Fig). These data strongly support functional *in vivo* interactions between UNC-104 and CASY-1C.

To further understand the role of UNC-104 in axonal trafficking of CASY-1, we determined the localization of CASY-1C::GFP in *unc-104* mutant animals. In WT *C*. *elegans*, CASY-1C::GFP shows significantly lower expression in the VNC cell bodies owing to its trafficking to the DNC synapses. However, in *unc-104* mutants we observed significant accumulation of CASY-1C::GFP in some neuronal cell bodies (Fig 8D). In parallel, we observed a significant decrease in the fluorescence intensity of CASY-1C::GFP at the DNC synapses (Fig 8E), suggesting that UNC-104 is a potential candidate for trafficking of CASY-1C::GFP to the synapse in these neurons. We next examined the motor neurons where CASY-1C::GFP is accumulating in the *unc-104* mutant animals. The localization pattern of P*unc-25*::mCherry with CASY-1C::GFP in the *unc-104* mutants revealed that CASY-1C accumulation in the *unc-104* mutants is not specific to GABAergic motor neurons as significant accumulation was observed in the adjacent cells that represent cholinergic motor neurons (arrow in S8D Fig).

Together, our results strongly suggests an interaction between the C-terminal of the shorter CASY-1 isoforms with the UNC-104 motor protein that appears to facilitate the transport of GABA SV precursors required to maintain inhibitory GABAergic signaling at the NMJ.

## Discussion

**Fig 9:**
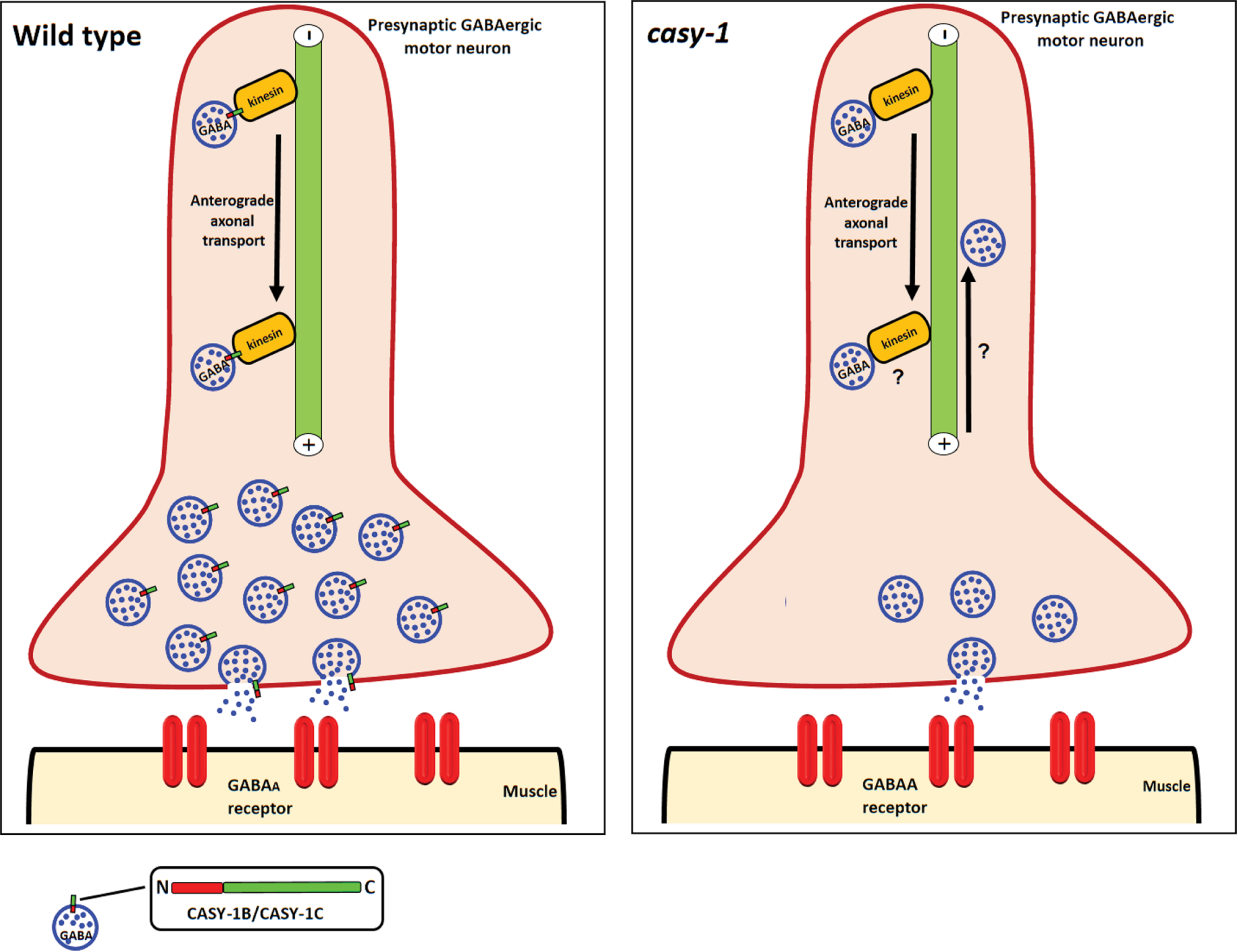
Proposed model for CASY-1 functioning at the NMJ. CASY-1B and CASY-1C, the shorter isoforms are present on the SV precursors. The conserved C-terminal of CASY-1 act as an adaptor to mediate interaction of GABA-specific SVs with the tail region of UNC-104/KIF1A motor protein that mediates fast anterograde axonal transport of synaptic cargo. Mobility dynamics of SV precursors in turn regulates the release kinetics of GABA at NMJ. However in the absence of *casy-1*, this cargo-adaptor-motor bridge is lost resulting in aberrant anterograde flux of the GABA-specific SV cargo along the axonal pathway. This function of CASY-1B/C shows some similarity to how the mammalian CLSTN1 is thought to function. CLSTN1 has been shown to be required for fast anterograde axonal transport and loss of interaction with kinesin motor results in decreased anterograde trafficking and an increase in retrograde transport of vesicular cargo [18].

GABA is a major inhibitory neurotransmitter functioning in both vertebrate and invertebrate nervous systems. In vertebrates, nearly 30–40% of the CNS synapses are thought to be GABAergic [70] and alterations in GABA neurotransmission have been associated with several different neurological disorders (reviewed in [71–73]). In *C*. *elegans*, very few reports have documented the role of genes regulating GABAergic synaptic transmission at the NMJ [74, 75]. In this study, we are proposing that the shorter CASY-1 isoforms are essential for regulating GABA signaling at the NMJ. Despite considerable reports emphasizing the involvement of calsyntenins in GABA synapse development and function, it has been very difficult to deduce the cellular and molecular mechanisms and implications of these proteins on animal behavior and function. Our results indicate that the short isoforms of CASY-1, CASY-1B and CASY-1C, are involved in modulating GABA synaptic release from motor neurons via interaction with the UNC-104 motor protein (Illustrated in Fig 9).

Mutants in *casy-1* show significant hypersensitivity to Aldicarb, suggesting increased synaptic transmission at the NMJ. Neuron-specific rescue experiments suggested that the shorter CASY-1 isoforms function specifically in GABAergic neurons to regulate synaptic signaling. Although the Aldicarb assay is a direct assay for monitoring defects in cholinergic signaling, it is also routinely used to identify mutants defective in GABAergic synaptic transmission. The increased hypersensitivity in Aldicarb assay, could result either from increased acetylcholine release (presynaptic defect) or increased expression of cholinergic receptors on the muscle (postsynaptic defect). Aldicarb hypersensitivity could also result from decreased GABA release (presynaptic defect) or decreased expression of GABA receptors (postsynaptic defect) [41, 76, 77]. Since motor circuit activity results from a balance between excitatory and inhibitory signaling at the NMJ, any defect in GABA signaling will result in a change in excitatory to inhibitory ratio, giving an Aldicarb phenotype [78–81]. Mutants in GABA signaling like *unc-25* (GABA-synthesis enzyme) and *unc-47* (GABA transporter) have been reported to be Aldicarb hypersensitive, due to overall increase in excitatory cholinergic signaling [41, 82, 83]. Our domain-mapping experiments suggest that the conserved C-terminal of CASY-1, present in *casy-1b/casy-1c* is functioning to rescue the hypersensitive phenotype of *casy-1* mutants. The expression analysis of CASY-1 isoforms further showed that only CASY-1B and CASY-1C express in GABAergic motor neurons, while CASY-1A expression is restricted mainly to head neurons and is not seen in the ventral cord motor neurons. Interestingly, *C*. *elegans* has devised a fascinating strategy wherein isoform expression and function is spatially regulated using alternative promoters. The shorter isoforms of CASY-1, which are essentially just the C-terminal region of mammalian Calsyntenins and required for this function, are expressed in GABAergic motor neurons to regulate GABA release at NMJ.

As discussed before, several studies of mammalian synapses using primary hippocampal neuronal cultures and knockout mice have established that calsyntenins are involved in the development and functioning of inhibitory GABA synapses [34]. CLSTN knockout mice also show reduced GABAergic neurotransmission [33] but the molecular basis of this regulation is unknown. In our study, we are showing, how the C-terminal of mammalian Calsyntenins which is conserved in CASY-1B/CASY-1C, can regulate GABAergic neurotransmission pre-synaptically. Using pharmacological, behavioral, optogenetic and electrophysiology approaches, we established defects in GABA signaling in *casy-1* mutants at the NMJ. All these mutant phenotypes could be completely rescued by expressing CASY-1B/CASY-1C specifically in GABAergic motor neurons. Although, mammalian calsyntenins are reported to be post-synaptic membrane proteins, here we are demonstrating a pre-synaptic role of the *C*. *elegans* orthologs of mammalian Calsyntenins. Our study opens up the possibility of exploring the potential existence of similar mechanisms regulating GABAergic neurotransmission in the mammalian nervous system.

We are also throwing light into the mechanistic insight of how the CASY-1 C-terminal could regulate GABA release at NMJ. Mammalian CLSTN1 has been well documented for its role in regulating trafficking of various axo-dendritic synaptic components via its interaction with kinesin light chain motor protein (*klc-1*). *C*. *elegans* CASY-1 has also recently been shown to interact physically with KLC-2 [59], suggesting a role for CASY-1 in trafficking of synaptic components as well. In this study, we have determined a novel interaction where the C-terminal of CASY-1 interacts with the tail region of the motor protein UNC-104 that essentially harbors the stalk region as well as the PH domain. UNC-104/KIF1A is established as an evolutionarily conserved motor protein required for trafficking of SV precursors from the soma to the synapse. Our results unlock the possible existence of similar interaction in mammalian system. However, we cannot nullify the possibility that CASY-1 might also affect SV precursor trafficking via its interaction with kinesin light chain (*klc-1* and *klc-2*) as multiple studies also showed the involvement of *klc-1* motor in SV precursor trafficking [84–86].

Despite proposing a convincing mechanistic role of *casy-1* isoforms in modulating GABA signaling at NMJ, some major questions remain unanswered. First, *casy-1b/casy-1c* specifically act in GABAergic neurons although they are also expressed in cholinergic neurons. Several previous reports have highlighted the role of molecules that expresses in both cholinergic and GABAergic motor neurons but specifically functions in just one system to affect NMJ signaling [87, 88]. Here we establish that *casy-1* isoforms function in GABAergic motor neurons to regulate Aldicarb responsiveness. However, we cannot rule out additional roles of CASY-1 in cholinergic motor neurons. Furthermore, electrophysiological recordings from the *casy-1* NMJ showed an increased EPSC frequency suggesting another mechanism for Aldicarb hypersensitivity, but expressing CASY-1 isoforms in cholinergic motor neurons could not rescue the Aldicarb phenotype. This implies that increased EPSC frequency is not an outcome of *casy-1* function in cholinergic motor neurons. Future investigations to address how CASY-1 could affect EPSC frequency might provide useful insights into other functions of CASY-1 isoforms that might affect neuromuscular signaling.

Despite the simplicity of *C*. *elegans* having just one gene coding for CASY-1, compared to multiple mammalian Calsyntenin genes, it emerges as an excellent regulator of diverse functions such as vesicular trafficking, functioning of GABAergic synapses and synaptic plasticity, functions that are performed by individual CLSTNs in the mammalian nervous system. This establishes *C*. *elegans* as an ideal model system to explore other functions of mammalian calsyntenins. Further studies in this system could enhance our understanding about pathophysiological mechanisms that trigger calsyntenin-related brain disorders.

## Methods

### *C*. *elegans* strain maintenance

All strains were maintained on nematode agar growth medium (NGM) plates seeded with OP50 *Escherichia coli* at 20°C under standard conditions [89]. The *C*. *elegans* Bristol strain, N2 was used as the wild-type (WT) control. Strains were synchronized by hypochlorite treatment followed by allowing *C*. *elegans* to grow for approximately 2.5 days at 20°C. All experiments were carried out with young adult hermaphrodites at ~ 23°C, unless otherwise mentioned. A complete list of strains utilized in this study is given in Tables C and D in S1 Text. OP50 *Escherichia coli* was obtained from the *C*. *elegans* Genetics Center (University of Minnesota, Minneapolis, MN, USA).

### Transgenic strains and constructs

Tables A and B in S1 Text lists all the plasmids and constructs used in this study, Table E in S1 Text lists the primers used to perform genotyping and Table F in S1 Text lists the primers used to make the different transgenes used in this study. All the plasmids were generated using standard restriction digestion based cloning strategy and sequenced before use in experiments. Previously described microinjection techniques were used to generate stable transgenic *C*. *elegans* lines carrying extra- chromosomal DNA arrays using either p*myo-3*::mCherry, p*myo-2*::GFP or p*opt-3*::mCherry as co-injection markers [90].

The three *casy-1* isoforms studied in the manuscript are mentioned on the Wormbase based on EST evidence. The sequence information has been obtained from Wormbase to design isoform-specific primers to amplify the promoter sequences (~ 3 kb upstream of the translation start codon) as well as the coding region.

### Pharmacological assays

All the assays were performed with the experimenter blind to the genotypes. Each assay was performed at least three times as indicated at the base of each bar with > than 20 animals for each replicate.

#### Aldicarb assay

The Aldicarb assays were performed as described previously [42]. Briefly, fresh Aldicarb plates were made previous day by adding 100mM stock solution (prepared in ethanol) of Aldicarb (Sigma-Aldrich, USA) to molten NGM at a final concentration of 1mM. Plates were then seeded with OP50 *E*. *coli* and stored in dark at room temperature overnight. For each assay, ~ 20 young adult hermaphrodites were transferred on to Aldicarb plates and scored for paralysis every 15 minutes for more than 2.5 hours. Animals were considered paralyzed when they failed to show any body bends following prodding three times on the head. For heat shock experiments, *C*. *elegans* were grown till young adult stage, heat shock was given at 34°C for 1 hour, following recovery for 3 hours at 20°C. Aldicarb assay was performed after recovery period is over.

#### Levamisole assay

The Levamisole assay was performed by exposing *C*. *elegans* to Levamisole (Sigma-Aldrich, St. Louis, MO) at a final concentration of 0.5mM. Plates were prepared as described previously for Aldicarb assay. Young adult hermaphrodites were transferred on to Levamisole plates and scored for paralysis every 15 minutes. Animals were deemed paralyzed when they do not show any body bends following three prodding’s on the head [42].

#### Pentazylenetetrazole (PTZ) assay

PTZ assays were performed as described previously [76]. Plates were made fresh on the day of experiment by spreading PTZ stock solution (100 mg/ml) on solidified NGM plates at a final concentration of 10 mg/ml. The plates were allowed to dry for about 2 hours and then seeded with concentrated OP50 *Escherichia coli*. For each assay, ~ 10 young adult hermaphrodites were transferred to PTZ plates and then scored by visual inspection under a stereomicroscope for the presence of anterior head bends called ‘head bobs’ at 30 and 60 minute time points. *unc-25* mutant was used as positive control.

#### Muscimol assay

Muscimol assays were carried out with young adult hermaphrodites as previously described. Briefly, ~ 10 *C*. *elegans* were placed on NGM plates containing 0.5mM Muscimol. The animals were then scored visually after 30 minutes by observing under a stereomicroscope for the presence of the “rubber band” phenotype [48, 79].

### Imaging experiments

Animals were immobilized with 30mg/ml 2,3-butanedione monoxamine (Sigma) on 2% agarose pads. All quantitative imaging was done using Zeiss AxioImager microscope with a 40x or 63x 1.4 NA Plan APOCHROMAT objective equipped with a Zeiss AxioCam MRm CCD camera controlled by Axiovision software (Zeiss Micro-imaging). For comparing WT animals with *casy-1 mutants*, >25 *C*. *elegans* were analyzed for each genotype [38].

For the morphological analysis of GABAergic neurons (*juIs76* [P*unc-25*::GFP]) and cholinergic neurons (*nuIs321* [P*unc-17*::mCherry]), z-stacks of the entire *C*. *elegans* (from overlapping fields of view) were taken using a 63x objective and a 10 μm optical slice (20 slices at 0.5 micron distance). Image J software was used to derive maximum intensity projection images from z-stacks. These images were then analyzed for gross morphological analysis of neurons.

All fluorescent SV marker imaging was done with the 63x objective. Animals were imaged for the DNC in the posterior portion of the *C*. *elegans* halfway between the vulva and the tail. For fluorescent analysis, image stacks were taken (approximately 10 μm) and the maximum intensity projections were obtained using Image J software. For the analysis of fluorescence intensity a freehand line was drawn (approximately 100 μm) along the DNC and the intensity values were obtained for each animal indicated. These values were normalized to WT levels and then an average plot has been drawn to determine the statistical difference in the intensity. For all the quantitative analysis, identical camera gain, exposure settings, and fluorescence filters were used for a particular transgenic line. For all the Figures, an average of the values for each *C*. *elegans* in the data set ± S.E.M. is plotted. Statistical difference between WT and mutant values was determined using the Student's *t*-test (*p* ≤ 0.05) in Graph Pad Prism 7. Graphs of punctal and axonal fluorescence show data normalized to WT values.

Transcriptional reporter expression, *superecliptic pHluorin*, *BiFC* and co-localization imaging was done using Leica HC PL APO 63x/ TCS SP8 confocal microscope (Leica Microsystems) with Multi-Ar (457, 488, and 515 nm), and He-Ne (543 and 633 nm) laser lines and HyD detectors. For co-localization analysis, image stacks were taken (four times of average for 1024x1024 scan format at speed 400 Hz) and maximum intensity projections were obtained for each channel separately. Maximum intensity projections were then merged to obtain co-localization images using the Image J software.

### Electrophysiology

Strains for electrophysiology were maintained on plates seeded with HB101 *Escherichia coli* at 20°C. Adult *C*. *elegans* were immobilized on Sylgard coated coverslips with cyanoacrylate glue. Dissections and whole-cell recordings were performed as described previously [91–93]. Statistical significance was determined using the one-way ANOVA followed by Dunnett’s test for comparison of mean frequency and amplitude for endogenous EPSCs and IPSCs.

### Optogenetics

Optogenetic experiments were performed as described previously [94]. All- trans retinal (ATR) plates were prepared fresh and used within one week. ATR plates were made by spotting NGM plates with 50 μl of OP50 *E*. *coli* containing 0.8mM ATR and allowed to grow overnight at 37° C in dark. Control plates were made by seeding NGM plates with 50μl of OP50 lacking ATR. Transgenic animals carrying GABAergic Channelrhodopsin *zxIs3* [P*unc-47*:: ChR2(H134R)::YFP + lin-15(+)] were synchronized and grown on ± ATR plates at 20°C. For the assay, ~ 10 young adult hermaphrodites were picked on fresh seeded NGM plates. For all analysis, a 20- second video was made using the Zeiss Lumar V12 fluorescence Stereomicroscope. Video recording was started as soon as the animals were seen crawling, blue light was turned on within 4 seconds and then left on for the duration of recording. The light intensity was approximately 57.5 mW/cm^2^ from HXP 120V light source. Two frames were selected from all the videos for each animal, one before blue light was turned on and one after that with the maximum relaxation of the *C*. *elegans*. Analysis was performed as described previously [54]. In brief, Image J on a Wacom Bamboo tablet and stylus were used to trace a freehand line from the nose tip down to the posterior-most point of the *C*. *elegans*. The length of the animal before and after exposure to blue light was measured and difference in these lengths divided by the starting *C*. *elegans* length was determined. This data was then plotted as percentage change in body length (relaxation).

### Fluorescence recovery after photobleaching (FRAP)

FRAP was performed on transgenic WT and *casy-1 (tm718)* mutants carrying GABAergic *nuIs376* [P*unc-25*:: SNB-1::GFP] or cholinergic *nuIs152* [P*unc-129*::SNB-1::GFP]. For RNAi- based FRAP experiments, the F2 generation of *C*. *elegans* exposed to *unc-104* RNAi were used. FRAP experiments were carried out using Leica TCS SP8 confocal microscope. For the FRAP experiments, *C*. *elegans* were immobilized on 10% agarose pads containing 0.2μl of 0.1μm polystyrene microspheres (Polysciences). To account for the focal drift, image stacks were taken, and maximum intensity projections were obtained using Image J. For this experiment, three frames were taken for one representative puncta in the posterior half of the animal, followed by 100 iterations of photobleaching in the defined region (25% power of a 488 nm Argon laser with bleaching power of 45%). This was followed by monitoring 10 iterations of recovery every 25 seconds for up to six minutes. The intensities were normalized against a non-bleached ROI within the same animal.

### RNA interference (RNAi)

RNAi experiments were carried out as described previously [95]. All assays were performed in *eri-1(mg366); lin-15B(n744)* background which makes animals more sensitive to RNAi in the nervous system[96]. Briefly, animals were raised on bacteria expressing double-stranded RNA containing *casy-1*, *unc-104* or empty vector for two generations. F2 generation animals were analyzed on acute Aldicarb assays in duplicates.

### Glutathione S-transferase pull down assay

For GST- pull down experiment soluble C-terminal of *casy-1* cDNA (amino acids 48–160) and *casy-1(Δkif)* cDNA was subcloned into the pGEX-KG vector and expressed as N-terminal GST fusion proteins in *Escherichia coli* BL21 cells. Cells were grown at 37°C to an OD_600_ nm of 0.6, induced with 0.1 mM isopropylthiogalactoside (IPTG) and grown further for 16 hours at 18°C. Harvested cells were resuspended in a lysis buffer containing 50 mM Tris at pH 7.5, 300 mM NaCl, Protease inhibitor (Roche) and DNase I (Sigma) and disrupted by freeze–thawing followed by short sonication. The cell lysate was centrifuged at 14,000 rpm for 20 minutes at 4°C and the cleared supernatant was incubated with glutathione beads (Sigma) for 2 hours at 4°C. The beads were washed with lysis buffer three times. The beads were then packed into a syringe column. GST-tagged protein immobilized on glutathione–Sepharose beads was incubated with the supernatant from *E*.*coli* BL21 cells expressing UNC-104-HA for 2 hours at 4°C. The supernatant was prepared as mentioned above. The beads were then washed extensively five times. After extensive washing with lysis buffer, bound proteins were added directly into 5×SDS/PAGE sample buffer, boiled for 5 min, separated by SDS/PAGE and subjected to immunoblot analysis. Western blot using Anti-HA Antibody (1∶1000) was then performed to identify interaction between CASY-1 and UNC-104.

### Bimolecular fluorescence complementation (BiFC) assays

For BiFC studies, transgenic strains containing following BiFC pairs were generated (1) *Punc-104*::UNC-104::VN173 and *Punc-104*::UNC-104::VC155 (positive control) (2) *Punc-104*::UNC-104::VN173 and Empty VC155 (negative control) (3) *Punc-104*::UNC-104::VN173 and *Punc-25*::CASY-1C (amino acids 1–160)::VC155 and (4) *Punc-104*::UNC-104::VN173 and *Punc-25*::CASY-1C (ΔKIF)::VC155. The transgenic lines were imaged using Leica HC PL APO 63x/ TCS SP8 confocal microscope in the posterior portion of the *C*. *elegans* halfway between the vulva and the tail. The transgenic lines obtained showed a discontinuous expression along the entire VNC probably due to low efficiency of extrachromosomal arrays.

### Live imaging and analysis of SV transport dynamics

For in vivo time-lapse imaging of the motor neuron commissures, 1-day adult hermaphrodites with the GABAergic *nuIs376* [P*unc-25*::SNB-1::GFP] or cholinergic *nuIs152* [P*unc-129*::SNB-1::GFP] transgene were immobilized in 3 mM tetramisole in M9 and mounted on a 5% agarose pad. Imaging was performed in the posterior commissures. SNB-1::GFP time-lapse images were obtained with an Olympus IX83 microscope (Olympus, Tokyo, Japan) using a Plan Apochromat objective (100X, 1.4 NA) attached with a spinning disk confocal head (CSU22; Yokogawa, Tokyo, Japan) and equipped with an electron-multiplying charge-coupled device (EMCCD) camera (ImagEM X2 EM-CCD, Hamamatsu). A 488 nm laser (25 mW) was used at 10% power for imaging (100X objective, 300 ms exposure time). Moving particles were defined as puncta that were displaced by more than 3 pixels in less than 5 consecutive time frames. The flux of particles was calculated as the total number of puncta moving in either direction in an entire kymograph, then normalized to a 10 μm region, further normalized to time (1 minute). The Unit of flux here is number of events/10μm/min. Movies were acquired at a constant frame rate of 3 frames/s for a total of 700 (again average number- varies across movies) frames. Each frame was 512x512 pixels dimension. Kymographs were generated and analyzed using ImageJ software, version 1.41 (National Institutes of Health, Bethesda, MD), and statistical significance was determined using a two-way ANOVA with Bonferroni's multiple comparison post-test.

### Statistical analysis

All statistical analysis were performed using GraphPad Prism V7. Experimental data are shown as mean ± S.E.M. Statistical comparisons were done using the Student's *t*-test, two-way ANOVA or one-way ANOVA with Bonferroni's multiple comparison or Dunnett’s post-test. A level of *p*<0.05 was considered significant.

## Supporting information

S1 Fig Expression of all *casy-1* isoforms is reduced in the *casy-1(tm718)* mutant line. (A) A schematic of the CASY-1 isoforms expressed in *C*. *elegans*. CASY-1A is a full-length protein with all the conserved domains- signal peptide (SP), two tandem cadherin domains, LG/LNS domain, transmembrane region and cytosolic acidic region. CASY-1B and CASY-1C are truncated proteins lacking the extracellular LG/LNS domain as well as cadherin repeats. CASY-1B and CASY-1C differ in just 7 amino acids at the N- terminal region, which are extra in CASY-1B. (B) Expression levels of *casy-1* isoforms; *casy-1a*, *casy-1b* and *casy-1c/casy-1a* in wild-type (WT) and *casy-1(tm718)* mutant background in a mixed stage population of *C*. *elegans*. Fold change mRNA levels are indicated after normalization with *act-1* levels, which served as an internal control. Real time PCR was done thrice from independent RNA samples and the data was analyzed using regular two-way ANOVA in GraphPad Prism V7 software.
(TIF)

S2 Fig Cholinergic motor neurons are normal in *casy-1* mutants. (A) Representative fluorescent images of WT and *casy-1* mutant animals expressing mCherry in all cholinergic neurons (*nuIs321* [P*unc-17*::mCherry]). The number of cell bodies and axonal commissures were largely normal in *casy-1* mutants (n-28). Scale bar, 8 μm. (B) Clustal omega alignment of the C-terminal of the three *casy-1* isoforms. All the isoforms are identical in their C-terminal. Purple and green sequences denote the transmembrane domain and the acidic region in CASY-1 respectively. Red denotes the six amino acids that are present in CASY-1B but not in CASY-1C.
(TIF)

S3 Fig Pre- and post-synaptic synapse morphology is normal in *casy-1* mutants. (A) *casy-1* mutants show higher sensitivity to GABA receptor antagonist PTZ than the WT animals. The graph shows the fraction of animals showing anterior ‘head bobs’ after 30 minute and 60 minute exposure to 10 mg/ml PTZ. Mutants in *unc-25*, the GABA synthesis enzyme were used as positive controls. Assays were done (~10 *C*. *elegans*/assay) thrice. Data are represented as mean ± S.E.M. (***P<0.0001 using one-way ANOVA and Bonferroni's Multiple Comparison Test). (B) Representative fluorescent images of GABAergic SNB-1::GFP [*nuIs376* (P*unc-25*::SNB-1::GFP)] on the VNC cell bodies of WT or *casy-1* mutant. Scale bar, 10μm. The ratio of DNC synapse fluorescence intensity to VNC cell body intensity at GABAergic synapses showed a subtle but significant decrease in fluorescent intensity when compared to WT animals. This suggests that less number of GABA vesicles are present at the DNC synapses. Quantification of fluorescent intensity is normalized to WT values. The number of animals analyzed for each genotype is indicated at the base of the bar graph. Quantified data are displayed as mean ± S.E.M. and were analyzed by two-tailed Student’s *t*-test (*p<0.01). Representative fluorescent images of cholinergic (C) [*nuIs160* (P*unc-129*:: SYD-2::GFP)] or (D) GABAergic [*hpIs3* (P*unc-25*::SYD-2::GFP)] synapses in the dorsal cord of WT and *casy-1* mutant *C*. *elegans*. The density of cholinergic and GABAergic synapses was largely normal in the mutants, indicating normal synapse development. Representative fluorescent images of (E) Cholinergic [*nuIs299* (P*myo-3*::ACR-16::GFP)] or (F) GABAergic [*nuIs283* (P*myo-3*::UNC-49::GFP)] receptors in the muscle of WT and *casy-1* mutants. The expression of receptors is unaltered in *casy-1* mutants. Quantification of fluorescent intensity is normalized to WT values. The number of animals analyzed for each genotype is indicated at the base of the bar graph. Quantified data are displayed as mean ± S.E.M. and were analyzed by two-tailed Student’s *t*-test, “ns” indicates not significant in all figures. Scale bar, 8 μm. (G) *casy-1* mutants show normal muscle response to 0.5 mM Muscimol, a GABA receptor agonist. Assays were done three times and total number of animals analyzed for each genotype is indicated at the base of the bar graph. Quantified data are displayed as mean ± S.E.M. and were analyzed by two-tailed Student’s *t*-test. (H) *casy-1* mutants show normal muscle response to 0.5 mM Levamisole, a cholinergic receptor agonist. Assays were done three times (~20 *C*. *elegans*/assay, see the Methods section for a more detailed protocol).
(TIF)

S4 Fig CASY-1A isoform could not rescue the decreased IPSC defect in *casy-1* mutants. mEPSCs and mIPSCs were recorded from body wall muscles of adult animals for the indicated genotypes. Representative traces of mIPSCs and summary data for frequency and amplitude are shown. Expression of CASY-1A isoform under its endogenous promoter could not rescue the decreased IPSC frequency in *casy-1* mutants. This data clearly suggests that CASY-1A isoform do not function to regulate GABA synaptic transmission at the NMJ. The number of animals analyzed for each genotype is indicated. Data are represented as mean ± S.E.M. (*p<0.05 using the one-way ANOVA with Dunnett’s post test, “ns” indicates not significant in all Figures).
(TIF)

S5 Fig Shorter CASY-1 isoforms are cleaved at the synaptic cleft to release ecto-domains. (A) Aldicarb-induced paralysis in *casy-1* mutants is completely rescued by expressing GFP or mCherry-tagged CASY-1C expressed under their own promoter, suggesting that the tagged versions of CASY-1C are functional. Assays were done three times as indicated in the figures (~20 *C*. *elegans*/assay). Data are represented as mean ± S.E.M. *** indicates *p*<0.0001 using one-way ANOVA and Bonferroni's Multiple Comparison Test and “ns” indicates not significant in comparison to WT. (B) The upper panel shows a schematic showing the processing of *casy-1b* and *casy-1c* isoforms at the NMJ. *casy-1a* isoform has been shown to be cleaved at its N-terminal by extracellular peptidases resulting in the secretion of entire N- terminal domain [21]. P*casy-1b/c*::mCherry::CASY-1B/C translational reporter shows that CASY-1B and CASY-1C isoforms, which lack a validated signal sequence also reach the synapse where their N- terminal is cleaved resulting in the release of entire N- terminal into the synaptic cleft. The lower panel shows representative fluorescent images showing the release of mCherry tagged N-terminal of CASY-1B and CASY-1C at the synapse followed by their uptake in the coelomocytes (solid arrowheads). Scale bar, 25μm.
(TIF)

S6 Fig Mobility dynamics of cholinergic::SNB-1 is normal in *casy-1* mutants. Representative fractional recovery fluorescence trace for a single *nuIs152* [P*unc-129*::SNB-1::GFP] puncta after photobleaching. Recovery was measured with the pre-bleach fluorescence intensity being 100% and the post-bleach intensity at time 0 being 0%. Recovery rate for Cholinergic SNB-1 puncta is unaltered in *casy-1* mutant.
(TIF)

S7 Fig Diverse vesicular cargo are not affected in *casy-1* mutants. (A) Representative image for Mitochondrial marker (P*unc-25*::MITO::GFP) in GABAergic motor neurons in WT and *casy-1* mutants. (B) Representative image for early endosomal marker [*juIs198* (P*unc-25*:: YFP::RAB-5)] in GABAergic motor neurons in WT and *casy-1* mutants. (C) Representative image for Lysosomal marker (P*unc-25*::CTNS-1::GFP) in GABAergic motor neurons of WT and *casy-1* mutants. Scale bar, 10μm. The fluorescence intensity for mitochondrial and early endosomal marker are largely normal in *casy-1* mutants, while lysosomal marker showed a subtle but significant decrease in fluorescent intensity when compared to WT animals. Quantification of fluorescent intensity is normalized to WT values. The number of animals analyzed for each genotype is indicated at the base of the bar graph. Quantified data are displayed as mean ± S.E.M. (*p<0.05 using two-tailed Student’s *t*-test, “ns” indicates not significant in all Figures).
(TIF)

S8 Fig The CASY-1 C–terminal serves as a potential adaptor for *unc-104/kif1a* mediated cargo transport. (A) Aldicarb- induced paralysis was compared following RNAi treatments with control (empty vector), *unc-104* or *casy-1* vectors as indicated in the graph. Knockdown of *unc-104* results in a similar Aldicarb resistance phenotype in both the WT and *casy-1* mutants indicating that *casy-1* and *unc-104* could be interacting genetically, however, this could also result from the dominant phenotype of *unc-104* function at the synapse. Assays were done 4 times as indicated in Figure (~20 *C*. *elegans*/ assay). Data are represented as mean ± S.E.M. (***p<0.0001 using one-way ANOVA and Bonferroni's Multiple Comparison Test). (B) The left panel indicates a schematic representation of CASY-1C C-terminal and UNC-104 constructs utilized in this study. UNC-104 constructs cover several domains; the UNC-104—Motor domain (aa 1–356), the fork head domain FHA (aa 460–633), the SYD-2/liprin binding domain STALK (aa 623–1105) and the PH domain (aa 1087–1628). The lower panel on the right indicates the yeast two hybrid data showing the control spotting in the presence of a selection component while the upper panel shows significant interaction of CASY-1 C terminal with several UNC-104 domains. The most prominent interaction occurs with the tail region of UNC-104 spanning the stalk and PH domain. (C) The C-terminal of the CASY-1C isoform harboring the KIF-interacting domain is required to regulate synaptic transmission at the NMJ. Transgenic lines expressing CASY-1C (ΔKIF) under *unc-25* promoter could not rescue the Aldicarb hypersensitivity in *casy-1* mutants. Assays were done 3 times as indicated in Figures (~20 *C*. *elegans*/ assay). Data are represented as mean ± S.E.M. (***p<0.0001 using one-way ANOVA and Bonferroni's Multiple Comparison Test). “ns” indicates not significant in all Figures. (D) Representative fluorescent images of P*casy-1c*::CASY-1C::GFP colocalizing in the VNC with P*unc-25*::mCherry in the *unc-104* mutant. Colocalization data suggests that the accumulation of CASY-1C::GFP occurs in both Cholinergic and GABAergic cell bodies in the VNC of *unc-104* mutants. Scale bar, 10 μm.
(TIF)

S1 Movie WT *C*. *elegans* move normally on brief exposure to PTZ. This media file indicates a 6s clips of WT animals (7 frames/second) exposed to 10 mM PTZ for 30 minutes. As can be seen from the movie file the WT *C*. *elegans* shows normal sinusoidal locomotion after 30 minutes exposure to PTZ.
(AVI)

S2 Movie *casy-1* mutants show anterior convulsions on exposure to PTZ. This media file indicates a 6s clips of *casy-1* mutant animals (7 frames/second) exposed to 10 mM PTZ for 30 minutes. As can be seen from the media file, one *casy-1* mutant shows anterior convulsions or ‘head-bobs’ while another animal shows the tail shrinker phenotype characteristic of GABA mutants in *C*. *elegans*.
(AVI)

S3 Movie *casy-1* mutants, with GABAergic CASY-1C expression show normal locomotion on exposure to PTZ. The media file indicate a 6s clips of *casy-1*; P*unc-25*::CASY-1C *C*. *elegans* (7 frames/second) exposed to 10 mM PTZ for 30 minutes. Expressing the CASY-1C isoform in GABAergic neurons completely rescues the PTZ defects seen in the *casy-1* mutant animals.
(AVI)

S4 Movie Movie of the synaptic vesicle marker SNB-1::GFP trafficking in GABAergic neurons in WT *C*. *elegans*. Representative time-lapse recording of SNB-1::GFP in axons of WT GABAergic commissure. The media file represents a 10s clips at 7 frames/second. VNC is on the right.
(MOV)

S5 Movie Movie of the synaptic vesicle marker SNB-1::GFP trafficking in GABAergic neurons in the *casy-1* mutants. Representative time-lapse recording of SNB-1::GFP in axons of *casy-1* mutant GABAergic commissure. The media file represents a 14s clips at 7 frames/second. VNC is on the right.
(MOV)

S6 Movie Movie of the synaptic vesicle marker SNB-1::GFP trafficking in GABAergic neurons in *casy-1* mutants that express CASY-1C in GABAergic neurons (GABA rescue of *casy-1*). Representative time-lapse recording of SNB-1::GFP in axons of rescue line GABAergic commissure. The media file represents a 10s clips at 7 frames/second. VNC is on the top.
(MOV)

S7 Movie Movie of the synaptic vesicle marker SNB-1::GFP trafficking in cholinergic neurons in WT animals. Representative time-lapse recording of SNB-1::GFP in axons of WT cholinergic commissure. The media file represents a 14s clips at 7 frames/second. VNC is on the right.
(MOV)

S8 Movie Movie of the synaptic vesicle marker SNB-1::GFP trafficking in cholinergic neurons in the *casy-1* mutants. Representative time-lapse recording of SNB-1::GFP in axons of *casy-1* mutant cholinergic commissure. The media file represents a 14s clips at 7 frames/second. VNC is on the down-left.
(MOV)

S1 Text Supplemental methods and tables. (DOCX)
