# Correction: The C-terminal of CASY-1/Calsyntenin regulates GABAergic synaptic transmission at the *Caenorhabditis elegans* neuromuscular junction

**DOI:** 10.1371/journal.pgen.1012023

**Published:** 2026-01-20

**Authors:** Shruti Thapliyal, Amruta Vasudevan, Yongming Dong, Jihong Bai, Sandhya P. Koushika, Kavita Babu

After publication of this article [1], errors were identified in Figs 1, 5, and S7.

In the originally published Figs 1E and 5A:

- In the Gonadal Sheath column of Fig 1E, the P*casy-1c*::GFP panel is incorrect and is a duplicate of the P*casy-1b*::GFP panel.
- The P*GABA*::mCherry panel in the first column of Fig 5A contains a movement artifact.
- The lower P*casy-1b*::GFP and associated P*GABA*::mCherry and Merge panels, and the lower P*casy-1c*::GFP panel and the associated P*GABA*::mCherry and Merge panels of Fig 5A, were incorrectly switched with each other during figure compilation and are therefore incorrectly placed and labeled in the originally published Fig 5A.

With this Correction, the first author provides revised Figs 1 and 5 including the correct image for the P*casy-1c*::GFP panel of Fig 1E from the original experiments.

Regarding the P*GABA*::mCherry panel in the first column of Fig 5A, the first author stated that at approximately z-stack position #7, the worm underwent a slight movement, creating the impression of two cell bodies in the maximum intensity projection (MIP) of all stacks. With this Correction, the P*GABA*::mCherry, P*casy-1a*::GFP and Merge panels are replaced with a reduced number of images of the same image stack from after the movement occurred, to remove this artifact present in the merged image used in the published panel. Based on the explanation and underlying image data provided, which is supportive of the revised figure, PLOS considers this concern resolved.

The first author confirms that the same images used to represent the P*casy-1b*::GFP and P*casy-1c*::GFP panels in the Ventral Nerve Cord column of Fig 1E are also used in the lower part of Fig 5A (now correctly aligned in the revised Fig 5) and that the images correctly represent the labeled conditions in each revised figure. This is also reflected in the updated figure legend for Fig 1.

In both S7A and S7C Figs, one additional WT sample was incorrectly included during the preparation of the bar graphs, resulting in incorrect raw WT data and subsequently also *casy-1* data, which are normalized to WT data. The correct number of WT samples in S7A Fig is 17, and in S7C Fig is 13. The corresponding author confirmed that the changes in S7A and S7C Figs do not change the significance of the results presented. With this Correction, they provide the corrected bar graphs in a revised S7 Fig.

The first and corresponding authors have shared the original images underlying all panels in Figs 1, 2, 5, S2, and S7 in S1-5 Files and the individual-level quantitative data underlying the updated Figs 1 and S7 are provided in S6–7 Files. The first author stated that all other underlying data are available and can be provided upon request.

With this Correction the *PLOS Genetics* Editors inform readers that there was a potential competing interest between the authors and one or more people involved in peer review. After reviewing this matter PLOS concluded that the article’s publication is supported based on expert input that was not affected by the concern. We regret that the issues were not addressed prior to the article’s publication.

**Fig 1:**
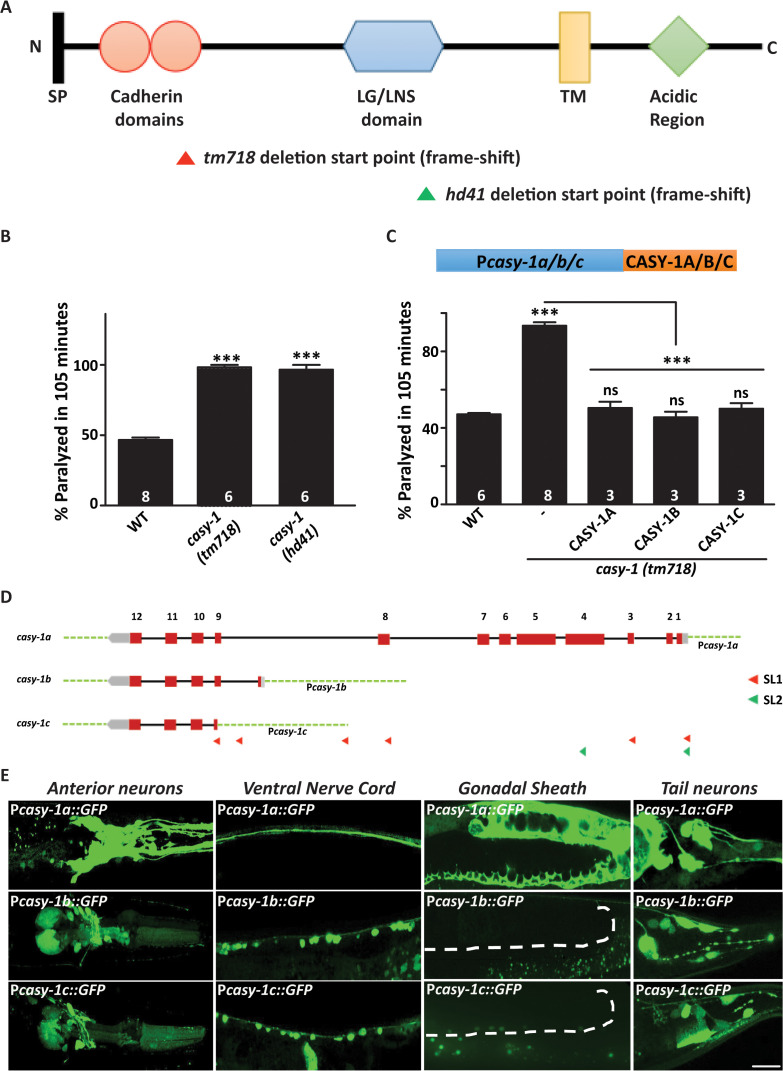
**(A)** A schematic representation of the CASY-1 protein showing the N-terminal signal peptide (SP), two-tandem cadherin repeats, LG/LNS domain, transmembrane region (TM) and cytosolic acidic region.The domains deleted in *casy-1 mutants* are indicated as triangles. The *tm718* and *hd41* alleles are putative null alleles as deletion starts in the N-terminal region and results in a frame-shift in both cases. **(B)** Aldicarb-induced paralysis in *casy-1* mutants was compared to wild-type (WT) animals. Both *casy-1* mutant alleles (*tm718* and *hd41*) are hypersensitive in the Aldicarb assays. Assays were done at least 6 times. **(C)** A schematic representing the transgenes used in the experiment. Expression of *casy-1* isoforms under their endogenous promoters completely rescues the Aldicarb hypersensitivity of *casy-1* mutant animals. In B and C, number of assays (~20 *C*. *elegans*/assay) is indicated for each genotype. Data are represented as mean ± S.E.M. (***p < 0.0001 using one-way ANOVA and Bonferroni’s Multiple Comparison Test) “ns” indicates not significant in all Figures. **(D)** Pictorial representation of the genomic locus of three isoforms. CASY-1B and CASY-1C are expressed by alternative promoters that exist in between the 8^th^ and 9^th^ intron of CASY-1A isoform, which is unusually long (~ 4000 bp) and carries their own SL1 leader sequences. The location of promoter sequences utilized in the study are indicated. **(E)** Representative confocal images of transcriptional reporters of the three *casy-1* isoforms. Expression of GFP under isoform-specific promoters showed expression of *casy-1a* in most of the head neurons including amphid sensory neurons, in VNC, some tail neurons, in the intestine as well as in the gonadal sheath. *casy-1b* and *casy-1c* also showed expression in some head neurons, in the ventral cord motor neurons and some tail neurons but no expression in the gonadal sheath. Dotted lines indicate the position of gonadal sheath. Scale bar, 20μm. The images used for P*casy-1b*::GFP and P*casy-1c*::GFP in this panel have also been used in Fig 5A in the colocalization with P*GABA*::mcherry.

**Fig 5:**
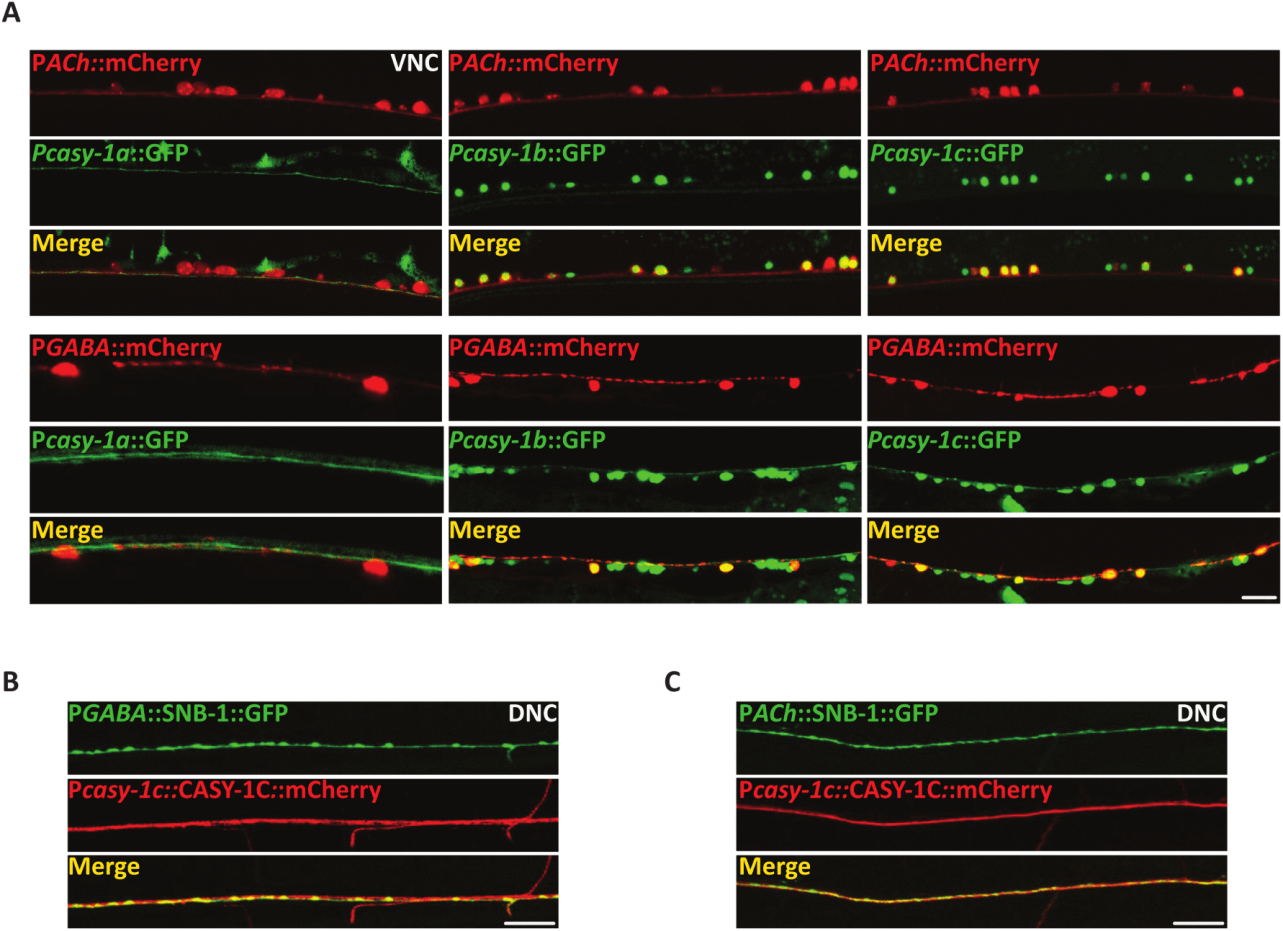
**(A)** Expression of GFP under isoform-specific *casy-1* promoters. *casy-1a* transcriptional reporter does not co-localize with mCherry marked cholinergic or GABAergic motor neurons. *casy-1b* and *casy-1c* expression reporters show expression in both cholinergic and GABAergic motor neurons.Anterior is to the left in all panels. Scale bar, 8μm. **(B)** Representative fluorescent images of P*casy-1c*::CASY-1C::mCherry translational reporter showing co-localization with the GABAergic *nuIs376* [P*unc-25*::SNB-1::GFP] pre-synaptic markers suggesting the presence of CASY-1C in the GABAergic NMJ pre-synaptic termini. Scale bar, 10μm. **(C)** Representative fluorescent images of P*casy-1c*::CASY-1C::mCherry translational reporter showing co-localization with the cholinergic *nuIs152* [P*unc-2129*::SNB-1::GFP] pre-synaptic markers suggesting the presence of CASY-1C in the cholinergic synapses. Scale bar, 10μm.

## Supporting information

S7 Fig (A) Representative image for Mitochondrial marker (P*unc-25*::MITO::GFP) in GABAergic motor neurons in WT and *casy-1* mutants. (B) Representative image for early endosomal marker [*juIs198* (P*unc-25*:: YFP::RAB-5)] in GABAergic motor neurons in WT and *casy-1* mutants. (C) Representative image for Lysosomal marker (P*unc-25*::CTNS-1::GFP) in GABAergic motor neurons of WT and *casy-1* mutants. Scale bar, 10μm. The fluorescence intensity for mitochondrial and early endosomal marker are largely normal in *casy-1* mutants, while lysosomal marker showed a subtle but significant decrease in fluorescent intensity when compared to WT animals. Quantification of fluorescent intensity is normalized to WT values. The number of animals analyzed for each genotype is indicated at the base of the bar graph. Quantified data are displayed as mean ± S.E.M. (*p < 0.05 using two-tailed Student’s *t*-test, “ns” indicates not significant in all Figures).
(TIF)

S1 File Fig 1E underlying image data. This file includes the original images underlying Fig 1E. Each image file included in S1 File is generated from image stacks taken in the original experiments.
(ZIP)

S2 File Fig 2A underlying image data. This file includes the original images underlying Fig 2A. Each image file included in S2 File is generated from image stacks taken in the original experiments.
(ZIP)

S3 File Fig 5 underlying image data. This file includes the original images underlying Fig 5. Each image file included in S3 File is generated from image stacks taken in the original experiments.
(ZIP)

S4 File Fig S2A underlying image data. This file includes the original images underlying S2A Fig. Each image file included in S4 File is generated from image stacks taken in the original experiments.
(ZIP)

S5 File Fig S7 underlying image data. This file includes the original images underlying S7A-C Figs. Each image file included in S5 File is generated from image stacks taken in the original experiments.
(ZIP)

S6 File Fig 1 individual-level quantitative data. This file includes the underlying individual-level quantitative data underlying Figs 1B and 1C.
(XLSX)

S7 File Fig S7 individual-level quantitative data. This file includes the underlying individual-level quantitative data underlying S7A-C Figs, including the raw and normalized data.
(XLSX)
