# Supplementary figures and images for: The C-terminal of CASY-1/Calsyntenin regulates GABAergic synaptic transmission at the Caenorhabditis elegans neuromuscular junction
Source: PLoS Genet. 2018 Mar 12;14(3):e1007263. doi: 10.1371/journal.pgen.1007263 (PMC5864096; doi:10.1371/journal.pgen.1007263)

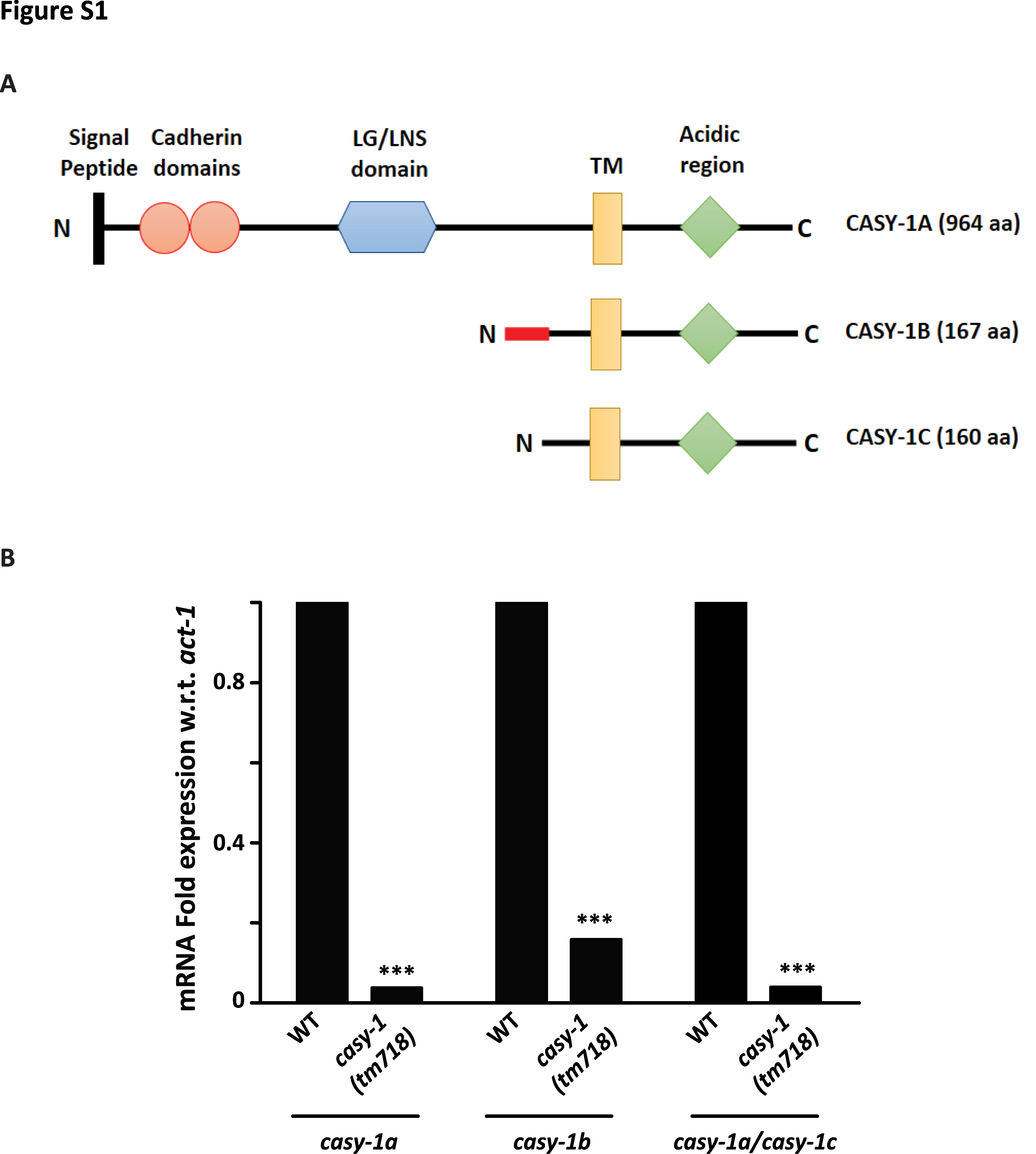

Supplement: S1 Fig — (A) A schematic of the CASY-1 isoforms expressed in C. elegans. CASY-1A is a full-length protein with all the conserved domains- signal peptide (SP), two tandem cadherin domains, LG/LNS domain, transmembrane region and cytosolic acidic region. CASY-1B and CASY-1C are truncated proteins lacking the extracellular LG/LNS domain as well as cadherin repeats. CASY-1B and CASY-1C differ in just 7 amino acids at the N- terminal region, which are extra in CASY-1B. (B) Expression levels of casy-1 isoforms; casy-1a, casy-1b and casy-1c/casy-1a in wild-type (WT) and casy-1(tm718) mutant background in a mixed stage population of C. elegans. Fold change mRNA levels are indicated after normalization with act-1 levels, which served as an internal control. Real time PCR was done thrice from independent RNA samples and the data was analyzed using regular two-way ANOVA in GraphPad Prism V7 software. (TIF) [file pgen.1007263.s001.tif]

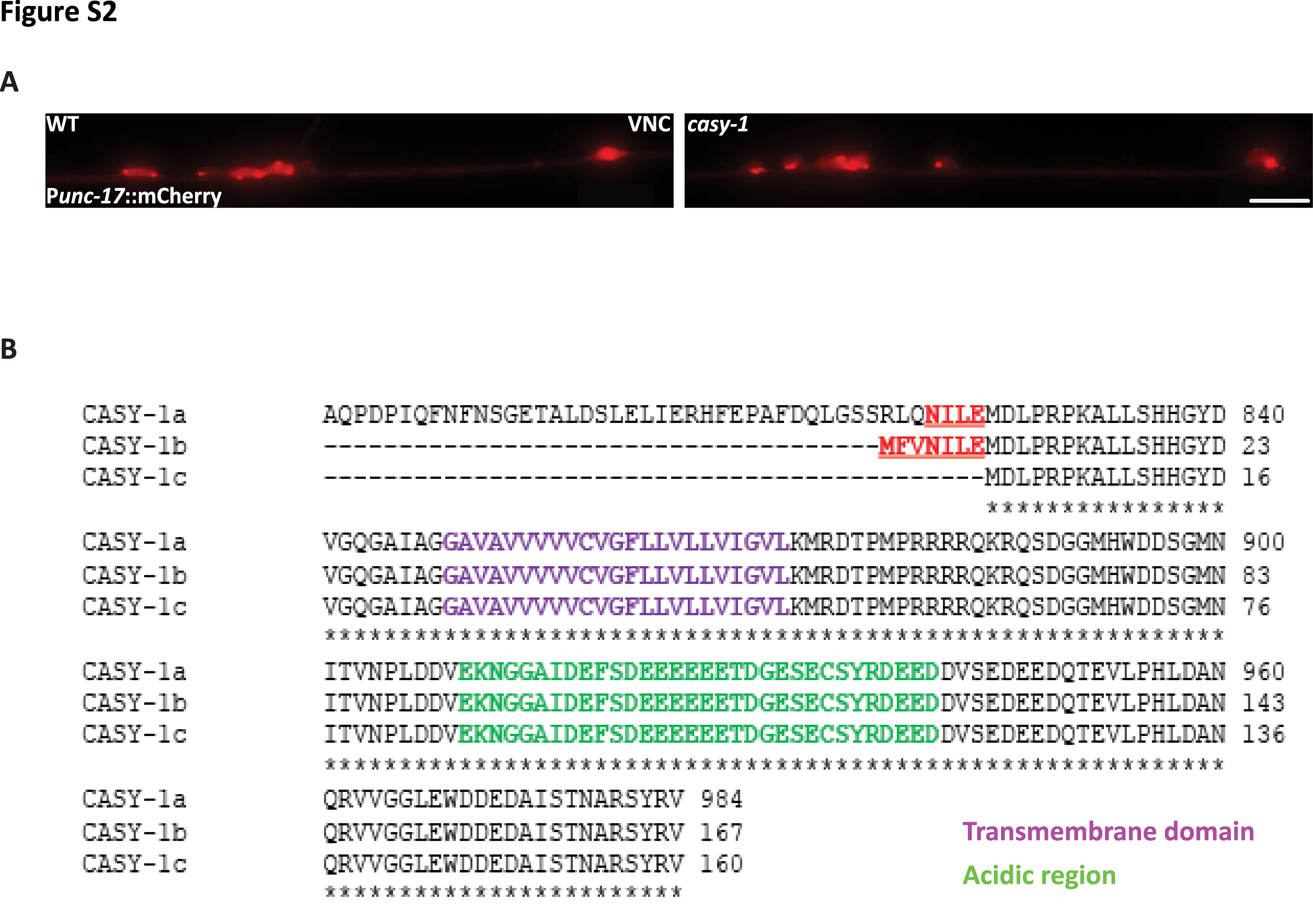

Supplement: S2 Fig — (A) Representative fluorescent images of WT and casy-1 mutant animals expressing mCherry in all cholinergic neurons (nuIs321 [Punc-17::mCherry]). The number of cell bodies and axonal commissures were largely normal in casy-1 mutants (n-28). Scale bar, 8 μm. (B) Clustal omega alignment of the C-terminal of the three casy-1 isoforms. All the isoforms are identical in their C-terminal. Purple and green sequences denote the transmembrane domain and the acidic region in CASY-1 respectively. Red denotes the six amino acids that are present in CASY-1B but not in CASY-1C. (TIF) [file pgen.1007263.s002.tif]

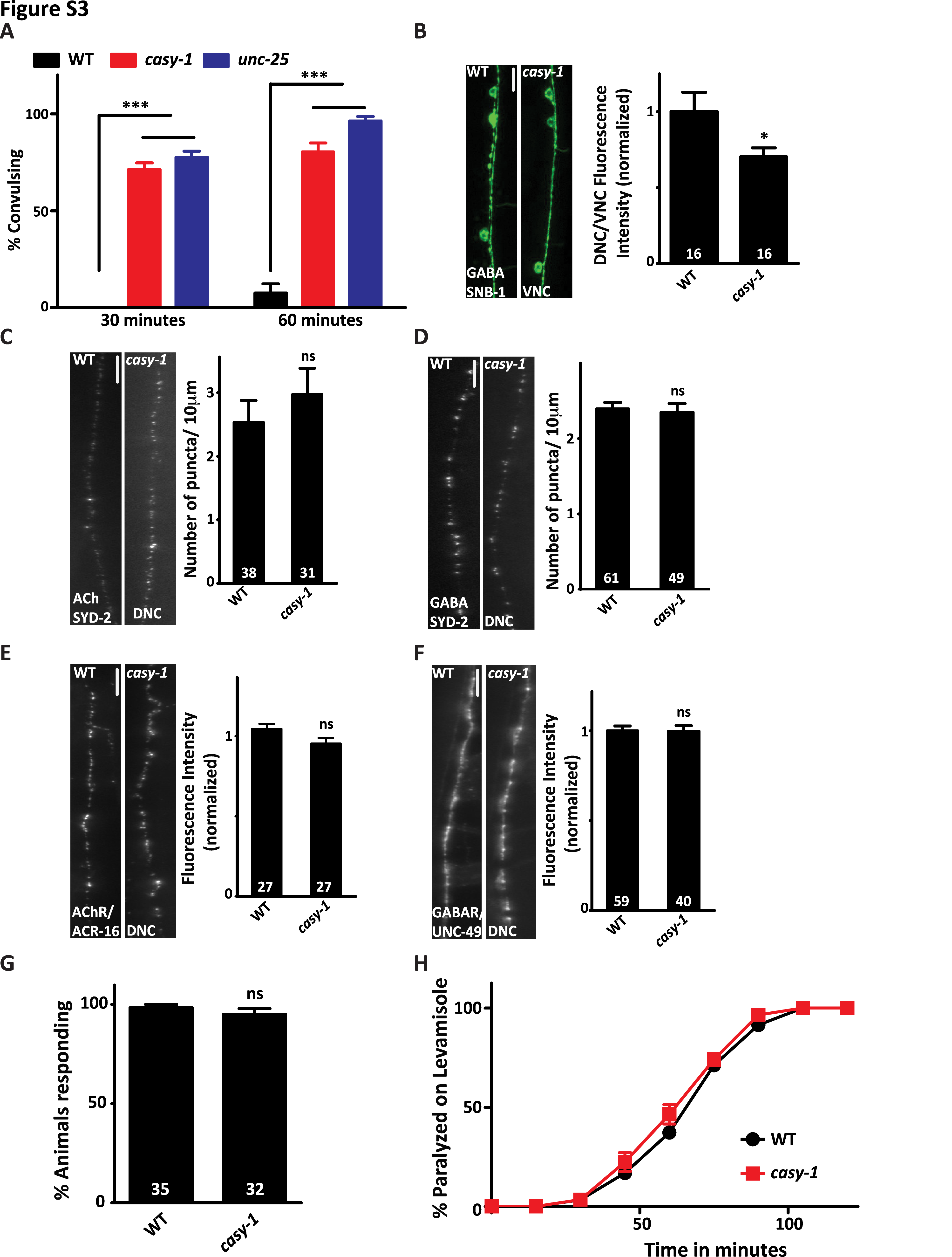

Supplement: S3 Fig — (A) casy-1 mutants show higher sensitivity to GABA receptor antagonist PTZ than the WT animals. The graph shows the fraction of animals showing anterior ‘head bobs’ after 30 minute and 60 minute exposure to 10 mg/ml PTZ. Mutants in unc-25, the GABA synthesis enzyme were used as positive controls. Assays were done (~10 C. elegans/assay) thrice. Data are represented as mean ± S.E.M. (***P<0.0001 using one-way ANOVA and Bonferroni's Multiple Comparison Test). (B) Representative fluorescent images of GABAergic SNB-1::GFP [nuIs376 (Punc-25::SNB-1::GFP)] on the VNC cell bodies of WT or casy-1 mutant. Scale bar, 10μm. The ratio of DNC synapse fluorescence intensity to VNC cell body intensity at GABAergic synapses showed a subtle but significant decrease in fluorescent intensity when compared to WT animals. This suggests that less number of GABA vesicles are present at the DNC synapses. Quantification of fluorescent intensity is normalized to WT values. The number of animals analyzed for each genotype is indicated at the base of the bar graph. Quantified data are displayed as mean ± S.E.M. and were analyzed by two-tailed Student’s t-test (*p<0.01). Representative fluorescent images of cholinergic (C) [nuIs160 (Punc-129:: SYD-2::GFP)] or (D) GABAergic [hpIs3 (Punc-25::SYD-2::GFP)] synapses in the dorsal cord of WT and casy-1 mutant C. elegans. The density of cholinergic and GABAergic synapses was largely normal in the mutants, indicating normal synapse development. Representative fluorescent images of (E) Cholinergic [nuIs299 (Pmyo-3::ACR-16::GFP)] or (F) GABAergic [nuIs283 (Pmyo-3::UNC-49::GFP)] receptors in the muscle of WT and casy-1 mutants. The expression of receptors is unaltered in casy-1 mutants. Quantification of fluorescent intensity is normalized to WT values. The number of animals analyzed for each genotype is indicated at the base of the bar graph. Quantified data are displayed as mean ± S.E.M. and were analyzed by two-tailed Student’s t-test, “ns” indicates not [file pgen.1007263.s003.tif]

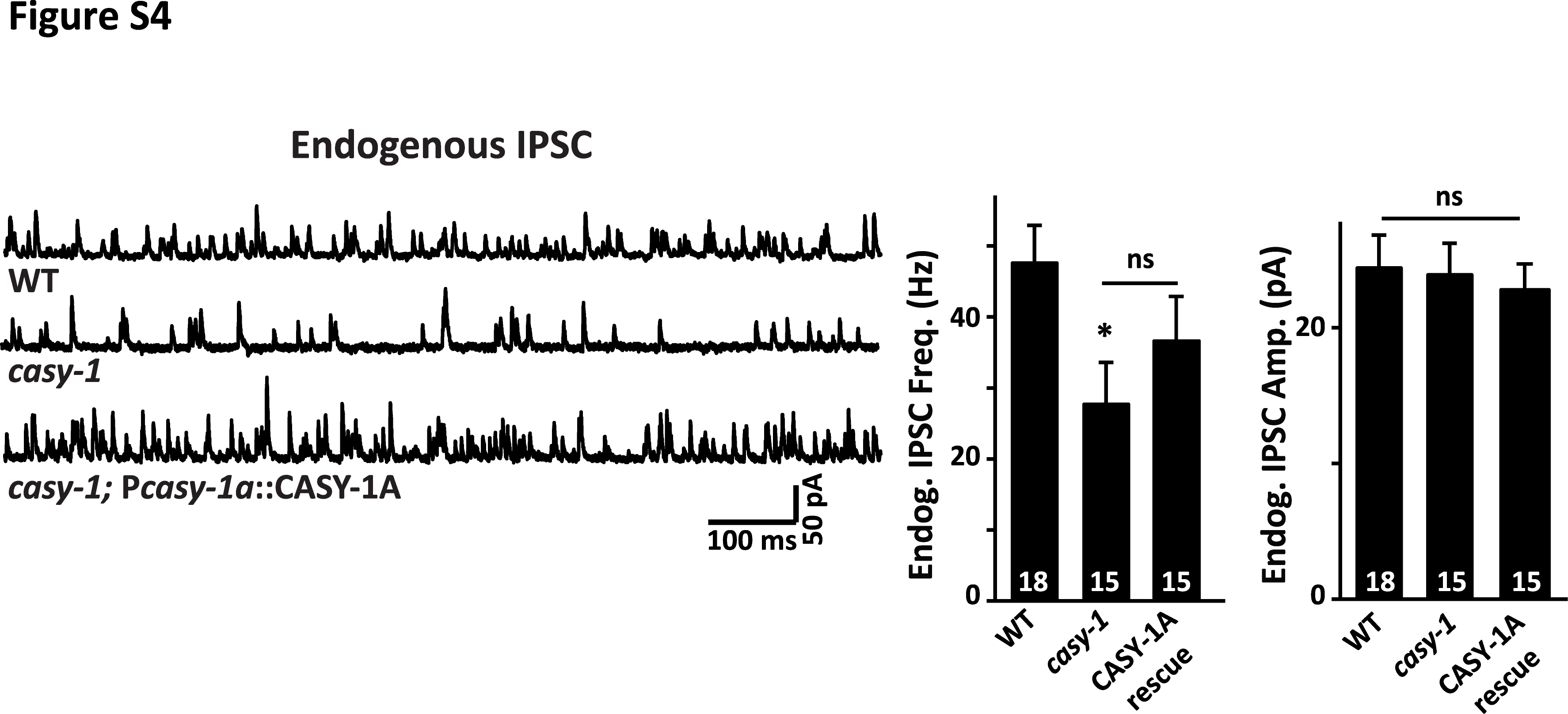

Supplement: S4 Fig — mEPSCs and mIPSCs were recorded from body wall muscles of adult animals for the indicated genotypes. Representative traces of mIPSCs and summary data for frequency and amplitude are shown. Expression of CASY-1A isoform under its endogenous promoter could not rescue the decreased IPSC frequency in casy-1 mutants. This data clearly suggests that CASY-1A isoform do not function to regulate GABA synaptic transmission at the NMJ. The number of animals analyzed for each genotype is indicated. Data are represented as mean ± S.E.M. (*p<0.05 using the one-way ANOVA with Dunnett’s post test, “ns” indicates not significant in all Figures). (TIF) [file pgen.1007263.s004.tif]

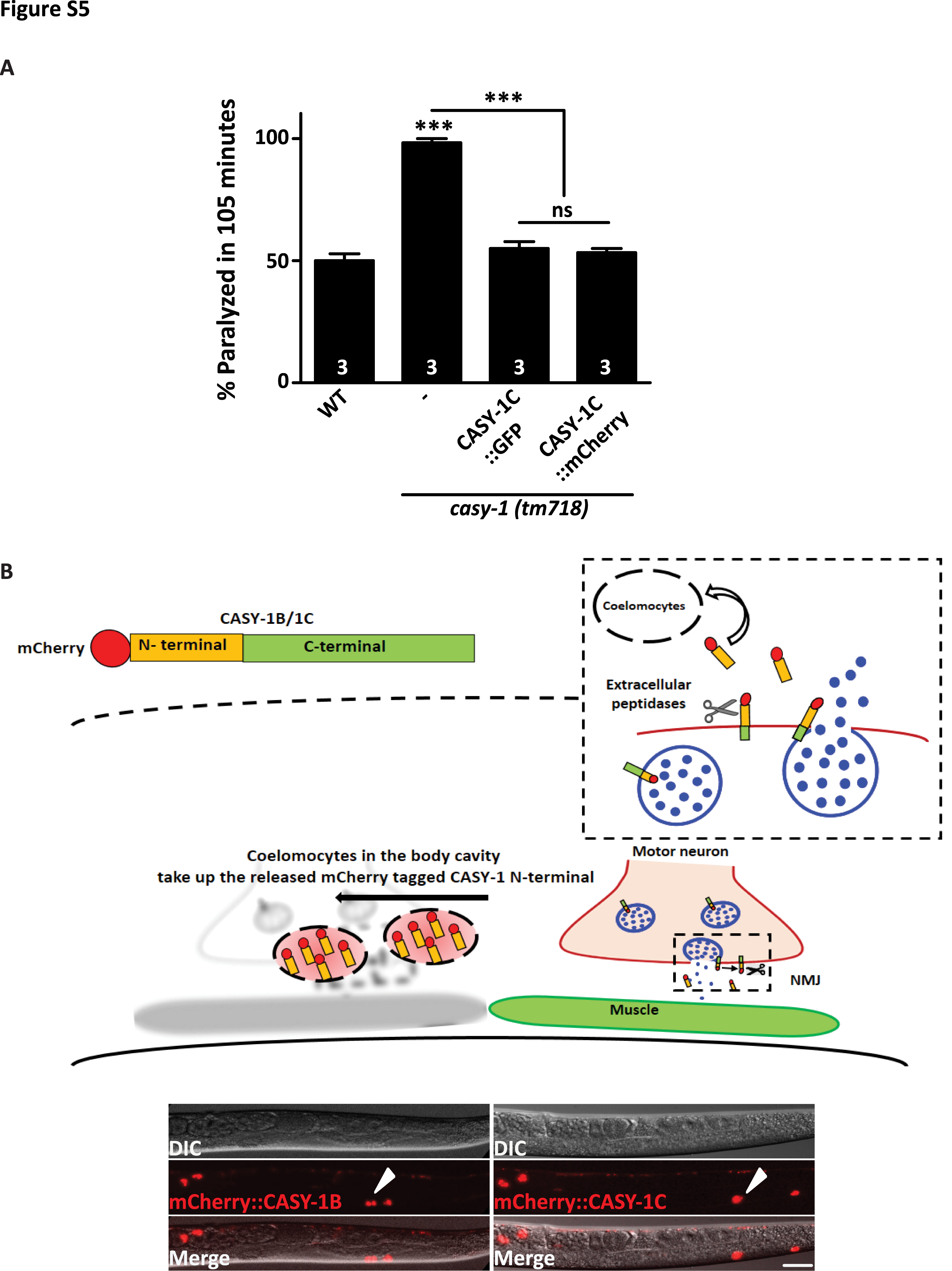

Supplement: S5 Fig — (A) Aldicarb-induced paralysis in casy-1 mutants is completely rescued by expressing GFP or mCherry-tagged CASY-1C expressed under their own promoter, suggesting that the tagged versions of CASY-1C are functional. Assays were done three times as indicated in the figures (~20 C. elegans/assay). Data are represented as mean ± S.E.M. *** indicates p<0.0001 using one-way ANOVA and Bonferroni's Multiple Comparison Test and “ns” indicates not significant in comparison to WT. (B) The upper panel shows a schematic showing the processing of casy-1b and casy-1c isoforms at the NMJ. casy-1a isoform has been shown to be cleaved at its N-terminal by extracellular peptidases resulting in the secretion of entire N- terminal domain [21]. Pcasy-1b/c::mCherry::CASY-1B/C translational reporter shows that CASY-1B and CASY-1C isoforms, which lack a validated signal sequence also reach the synapse where their N- terminal is cleaved resulting in the release of entire N- terminal into the synaptic cleft. The lower panel shows representative fluorescent images showing the release of mCherry tagged N-terminal of CASY-1B and CASY-1C at the synapse followed by their uptake in the coelomocytes (solid arrowheads). Scale bar, 25μm. (TIF) [file pgen.1007263.s005.tif]

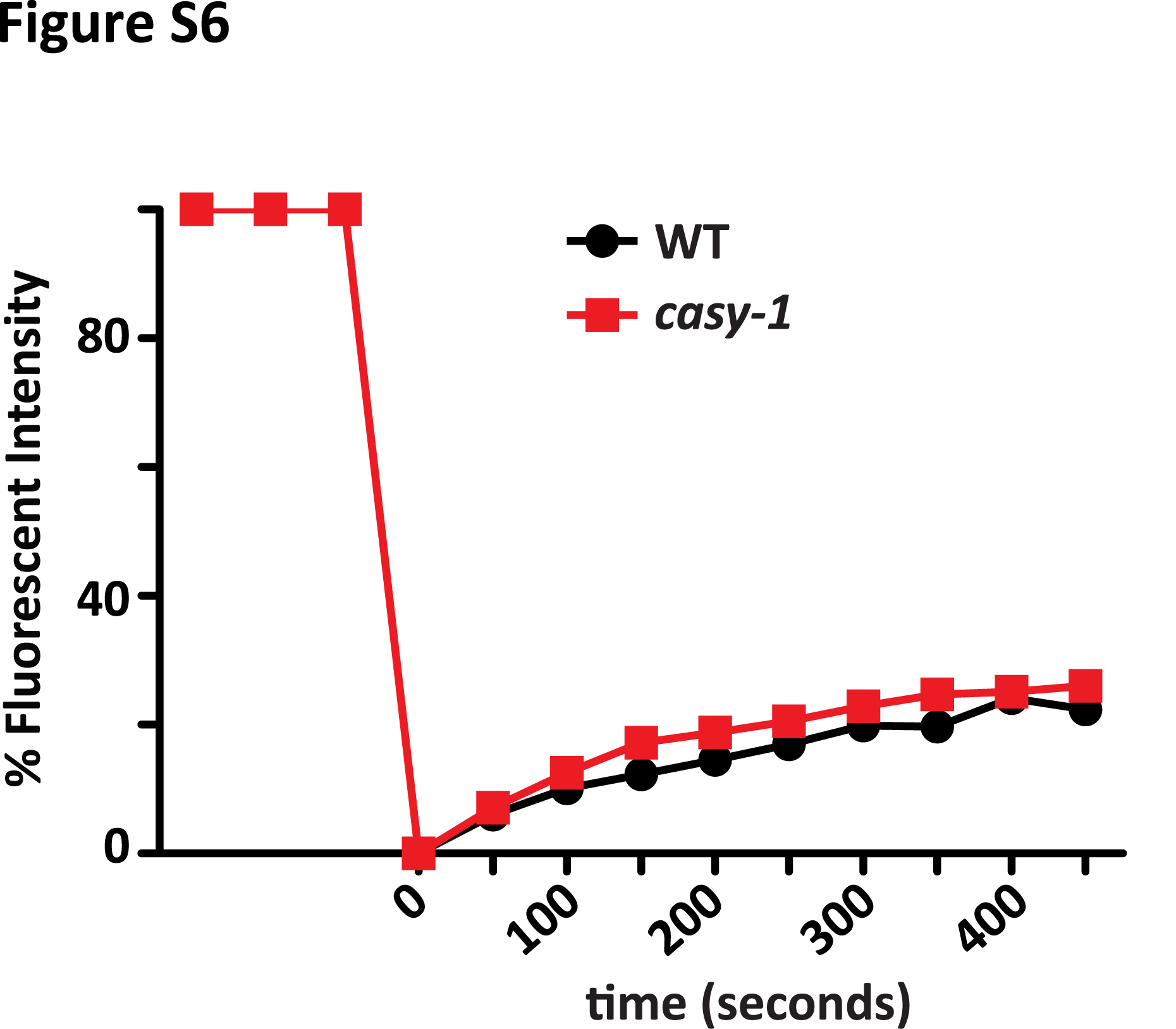

Supplement: S6 Fig — Representative fractional recovery fluorescence trace for a single nuIs152 [Punc-129::SNB-1::GFP] puncta after photobleaching. Recovery was measured with the pre-bleach fluorescence intensity being 100% and the post-bleach intensity at time 0 being 0%. Recovery rate for Cholinergic SNB-1 puncta is unaltered in casy-1 mutant. (TIF) [file pgen.1007263.s006.tif]

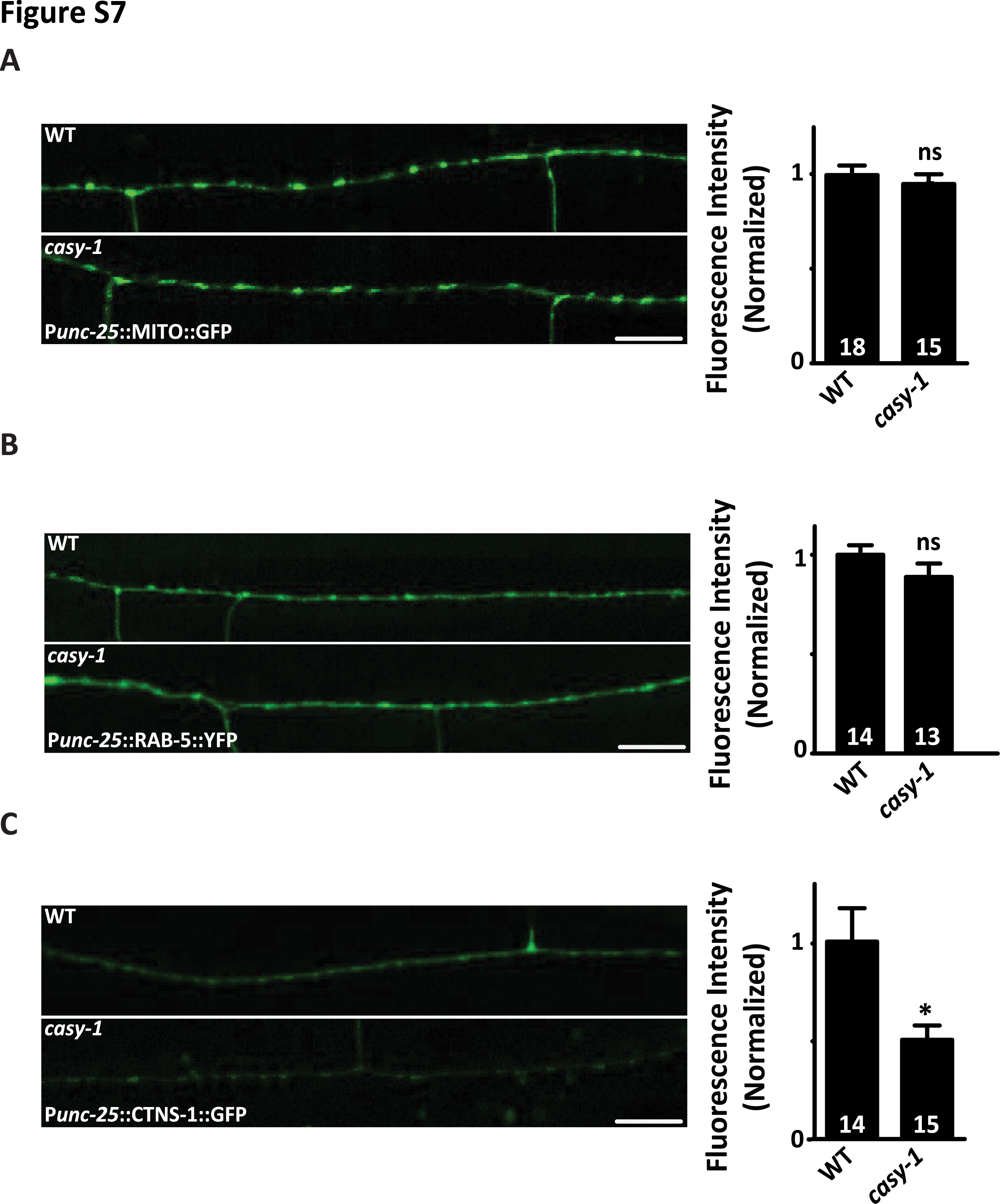

Supplement: S7 Fig — (A) Representative image for Mitochondrial marker (Punc-25::MITO::GFP) in GABAergic motor neurons in WT and casy-1 mutants. (B) Representative image for early endosomal marker [juIs198 (Punc-25:: YFP::RAB-5)] in GABAergic motor neurons in WT and casy-1 mutants. (C) Representative image for Lysosomal marker (Punc-25::CTNS-1::GFP) in GABAergic motor neurons of WT and casy-1 mutants. Scale bar, 10μm. The fluorescence intensity for mitochondrial and early endosomal marker are largely normal in casy-1 mutants, while lysosomal marker showed a subtle but significant decrease in fluorescent intensity when compared to WT animals. Quantification of fluorescent intensity is normalized to WT values. The number of animals analyzed for each genotype is indicated at the base of the bar graph. Quantified data are displayed as mean ± S.E.M. (*p<0.05 using two-tailed Student’s t-test, “ns” indicates not significant in all Figures). (TIF) [file pgen.1007263.s007.tif]

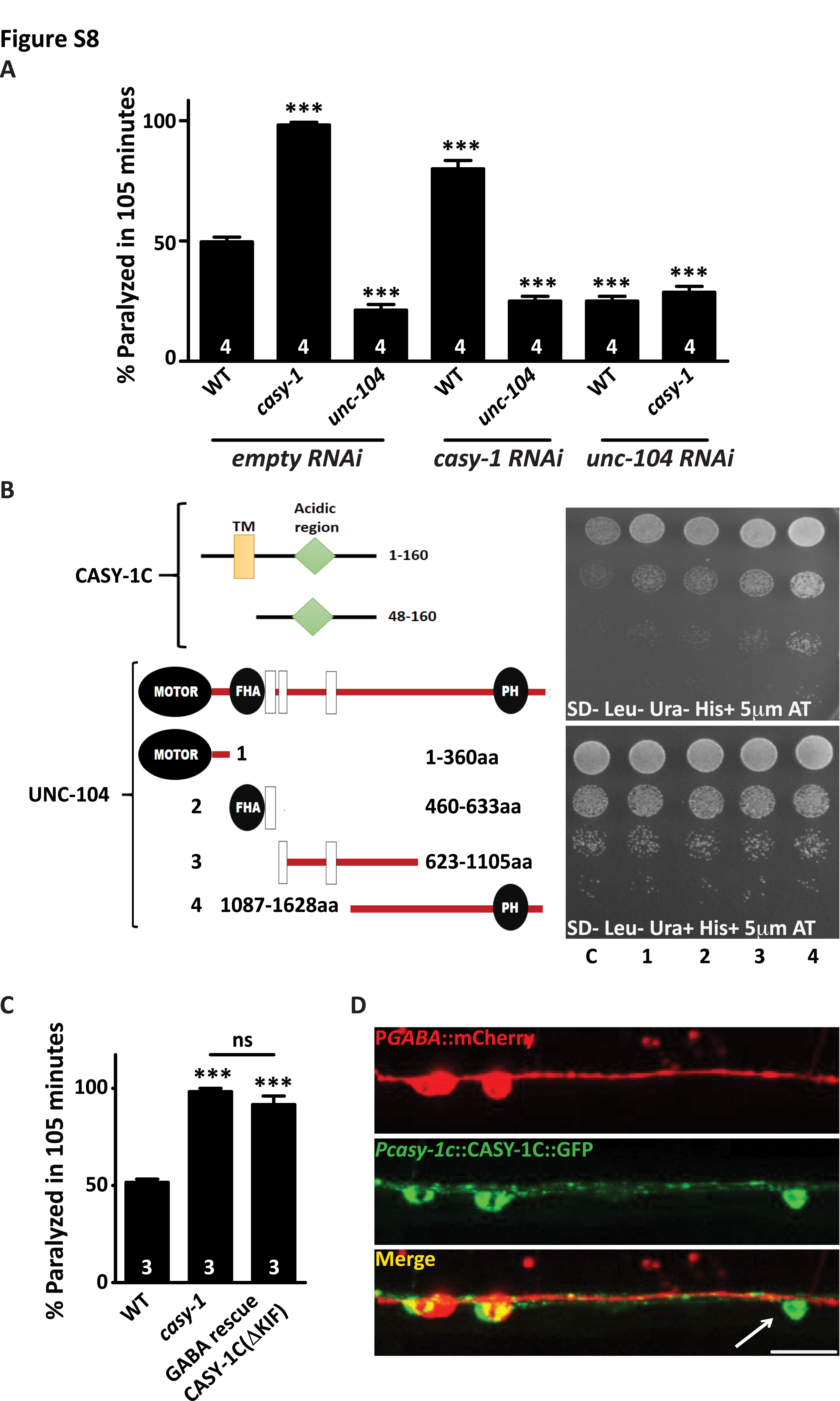

Supplement: S8 Fig — (A) Aldicarb- induced paralysis was compared following RNAi treatments with control (empty vector), unc-104 or casy-1 vectors as indicated in the graph. Knockdown of unc-104 results in a similar Aldicarb resistance phenotype in both the WT and casy-1 mutants indicating that casy-1 and unc-104 could be interacting genetically, however, this could also result from the dominant phenotype of unc-104 function at the synapse. Assays were done 4 times as indicated in Figure (~20 C. elegans/ assay). Data are represented as mean ± S.E.M. (***p<0.0001 using one-way ANOVA and Bonferroni's Multiple Comparison Test). (B) The left panel indicates a schematic representation of CASY-1C C-terminal and UNC-104 constructs utilized in this study. UNC-104 constructs cover several domains; the UNC-104—Motor domain (aa 1–356), the fork head domain FHA (aa 460–633), the SYD-2/liprin binding domain STALK (aa 623–1105) and the PH domain (aa 1087–1628). The lower panel on the right indicates the yeast two hybrid data showing the control spotting in the presence of a selection component while the upper panel shows significant interaction of CASY-1 C terminal with several UNC-104 domains. The most prominent interaction occurs with the tail region of UNC-104 spanning the stalk and PH domain. (C) The C-terminal of the CASY-1C isoform harboring the KIF-interacting domain is required to regulate synaptic transmission at the NMJ. Transgenic lines expressing CASY-1C (ΔKIF) under unc-25 promoter could not rescue the Aldicarb hypersensitivity in casy-1 mutants. Assays were done 3 times as indicated in Figures (~20 C. elegans/ assay). Data are represented as mean ± S.E.M. (***p<0.0001 using one-way ANOVA and Bonferroni's Multiple Comparison Test). “ns” indicates not significant in all Figures. (D) Representative fluorescent images of Pcasy-1c::CASY-1C::GFP colocalizing in the VNC with Punc-25::mCherry in the unc-104 mutant. Colocalization data suggests that the accumulation of CASY-1C::GFP occurs in bot [file pgen.1007263.s008.tif]
