# Supplementary material for: The C-terminal of CASY-1/Calsyntenin regulates GABAergic synaptic transmission at the Caenorhabditis elegans neuromuscular junction
Source: PLoS Genet. 2018 Mar 12;14(3):e1007263. doi: 10.1371/journal.pgen.1007263 (PMC5864096; doi:10.1371/journal.pgen.1007263)
Supplement: S1 Text — (DOCX) [file pgen.1007263.s017.docx]

**S1_Text Supplemental Methods and tables**

**Supplemental methods:**

**Identification of the promoter sequence for each isoform of CASY-1:**

Analysis of the *casy-1* genomic locus revealed that *casy-1b* and *casy-1c* could be expressed by their own internal promoters that lie in the intronic region of *casy-1a*. Choi *et al.* have proposed two pre-requisites to function as an internal promoter both of which are fulfilled for the shorter isoforms of *casy-1* (Fig 1D). First, the introns acting as internal promoters should be significantly longer than the other introns. In the *casy-1a* locus, the 8^th^ intron which could be acting as the internal promoter for *casy-1b* and *casy-1c* is significantly longer than the other introns (approximately 4 kb in size vs 1.5kb for other introns). And second, the internal SL1-coupled introns are more prone to function as independent promoters to produce protein isoforms [1]. The 8^th^ intron in *casy-1a* carries an internal SL1 acceptor leader sequence. To further support our hypothesis, we analyzed the sequence of the *casy-1* gene in closely related nematode, *Caenorhabditis* *briggsae*. *C. briggsae* has just one *casy-1* isoform reported, which is 100% identical in the coding region to the *casy-1a* isoform in *C. elegans* (www.wormbase.org). It carries a 7^th^ intron, which is significantly longer than the other introns (̴ 3700bp). We also did a pairwise sequence alignment for these introns in the two *Caenorhabditis* species using NBLAST (NCBI) and found close to 79% identity in the two sequences, whereas intronic sequences normally show less than 20% identity [2]. This strongly suggests conservation of elements in these intronic sequences. To validate the functionality of the *casy-1a, casy-1b* and *casy-1c* promoters we generated translational fusion lines in which *casy-1* isoforms were expressed under their own specific promoters (3 kb directly upstream of the isoform's initiator methionine) that were used in rescue experiments (Fig 1C).

**Quantitative PCR (qPCR) experiments:**

For Real time qPCR analysis, fresh RNA was isolated from mixed stage populations of wild-type (WT) and *casy-1(tm718)* mutants using the RNeasy Mini Kit (Qiagen). The cDNA was synthesized with random hexamers using the [Transcriptor High Fidelity cDNA Synthesis Kit](https://www.google.co.in/url?sa=t&rct=j&q=&esrc=s&source=web&cd=1&cad=rja&uact=8&ved=0ahUKEwivt-2HtszRAhVEPI8KHcyJAZ8QFggjMAA&url=http%3A%2F%2Fshop.roche.com%2Fshop%2Fproducts%2Ftranscriptor-high-fidelity-cdna-synthesis-kit&usg=AFQjCNHJlY0u2tBu3lCZS9mogrkKuZ7MAw)(Roche). Real time qPCR reactions were carried out in 20µl volumes using the SYBR Premix Ex Taq II (Tli RNase H Plus) (Clontech) in triplicates, *act-1* was used as an internal control and RNA levels of the gene of interest was normalized to *act-1* levels for comparison. The normalized expression of *casy-1* isoforms in *casy-1* mutants relative to WT animals was calculated using the 2^−ΔΔCt^ method. A list of primers used for these experiments is listed in Supplemental Table S5.

**Yeast two-hybrid assay**

All the yeast two-hybrid constructs used in this study are mentioned in Supplemental Table 4. Briefly, pGBDUC1 vector was used as bait containing the yeast URA3 selectable marker and the C-terminal of CASY-1 is expressed as fusion to the Gal4p activation domain. While the pGADC1 vector used as prey carries the LEU2 selection marker and generates protein fusions with Gal4p DNA binding domain. pGAD-C1 and pGBDU-C1 clones containing genes of interest were co-transformed into PJ69-4α using the Lithium acetate method [3] and were then plated onto SC-Ura-Leu plates and incubated at 30°C. Following this, the double transformants were inoculated into 5 mL SD-Ura-Leu and grown overnight in a shaking incubator (New Brunswick, Thermo Scientific) at 200 rpm at 30°C. Cell count was determined by monitoring O.D, followed by spotting serial dilutions of equal number of cells onto SC-Ura-Leu-His and SC-Ura-Leu+His plate and grown for 72 h at 30 °C. For CASY-1 interaction with UNC-104/KIF1A, serial dilutions of equal number of cells were spotted on SC-Ura-Leu-His and SC-Ura-Leu+His plates containing 5 µM 3-Amino-1,2,4-triazole (AT).

For identifying the domain in UNC-104/KIF1A that might be interacting with CASY-1 C- terminal, a series of constructs were generated covering various domain of UNC-104/KIF1A as published previously [4]. These constructs cover several domains in UNC-104/KIF1A - Motor domain (aa 1-356), the forkhead domain FHA (aa 460-633), the SYD-2/Liprin binding domain STALK (aa 623-1105) and the PH domain (aa 1087-1628).

**Supplemental tables**

**Supplemental Table A: Plasmids obtained from other sources or utilized for biochemical assays done in this study**

| Name | Description | Source |
| --- | --- | --- |
| pGBDU-C1 | Yeast-2-hybrid vector-Gal4 DNA Binding Domain | James 1996 [5] |
| pGAD-C1 | Yeast-2-hybrid vector-Gal4 Activation Domain | James 1996 [5] |
| BAB342 | pGBDU-C1 *casy-1* C- terminal acidic region | This study |
| BAB373 | pGAD-C1 UNC-104 (1-360) | This study |
| BAB374 | pGAD-C1 UNC-104 (460-633) | This study |
| BAB375 | pGAD-C1 UNC-104 (623-1105) | This study |
| BAB341 | pGAD-C1 UNC-104 (1087-1628) | This study |
|  | P*unc-25*::MITO::GFP | Sandhya Koushika Lab (unpublished) |
| KG354 | P*unc-25::*CTNS-1A::GFP | [6] |
| pGEX-KG | GST pull down vector- N-terminal GST fusion | This study |
| BAB380 | pGEX-KG CASY-1 C-terminal (48-160) | This study |
| BAB1013 | pGEX-KG CASY-1 C-terminal (48-160) Δkif 970-148) | This study |
| BAB393 | pET-23A UNC-104-HA (1087-1628) | This study |
|  | P*unc-104*::UNC-104::VN173 | [7] |
|  | P*unc-104*::UNC-104::VC155 | [7] |

**Supplemental Table B: List of Plasmids and Arrays generated in this study**

| S. no. | Plasmid | Plasmid number | Array number |
| --- | --- | --- | --- |
| 1 | P*casy-1a*:: mCherry::CASY-1A (injected into *casy-1*) | BAB353 | *IndEx190* |
| 2 | P*casy-1b*::mCherry::CASY-1B (injected into *casy-1*) | BAB354 | *IndEx192* |
| 3 | P*casy-1c*:: mCherry::CASY-1C (injected into *casy-1*) | BAB355 | *IndEx193* |
| 4 | P*casy-1b*::mCherry::CASY-1B (injected into *nuIs376*) | BAB354 | *IndEx1007* |
| 5 | P*casy-1c*::mCherry::CASY-1C (injected into *nuIs376*) | BAB355 | *IndEx1009* |
| 6 | P*casy-1c*::CASY-1C::mCherry (injected into *casy-1*) | BAB363 | *indEx1015* |
| 7 | P*casy-1c*::CASY-1C::GFP  (injected into N2) | BAB384 | *indEx1033* |
| 8 | P*casy-1c*::CASY-1C::GFP  (injected into *casy-1*) | BAB384 | *indEx1034* |
| 9 | P*casy-1b*::NLS-GFP (injected into N2) | BAB351 | *IndEx118* |
| 10 | P*casy-1c*::NLS-GFP (injected into N2) | BAB352 | *IndEx119* |
| 11 | P*unc-17*::CASY-1A (injected into *casy-1*) | BAB364 | *IndEx188* |
| 12 | P*unc-17*::CASY-1B (injected into *casy-1*) | BAB365 | *IndEx181* |
| 13 | P*unc-17*:: CASY-1C (injected into *casy-1*) | BAB366 | *IndEx180* |
| 14 | P*unc-25*::CASY-1A (injected into *casy-1*) | BAB367 | *IndEx189* |
| 15 | P*unc-25*::CASY-1B (injected into *casy-1*) | BAB368 | *IndEx182* |
| 16 | P*unc-25*:: CASY-1C (injected into *casy-1*) | BAB369 | *IndEx184* |
| 17 | P*unc-25*::CASY-1A (ΔCt) (injected into *casy-1*) | BAB350 | *IndEx1011* |
| 18 | P*myo-3*::CASY-1A (injected into *casy-1*) | BAB335 | *IndEx185* |
| 19 | P*unc-30*::CASY-1C(injected into *casy-1*) | BAB383 | *IndEx1038, IndEx1039* |
| 20 | P*unc-25*:: CASY-1C (injected into *casy-1(tm718); zxIs3*) | BAB369 | *IndEx1002, IndEx1012* |
| 21 | P*unc-25*:: CASY-1C (injected into *casy-1(tm718); nuIs376* ) | BAB369 | *IndEx1012* |
| 22 | P*casy-1c*::CASY-1C::mCherry(injected into *nuIs376*) | BAB385 | *IndEx1017* |
| 23 | P*casy-1c*::CASY-1C::mCherry(injected into *nuIs152*) | BAB385 | *IndEx1019* |
| 24 | P*casy-1c*::CASY-1C::GFP (injected into *unc-104(ok913)*) | BAB384 | *IndEx1035* |
| 25 | P*unc-25*::MITO::GFP (injected into N2) |  | *IndEx1069* |
| 26 | P*unc-25::*CTNS-1A::GFP (injected into N2) | KG354 | *IndEx1070* |
| 27 | P*unc-25*::CASY-1C::VC155 (injected into N2) alongwith P*unc-104*::UNC-104::VN | BAB1011 | *IndEx1073* |
| 28 | P*unc-25*::CASY-1C (ΔKIF)::VC155(injected into N2) alongwith P*unc-104*::UNC-104::VN | BAB1010 | *IndEx1074* |
| 29 | P*unc-25*::Empty::VC155 (injected into N2) alongwith P*unc-104*::UNC-104::VN | BAB1006 | *IndEx1072* |
| 30 | P*unc-104*::UNC-104::VC155 VC155 (injected into N2) alongwith P*unc-104*::UNC-104::VN |  | *IndEx1071* |
| 31 | P*unc-25*::mCherry (injected into *IndEx1035*) | BAB1008 | *IndEx1076* |
| 32 | P*unc-25*:: CASY-1C (ΔKIF) (injected into *casy-1*) | BAB1007 | *IndEx1075* |

**Supplemental Table C: List of Integrated lines used in this study**

| S. no. | Plasmid | Integrated Line number | Source and reference |
| --- | --- | --- | --- |
| 1 | P*unc-17*::MCHERY | *nuIs321* | Josh Kaplan Lab [8] |
| 2 | P*unc-25*::GFP | *juIs76* | Yishi Jin Lab [9] |
| 3 | P*unc-129*::SNB-1::GFP | *nuIs152* | Josh Kaplan Lab [10] |
| 4 | P*unc-25*::SNB-1::GFP | *nuIs376* | Josh Kaplan Lab [11] |
| 5 | P*unc-129*::SYD-2::GFP | *nuIs160* | Josh Kaplan Lab [10] |
| 6 | P*unc-25*::SYD-2::GFP | *hpIs3* | CGC |
| 7 | P*myo-3*::ACR-16::GFP | *nuIs299* | Josh Kaplan Lab [8] |
| 8 | P*myo-3*::UNC-49::GFP | *nuIs283* | Josh Kaplan Lab [8] |
| 9 | P*unc*-*25*::mCherry | *IhIs6* | Erik A. Lundquist Lab [12] |
| 10 | Punc-25::snb-1::superecliptic pHluorin; lin15(+)] IV; [lin-15](http://www.wormbase.org/db/get?name=lin-15;class=Gene)([n765ts](http://www.wormbase.org/db/get?name=n765;class=Variation)) | *oxIs155* | Erik Jorgensen Lab [13] |
| 11 | P*casy-1a*::GFP | *sIs10330* | CGC |
| 12 | Punc-25::YFP::RAB-5; Pttx-3::RFP | juIs198 | [14] |

**Supplemental Table D: List of strains**

| S. no. | Genotype | Strain number | Source and reference |
| --- | --- | --- | --- |
| 1 | *casy-1(tm718)* |  | Yuichi Iino Lab [15] |
| 2 | *casy-1 (hd41)* |  | Yuichi Iino Lab [15] |
| 3 | *casy-1(tm718);indEx190* | BAB1068 | This study |
| 4 | *casy-1(tm718);indEx192* | BAB1070 | This study |
| 5 | *casy-1(tm718);indEx193* | BAB1071 | This study |
| 6 | *casy-1(tm718);indEx188* | BAB1066 | This study |
| 7 | *casy-1(tm718);indEx181* | BAB1059 | This study |
| 8 | *casy-1(tm718);indEx180* | BAB1058 | This study |
| 9 | *casy-1(tm718);indEx189* | BAB1067 | This study |
| 10 | *casy-1(tm718);indEx182* | BAB1060 | This study |
| 11 | *casy-1(tm718);indEx184* | BAB1062 | This study |
| 12 | *casy-1(tm718);indEx1011* | BAB1102 | This study |
| 13 | *casy-1(tm718);indEx185* | BAB1063 | This study |
| 14 | *indEx118* | BAB174 | This study |
| 15 | *indEx119* | BAB175 | This study |
| 16 | *casy-1(tm718); nuIs321* | BAB133 | This study |
| 17 | *casy-1(tm718); juIs76* | BAB1046 | This study |
| 18 | *casy-1(tm718); nuIs152* | BAB131 | This study |
| 19 | *casy-1(tm718); nuIs376* | BAB132 | This study |
| 20 | *casy-1(tm718); nuIs160* | BAB164 | This study |
| 21 | *casy-1(tm718); nuIs321* | BAB163 | This study |
| 22 | *casy-1(tm718); nuIs299* | BAB160 | This study |
| 23 | *casy-1(tm718)*; Ex[*hsp16.2p*::*casy-1*, *myo-3*p::venus]. | JN442 | Yuichi Iino lab [15] |
| 24 | *casy-1(tm718)*; Ex[*ins-1p*::*casy-1*(RYV, ΔNt), *myo-3*p::venus] | JN1440 | Yuichi Iino lab [15] |
| 25 | *casy-1(tm718)*; Ex[*ins-1p*::*casy-1*(RYV, delta Ct), myo-3p::venus]. | JN1442 | Yuichi Iino lab [15] |
| 26 | *casy-1(tm718);indEx1038* | BAB1138 | This study |
| 27 | *casy-1(tm718);indEx1039* | BAB1139 | This study |
| 28 | *casy-1(tm718);* [*zxIs3*](http://www.wormbase.org/species/all/transgene/WBTransgene00005248)*; IndEx1001* | BAB1087 | This study |
| 29 | *casy-1(tm718);* [*zxIs3*](http://www.wormbase.org/species/all/transgene/WBTransgene00005248)*; IndEx1002* | BAB1088 | This study |
| 30 | *nuIs321; sIs10330* | BAB176 | This study |
| 31 | *nuIs321; IndEx118* | BAB177 | This study |
| 32 | *nuIs321; IndEx119* | BAB1001 | This study |
| 33 | *IhIs6; sIs10330* | BAB1048 | This study |
| 34 | *IhIs6; IndEx118* | BAB1014 | This study |
| 35 | *IhIs6; IndEx119* | BAB1013 | This study |
| 36 | *IndEx1007* | BAB1098 | This study |
| 37 | *IndEx1009* | BAB1100 | This study |
| 38 | *IndEx1017* | BAB1109 | This study |
| 39 | *unc-104(ok913)* | RB992 | CGC |
| 40 | [*lin-15B*](http://www.wormbase.org/species/c_elegans/gene/WBGene00023497)*&*[*lin-15A*](http://www.wormbase.org/species/c_elegans/gene/WBGene00023498)*(*[*n765*](http://www.wormbase.org/search/variation/n765)*) X;*[*zxIs3*](http://www.wormbase.org/species/all/transgene/WBTransgene00005248) *[[unc-47p::ChR2(H134R)::YFP + lin-15(+)]* | ZX388 | CGC |
| 41 | *casy-1(tm718);* [*zxIs3*](http://www.wormbase.org/species/all/transgene/WBTransgene00005248) | BAB1012 | This study |
| 42 | *casy-1(tm718); oxIs155* | BAB1112 | This study |
| 43 | *IndEx1019* | BAB1111 | This study |
| 44 | *IndEx1017* | BAB1109 | This study |
| 45 | *indEx1033* | BAB1133 | This study |
| 46 | *indEx1035* | BAB1135 | This study |
| 47 | *indEx1036* | BAB1136 | This study |
| 48 | *indEx1037* | BAB1137 | This study |
| 49 | *IndEx1023* | BAB1116 | This study |
| 50 | *IndEx1019* | BAB1111 | This study |
| 51 | *IndEx1069* | BAB1197 | This study |
| 52 | *IndEx1070* | BAB1198 | This study |
| 53 | *casy-1(tm718); IndEx1069* | BAB1199 | This study |
| 54 | *casy-1(tm718);IndEx1070* | BAB1200 | This study |
| 55 | *casy-1(tm718);* juIs198 | BAB1201 | This study |
| 56 | *IndEx1073* | BAB1204 | This study |
| 57 | *IndEx1074* | BAB1205 | This study |
| 58 | *IndEx1072* | BAB1203 | This study |
| 59 | *IndEx1071* | BAB1202 | This study |
| 60 | *IndEx1076* | BAB1208 | This study |
| 61 | *IndEx1075* | BAB1207 | This study |

**Supplemental Table E: Primers utilized for genotyping and Real time PCR**

| Mutant genotyped | Primer number | Primer sequence | Mutation |
| --- | --- | --- | --- |
| *casy-1 (tm718)* | ST150  ST151  ST152 | gacgggtgatggaatgaaag  tcaaagcttctcctcccaga  cagcacgctcctacaacaag | Deletion |
| *casy-1 (hd41)* | \| ST156 \| \| --- \| \| ST157 \| \| ST158 \| | \| tcgtgctggacacgttactc \| \| --- \| \| accatccagcaaaggatacg \| \| ggcgtatgattctggtcgag \| | Deletion |
| *unc-104(ok913)* | \| ST415  ST416  ST417 \| \| --- \| | \| cgaaggaaataaagcgagg \| \| --- \| \| cgacggtcggtggctgttcgg \| \| ctcttcatctctatcgagaacc \| | Deletion |
| *lin-15b(n744)* | ST176  ST177  ST175 | gtctgacgcattccccac  gtctgacgcattccccat  cattgtcgagagctcagc | Substitution |
| *eri-1(mg366)* | ST173  ST588  ST174 | aaacttcggaacatatggggc  ttcgataaagtgcctgttttttt  tgggtaaggaatcgaagacg | Deletion |
| *casy-1a* | ST408  ST152 | ggttgttctccgccgtgaagctgg  cagcacgctcctacaacaag | Real time PCR |
| *casy-1b* | ST411  ST244 | atgttcgtgaacattctgg  tcccactcaagacctccaac | Real time PCR |
| *casy-1c* | ST241  ST244 | cggtggtgcaattgatgagt  tcccactcaagacctccaac | Real time PCR |
| *act-1* | ST066  ST067 | tgcgacattgatatccgtaagg  ggtggttcctccggaaagaa | Real time PCR |

**Supplemental Table F: Primers utilized for cloning (**FP denotes forward primer and RP, reverse primer)

| Gene/ Promoter cloned | Primer no. | Forward/  Reverse primer | Primer Sequence | Vector backbone |
| --- | --- | --- | --- | --- |
| *casy-1a* promoter | ST331  ST332 | FP  RP | ctctctcctgcaggccgtacttcctctgaatcgac  ctctctggatccaatggtgatgtttggcgtaggagacgcc | pPD49.26 |
| *casy-1b* promoter | ST324  ST325 | FP  RP | ctctctctgcaggagtcgagcactcctgacaccc  ctctctggatccgcagtgaaataagtgtataaagaag | pPD49.26 |
| *casy-1c* promoter | ST328  ST329 | FP  RP | ctctctcctgcaggctgaaaatctcttcactatacaacccg  ctctctggatccctaaaagtataaacaattagattatgac | pPD49.26 |
| *casy-1a* gene (N- terminal MCHERRY fusion) | ST334  ST335 | FP  RP | ctctctcccggggctgctcgagcgccaataatcaatctgc  ctctctgctagcgacacgataagaacgagcgttcg | pPD49.26 |
| *casy-1b* gene (N- terminal MCHERRY fusion) | ST357  ST335 | FP  RP | ctctctcccgggttcgtgaacattctggaaatggacc  ctctctgctagcgacacgataagaacgagcgttcg | pPD49.26 |
| *casy-1c* gene (N- terminal MCHERRY fusion) | ST288  ST335 | FP  RP | ctctctcctgcaggctgaaaatctcttcactatacaacccg  ctctctgctagcgacacgataagaacgagcgttcg | pPD49.26 |
| MCHERRY (N- terminal fusion) | ST404  ST406 | FP  RP | ctctctggatccatggtctcaaagggtgaagaag  ctctctcccgggcttatacaattcatccatgcc | pPD49.26 |
| MCHERRY (C- terminal fusion) | ST609  ST610 | FP  RP | ctctctgctagcgtctcaaagggtgaagaag  ctctctggtaccctacttatacaattcatccat | pPD49.26 |
| GFP (C- terminal fusion) | ST552  ST553 | FP  RP | ctctctggtaccagtaaaggagaagaacttttc  ctctctcggccgctatttgtatagttcatcc | pPD49.26 |
| *casy-1a* gene (C- terminal MCHERRY/GFP fusion) | ST599  ST597 | FP  RP | ctctctggatccgctgctcgagcgccaataa  ctctctaccggtttgacacgataagaacg | pPD49.26 |
| *casy-1b* gene (C- terminal MCHERRY/GFP fusion) | ST596  ST597 | FP  RP | ctctctggatccatgttcgtgaacattctgg  ctctctaccggtttgacacgataagaacg | pPD49.26 |
| *casy-1c* gene (C- terminal MCHERRY/GFP fusion) | ST598  ST597 | FP  RP | ctctctggatccatggacctcccgcgtcc  ctctctaccggtttgacacgataagaacg | pPD49.26 |
| *casy-1b* promoter transcriptional reporter line | ST305  ST306 | FP  RP | ctctct*cctgcagg*cgacggagtcaccactaccacatccacga  ctctctggatccctggaatacaagtatcaacttcagaattctg | pGC76 |
| *casy-1c* promoter transcriptional reporter line | ST307  ST308 | FP  RP | ctctctcctgcaggctgaaaatctcttcactatacaacccg  ctctctggatccccagaatgttctaaaagtataaacaattagattatgac | pGC76 |
| *unc-17* promoter (cholinergic rescue) | ST363  ST318 | FP  RP | ctctctaccggtcatgacaaagtggtgacactgg  ctctctcctgcagggactccaccgagttaccttaaa | pCFJ910 (later sub cloned into pPD49.26) |
| *unc-25* promoter (GABAergic rescue) | ST373  ST374 | FP  RP | ctctctaagcttggtcaaaagccgaaatttaaagctag  ctctctggatccgtggctcagttgtgtagttgct | pPD49.26 |
| *myo-3* promoter (muscle rescue) | ST466  ST467 | FP  RP | ctctctggatccagtgattatagtctctgttttcg  ctctctgctagccatttctagatggatctagtgg | pPD49.26 |
| *unc-30* promoter (DD/VD neuron rescue) | ST621  ST622 | FP  RP | ctctctctgcagcgccattcggagcacgctcttcagc  ctctctggatccgccggagggcgcctcaatcccc | pPD49.26 |
| CASY-1A gene (for rescue) | ST336  ST162 | FP  RP | ctctctggtaccatgcgaactgcgtactttatttttgtcg  ctctctactagtggggaaggagtgaaaaggac | pPD49.26 |
| CASY-1B gene (for rescue) | ST337  ST162 | FP  RP | ctctctggtaccatgttcgtgaacattctgg  ctctctactagtggggaaggagtgaaaaggac | pPD49.26 |
| CASY-1C gene (for rescue) | ST338  ST162 | FP  RP | ctctctggtaccatggacctcccgcgtccg  ctctctactagtggggaaggagtgaaaaggac | pPD49.26 |
| CASY-1A truncated (ΔCt) (for rescue) | ST336  ST502 | FP  RP | ctctctggtaccatgcgaactgcgtactttatttttgtcg  ctctctactagtttatggcattggggtgtctcgc | pPD49.26 |
| *unc-104* RNAi | ST443  ST444 | FP  RP | ctctctaccggttcctgaacacatgcaagagg  ctctctaagcttcaacagctggttgagcacat | L4440 |
| *casy-1* RNAi | ST146  ST147 | FP  RP | ctctctactagtcggcaagagcactctcttct  ctctctggtaccagacccttttctcgcaatcc | L4440 |
| CASY-1 C- terminal minus acidic region (for yeast two hybrid assay) | ST447  ST486 | FP  RP | ctctctggatccaaaatgcgagacaccccaatgc  ctctctgtcgacatcactgaactcatcaattgcacc | pGBDUC1 |
| UNC-104 (1-360) (for yeast two hybrid assay) | ST449  ST611 | FP  RP | ctctctcccgggtcatcggttaaagtagctgtacg  ctctctctgcagctattttgcatttggatcctc | pGADC1 |
| UNC-104 (460-633) (for yeast two hybrid assay) | ST612  ST613 | FP  RP | ctctctcccgggagtccaaagaagttacccc  ctctctctgcagctaatattcccttgtctgatg | pGADC1 |
| UNC-104 (623-1105) (for yeast two hybrid assay) | ST614  ST615 | FP  RP | ctctctcccggggaacaaaaaatgtatcatc  ctctctctgcagctaccacacaagaagatcatg | pGADC1 |
| UNC-104 (1087-1628) (for yeast two hybrid assay) | ST481  ST450 | FP  RP | ctctctcccggggcaccaattcagaacaataacgc  ctctctctgcagttatgaagcagcaattgaagatg | pGADC1 |
| CASY-1C (ΔKIF) (for Aldicarb assay) | ST161  ST683  ST684  ST162 | FP  IRP  EFP  RP | ctctctgggcccatggacctcccgcgtccgaaagcac  gagatggcgtcatgcattcctccgtcac  ggaatgcatgacgccatctcaacgaacg  ctctctactagtggggaaggagtgaaaaggac | pPD49.26 |
| CASY-1C (ΔKIF) (for GST Pull down) | ST447  ST683  ST684  ST618 | FP  IRP  EFP  RP | ctctctggatccaaaatgcgagacaccccaatgc  gagatggcgtcatgcattcctccgtcac  ggaatgcatgacgccatctcaacgaacg  ctctctgtcgacttacagatcctcttctgagatgagtttttgttcgacacgataagaa | pGEX-KG |
| CASY-1C (ΔKIF) (for BiFC assay) | ST666  ST667  VT185  VT186 | FP  RP (with linker)  FP (with linker)  RP | ctctctggatccatggacctcccgcgtccgaaagc  atggttcatcactttctgtttcagatcgttcggaattttgcacgccgggcggacacgataagaacgagcgttcg  cgcccggcgtgcaaaattccgaacgatctgaaacagaaa  gtgatgaaccatcagaagaacggcatcaaggcc  ctagctagcttacttgtacagctcgtccatgccg | P*unc-25*:: pPD49.26 |
| CASY-1C (for GST Pull down) | ST447  ST618 | FP  RP | ctctctggatccaaaatgcgagacaccccaatgc  ctctctgtcgacttacagatcctcttctgagatgagtttttgttcgacacgataagaa | pGEX-KG |
| CASY-1C::VC155 | ST666  ST667  VT185  VT186 | FP  RP (with linker)  FP (with linker)  RP | ctctctggatccatggacctcccgcgtccgaaagc  atggttcatcactttctgtttcagatcgttcggaattttgcacgccgggcggacacgataagaacgagcgttcg  cgcccggcgtgcaaaattccgaacgatctgaaacagaaa  gtgatgaaccatcagaagaacggcatcaaggcc  ctagctagcttacttgtacagctcgtccatgccg | P*unc-25*:: pPD49.26 |
| Empty::VC155 | ST672  ST671  VT185  VT186 | FP  RP  FP (with linker)  RP | ctctctggatcccgcccggcgtgcaaaattccgaacg  ctctctggtaccttacttgtacagctcgtccatgccg  cgcccggcgtgcaaaattccgaacgatctgaaacagaaa  gtgatgaaccatcagaagaacggcatcaaggcc  ctagctagcttacttgtacagctcgtccatgccg | P*unc-25*:: pPD49.26 |
| Superecliptic  pHluorin | \| ST606 \| \| --- \| \| ST607 \| | FP  RP | \| catacggaaaacttaccc \| \| --- \| \| gttgaacgcctccatcttc \| | pPD49.26 |

**Reference:**

1. Choi J, Newman AP. A two-promoter system of gene expression in C. elegans. Developmental biology. 2006;296(2):537-44. doi: 10.1016/j.ydbio.2006.04.470. PubMed PMID: 16765937.

2. Shabalina SA, Kondrashov AS. Pattern of selective constraint in C. elegans and C. briggsae genomes. Genet Res. 1999;74(1):23-30. PubMed PMID: 10505405.

3. Gietz RD, Schiestl RH. Quick and easy yeast transformation using the LiAc/SS carrier DNA/PEG method. Nat Protoc. 2007;2(1):35-7. doi: 10.1038/nprot.2007.14. PubMed PMID: 17401335.

4. Wagner OI, Esposito A, Kohler B, Chen CW, Shen CP, Wu GH, et al. Synaptic scaffolding protein SYD-2 clusters and activates kinesin-3 UNC-104 in C. elegans. Proceedings of the National Academy of Sciences of the United States of America. 2009;106(46):19605-10. doi: 10.1073/pnas.0902949106. PubMed PMID: 19880746; PubMed Central PMCID: PMCPMC2780759.

5. James P, Halladay J, Craig EA. Genomic libraries and a host strain designed for highly efficient two-hybrid selection in yeast. Genetics. 1996;144(4):1425-36. PubMed PMID: 8978031; PubMed Central PMCID: PMCPMC1207695.

6. Edwards SL, Yu SC, Hoover CM, Phillips BC, Richmond JE, Miller KG. An organelle gatekeeper function for Caenorhabditis elegans UNC-16 (JIP3) at the axon initial segment. Genetics. 2013;194(1):143-61. doi: 10.1534/genetics.112.147348. PubMed PMID: 23633144; PubMed Central PMCID: PMCPMC3632462.

7. Wu GH, Muthaiyan Shanmugam M, Bhan P, Huang YH, Wagner OI. Identification and Characterization of LIN-2(CASK) as a Regulator of Kinesin-3 UNC-104(KIF1A) Motility and Clustering in Neurons. Traffic. 2016;17(8):891-907. doi: 10.1111/tra.12413. PubMed PMID: 27172328.

8. Babu K, Hu Z, Chien SC, Garriga G, Kaplan JM. The immunoglobulin super family protein RIG-3 prevents synaptic potentiation and regulates Wnt signaling. Neuron. 2011;71(1):103-16. Epub 2011/07/13. doi: S0896-6273(11)00485-5 [pii]

10.1016/j.neuron.2011.05.034. PubMed PMID: 21745641; PubMed Central PMCID: PMC3134796.

9. Baran R, Castelblanco L, Tang G, Shapiro I, Goncharov A, Jin Y. Motor neuron synapse and axon defects in a C. elegans alpha-tubulin mutant. PloS one. 2010;5(3):e9655. doi: 10.1371/journal.pone.0009655. PubMed PMID: 20300184; PubMed Central PMCID: PMCPMC2836382.

10. Sieburth D, Ch'ng Q, Dybbs M, Tavazoie M, Kennedy S, Wang D, et al. Systematic analysis of genes required for synapse structure and function. Nature. 2005;436(7050):510-7. PubMed PMID: 16049479.

11. Hao Y, Hu Z, Sieburth D, Kaplan JM. RIC-7 promotes neuropeptide secretion. PLoS genetics. 2012;8(1):e1002464. doi: 10.1371/journal.pgen.1002464. PubMed PMID: 22275875; PubMed Central PMCID: PMCPMC3261915.

12. Norris AD, Lundquist EA. UNC-6/netrin and its receptors UNC-5 and UNC-40/DCC modulate growth cone protrusion in vivo in C. elegans. Development. 2011;138(20):4433-42. doi: 10.1242/dev.068841. PubMed PMID: 21880785; PubMed Central PMCID: PMCPMC3177313.

13. Ernstrom GG, Weimer R, Pawar DR, Watanabe S, Hobson RJ, Greenstein D, et al. V-ATPase V1 sector is required for corpse clearance and neurotransmission in Caenorhabditis elegans. Genetics. 2012;191(2):461-75. doi: 10.1534/genetics.112.139667. PubMed PMID: 22426883; PubMed Central PMCID: PMCPMC3374311.

14. Sann SB, Crane MM, Lu H, Jin Y. Rabx-5 regulates RAB-5 early endosomal compartments and synaptic vesicles in C. elegans. PloS one. 2012;7(6):e37930. doi: 10.1371/journal.pone.0037930. PubMed PMID: 22675499; PubMed Central PMCID: PMCPMC3366993.

15. Ikeda DD, Duan Y, Matsuki M, Kunitomo H, Hutter H, Hedgecock EM, et al. CASY-1, an ortholog of calsyntenins/alcadeins, is essential for learning in Caenorhabditis elegans. Proceedings of the National Academy of Sciences of the United States of America. 2008;105(13):5260-5. doi: 10.1073/pnas.0711894105. PubMed PMID: 18381821; PubMed Central PMCID: PMC2278220.
