# Supplementary figures and images for: Correction: The C-terminal of CASY-1/Calsyntenin regulates GABAergic synaptic transmission at the Caenorhabditis elegans neuromuscular junction
Source: PLoS Genet. 2026 Jan 20;22(1):e1012023. doi: 10.1371/journal.pgen.1012023 (PMC12818617; doi:10.1371/journal.pgen.1012023)

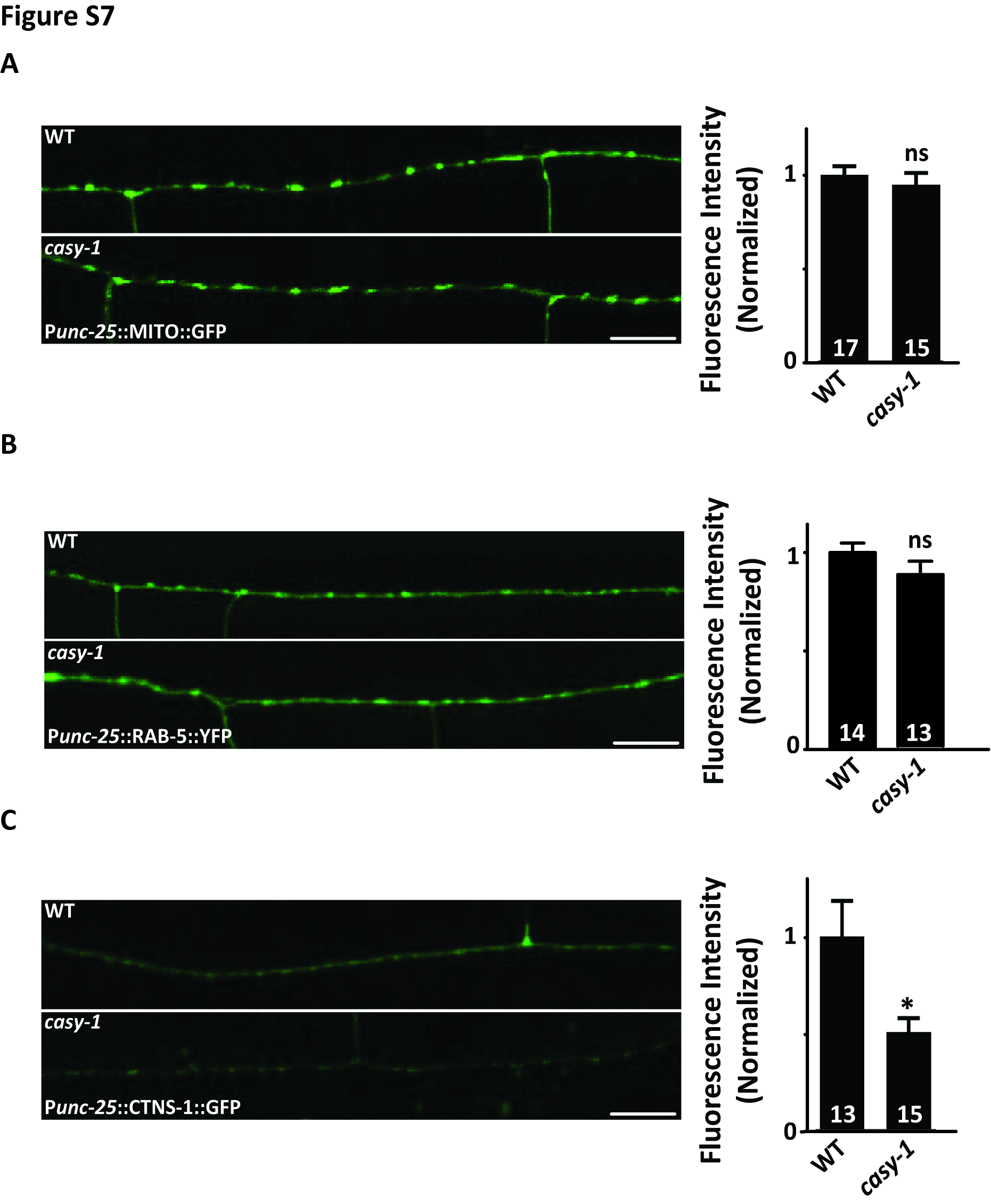

Supplement: S7 Fig — (B) Representative image for early endosomal marker [juIs198 (Punc-25:: YFP::RAB-5)] in GABAergic motor neurons in WT and casy-1 mutants. (C) Representative image for Lysosomal marker (Punc-25::CTNS-1::GFP) in GABAergic motor neurons of WT and casy-1 mutants. Scale bar, 10μm. The fluorescence intensity for mitochondrial and early endosomal marker are largely normal in casy-1 mutants, while lysosomal marker showed a subtle but significant decrease in fluorescent intensity when compared to WT animals. Quantification of fluorescent intensity is normalized to WT values. The number of animals analyzed for each genotype is indicated at the base of the bar graph. Quantified data are displayed as mean ± S.E.M. (*p < 0.05 using two-tailed Student’s t-test, “ns” indicates not significant in all Figures). (TIF) [file pgen.1012023.s001.tif]

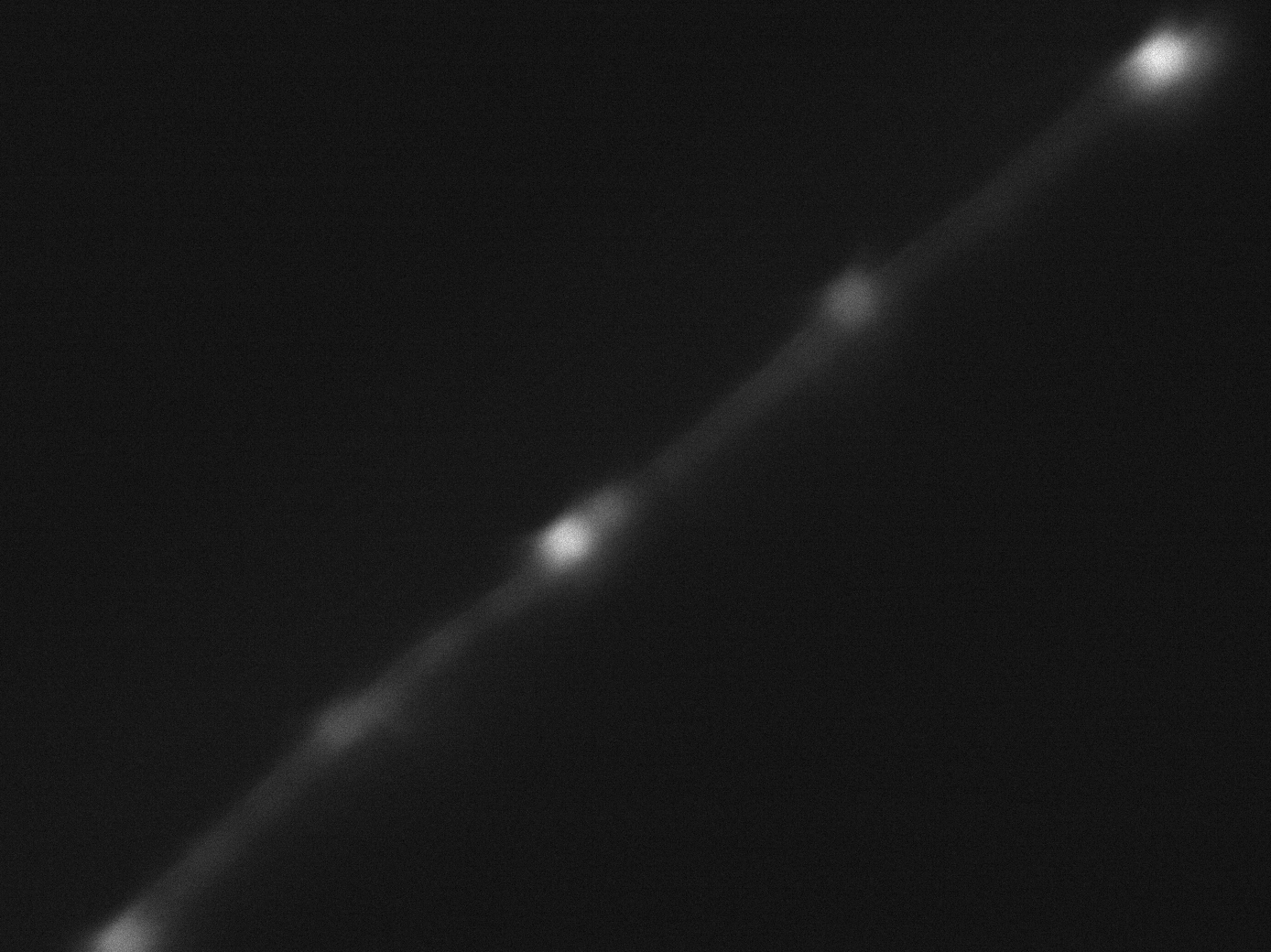

Supplement: S2 File — This file includes the original images underlying Fig 2A. Each image file included in S2 File is generated from image stacks taken in the original experiments. (ZIP) [file pgen.1012023.s003.zip › S2 File/Fig 2A Punc25_GFP casy-1 VNC.tif]

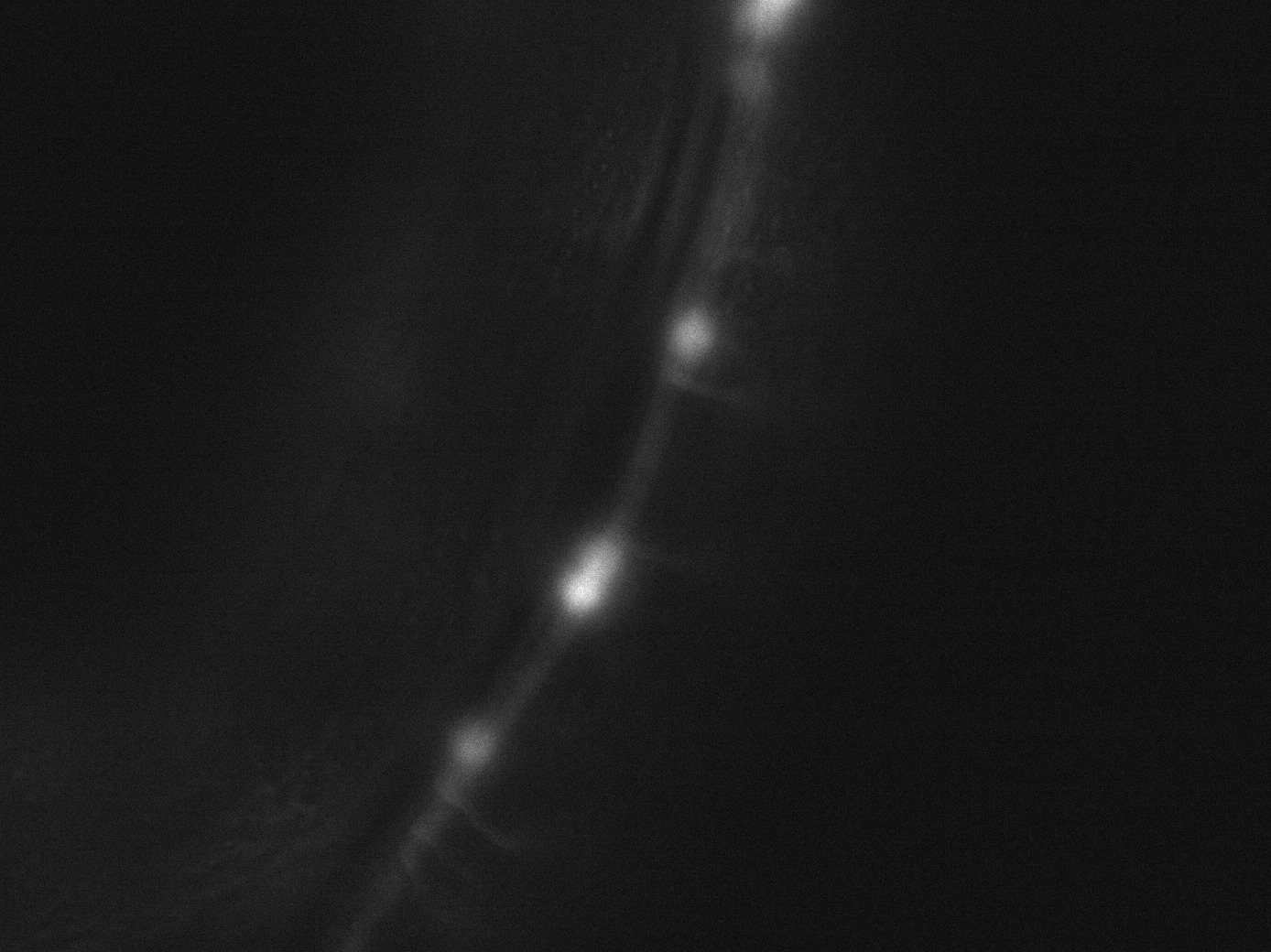

Supplement: S2 File — This file includes the original images underlying Fig 2A. Each image file included in S2 File is generated from image stacks taken in the original experiments. (ZIP) [file pgen.1012023.s003.zip › S2 File/Fig 2A Punc25_GFP WT VNC.tif]

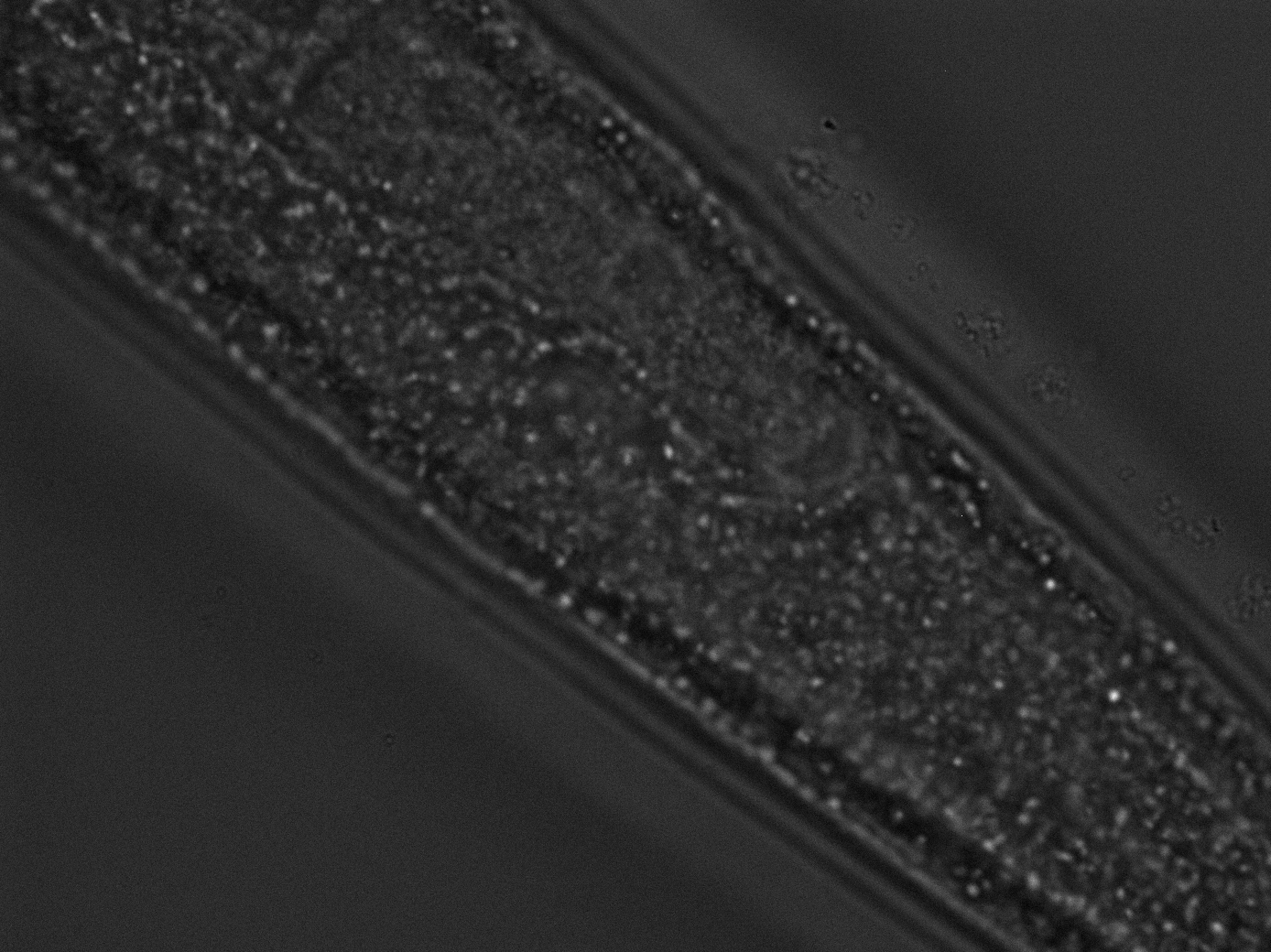

Supplement: S4 File — This file includes the original images underlying S2A Fig. Each image file included in S4 File is generated from image stacks taken in the original experiments. (ZIP) [file pgen.1012023.s005.zip › S4 File/Fig S2A Punc17_mCherry casy-1 VNC.tif]

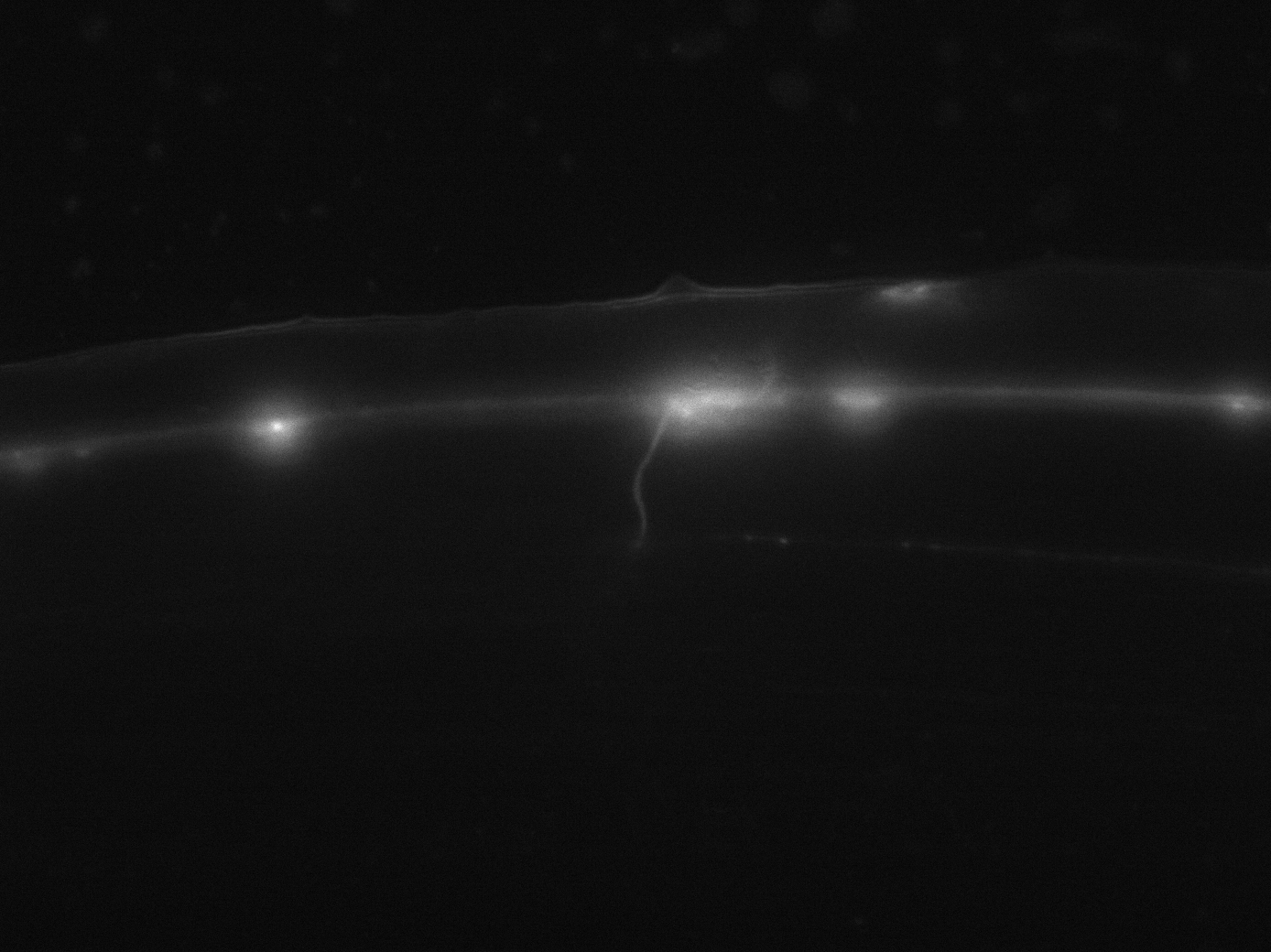

Supplement: S4 File — This file includes the original images underlying S2A Fig. Each image file included in S4 File is generated from image stacks taken in the original experiments. (ZIP) [file pgen.1012023.s005.zip › S4 File/Fig S2A Punc17_mCherry WT VNC.tif]
